# Molecular impact of nicotine and smoking exposure on the developing and adult mouse brain

**DOI:** 10.1016/j.addicn.2026.100270

**Published:** 2026-07-15

**Authors:** Daianna Gonzalez-Padilla, Nicholas J. Eagles, Marisol Cano, Geo Pertea, Andrew E. Jaffe, Kristen R. Maynard, Dana B. Hancock, James T. Handa, Keri Martinowich, Leonardo Collado-Torres

**Affiliations:** a Lieber Institute for Brain Development, Johns Hopkins Medical Campus, Baltimore, MD, 21205, USA; b Wilmer Eye Institute, Johns Hopkins School of Medicine, Baltimore, MD, 21287, USA; c The Solomon H. Snyder Department of Neuroscience, Johns Hopkins School of Medicine, Baltimore, MD, 21205, USA; d Department of Psychiatry and Behavioral Sciences, Johns Hopkins School of Medicine, Baltimore, MD, 21205, USA; e RTI International, Research Triangle Park, NC, 27709, USA; f Johns Hopkins Kavli Neuroscience Discovery Institute, Johns Hopkins University, Baltimore, MD, 21218, USA; g Department of Biostatistics, Johns Hopkins Bloomberg School of Public Health, Baltimore, MD, 21205, USA

**Keywords:** Prenatal nicotine exposure, Gestational smoking, Neurodevelopment, Bulk RNA-seq, Substance use disorders

## Abstract

Maternal smoking during pregnancy (MSDP) is associated with cognitive and behavioral effects on offspring. Although neurodevelopmental effects of prenatal nicotine exposure (PNE) have been described, the extent to which the molecular impacts of MSDP are attributable to nicotine remains unclear. In addition, the molecular responses to cigarette smoke in the developing and mature brain have not been contrasted. Distinguishing nicotine-specific effects from broader consequences of tobacco exposure is required to inform therapies for affected newborns and cessation strategies for pregnant women. In this study, pregnant mice received nicotine or were exposed to chronic cigarette smoke, and RNA sequencing was performed on frontal cortices of postnatal day 0 pups and adult dams. A total of 1010 and 4165 differentially expressed genes (DEGs) were identified in the nicotine and smoking-exposed pup brain, respectively (19 nicotine-exposed vs 23 vehicle-exposed; 42 smoking-exposed vs 46 controls). PNE was related to dopaminergic synapses and long-term synaptic depression and MSDP to functions of the SNARE complex and vesicle transport. No DEGs at FDR<0.05 were found in adult mouse brain for any exposure (11 nicotine-administered vs 7 vehicle-administered; 10 smoking-exposed vs 11 controls), nor in whole blood of adult dams–analyzed as proxy for brain effects (12 smoking-exposed vs 11 controls). Nonetheless, 29% and 43% of DEGs in smoking-exposed pup brain replicated in blood and human prenatal brain, respectively. Results demonstrate shared and unique effects of PNE and MSDP on the developing brain, attenuated impacts on adult brain, and convergent gene targets in human brain.

## Introduction

As of 2021, 4.6% of mothers in the United States smoked cigarettes during pregnancy. Although declining in prevalence over time, maternal smoking during pregnancy (MSDP) remains a major public health problem as it affects hundreds of thousands of mothers and their offspring [1,2]. Adverse health implications for pregnant women include increased risk for preterm deliveries and miscarriages, and impacts on lung and brain development from various toxic compounds in tobacco smoke for the unborn [3]. Prenatal tobacco exposure is also associated with cognitive and behavioral disruption. Specifically, exposed babies are predisposed to impaired language and learning skills, attention deficits, conduct and behavioral alterations, and are at higher risk of developing substance use disorders [4]. Several studies have investigated MSDP and prenatal nicotine exposure (PNE) in animal models and confirmed similar effects [4].

Because cigarette smoke contains a mixture of over 7000 compounds [5], understanding the molecular mechanisms by which tobacco smoke affects neurodevelopment is complex. Many of these constituents are toxic or carcinogenic and can disrupt brain function [6–10], and limited information is available regarding how individual components of cigarette smoke affect the developing brain during prenatal exposure. The most comprehensively studied substance is nicotine–the main psychoactive component of cigarette smoke. Nicotine activates and desensitizes nicotinic acetylcholine receptors in the developing central nervous system (CNS), and impacts brain development [4,11]. Despite extensive evidence demonstrating PNE affecting brain function [11], the extent to which nicotine accounts for MSDP effects remains unclear. The only transcriptomic investigation examining MSDP in the human prefrontal cortex identified 13 affected genes but did not distinguish nicotine-specific effects [12]. Nonetheless, identifying the molecular pathways driven by nicotine versus other components present in cigarette smoke is critical to target smoking-related neurodevelopmental damage. In addition, disentangling the effects of nicotine and other tobacco smoke constituents on pathways regulating addition and reward in the adult brain is warranted to refine our understanding of smoking behavior and develop targeted cessation strategies, particularly during pregnancy.

This study aimed to contrast nicotine-specific versus broader cigarette smoke molecular effects in the developing and adult mouse brain. MSDP and PNE were modeled under controlled conditions and their gene expression impacts were measured using bulk RNA sequencing in the frontal cortex of mouse offspring (P0: postnatal day 0), as well as on frontal cortex of the adult dams. Frontal cortex tissue was analyzed to establish neurodevelopmental exposure effects given the technical limitation the pup brain size imposes to obtain finer dissections. Additionally, whole blood was collected from the adult dams to interrogate the sharing of cigarette smoke transcriptomic effects in brain and blood, in order to investigate the suitability of blood-based toxicological brain studies. Differential expression analysis in pup brain identified genes affected by MSDP and PNE, distinguishing substance-specific and shared targets. PNE and MSDP molecular impacts were further characterized in expression features beyond gene expression, and variability in nicotine and smoking effects was assessed across ages (prenatal vs adult brain), tissues (brain vs blood), and species (human vs mouse brain).

## Materials and methods

Detailed materials and methods can be found in Supplementary Materials and Methods.

### Animals

Wild type male and female mice (C57BL/6 J; stock # 000,664, Jackson Laboratories) were purchased and used for timed breeding. 6 week old female mice were paired with male mice. Copulation plugs were checked daily and male mice were removed upon identification of plugs. Female mice were monitored for pregnancy, and separated upon pregnancy confirmation. Pups were euthanized by decapitation on the day of birth (postnatal day 0; P0). Pregnant dams were euthanized by decapitation and trunk blood was collected into a heparinized tube. Pup and adult brains were rapidly extracted from the skull and the frontal cortex was dissected from the brain over wet ice on a steel block using a scalpel. Frontal cortical tissue was snap frozen in chilled 2-methylbutane. Samples were transferred to tubes and placed on dry ice and stored at −80 °C until further processing for RNA extraction. In total, 208 samples were collected: 184 frontal cortex samples and 24 blood samples (Fig. 1A, Table S1). All experiments and procedures were approved by the Johns Hopkins Animal Care and Use Committee and in accordance with the Guide for the Care and Use of Laboratory Animals. No statistical method was used to determine the sample size and no randomization method was used to allocate animals to experimental groups, but balanced numbers of exposed and control animals were ensured across sample batches. Investigators were not blinded to nicotine administration or smoking exposure identities, and analysis of the data was not blinded.

### Nicotine administration

Free-base (−) nicotine (Cat no N3876, Sigma Aldrich) was dissolved in PBS (Cat no 10010023, ThermoFisher), and pH adjusted to 7.4. Nicotine was administered to the dams by s.c. injection (1.5mg/kg, ~0.1–0.2 mL) twice daily (8AM and 4PM), 5 days per week. This dosing regimen was used to produce systemic nicotine exposure, resembling intermittent exposure patterns experienced by smokers while minimizing excessive toxicity. Matched volumes of PBS were administered i. p. to control female dams. Administration started the week before mice were paired and continued until E17. This design was implemented to restrict the intervention to the prenatal period, avoiding effects associated with perinatal stress and postnatal exposure.

The non-pregnant cohort was exposed using the same dosing regimen as the pregnant dams and were exposed over a matched time window corresponding to the adult exposure condition.

### Smoking exposure

Dams were placed into a smoking chamber for 5 h/day, 5 days/week starting one week before mice were paired for breeding and until the time of delivery. The chamber contained a smoking machine (Model TE-10, Teague Enterprises, Davis, CA) that burns 5 cigarettes at a time (2R4F reference cigarettes (2.45 mg nicotine/cigarette; Tobacco Research Institute, University of Ky). The machine generated 2-second puffs at a flow rate of 1.05 L/min, producing a standard puff volume of 35 cm^3^ at a rate of 8 puffs per minute. Smoke was delivered as a mixture of 11% mainstream and 89% sidestream to mimic human smoking conditions. The chamber atmosphere was monitored continuously to maintain total suspended particulate at 90 mg/m^3^ and carbon monoxide at 350 ppm, providing a controlled exposure that approximates the smoke environment experienced by habitual smokers. Control dams were housed in matched exposure chambers operated under the same conditions, but supplied with filtered air instead of cigarette smoke, which they were exposed to for the same duration (5 h/day, 5 days/week). The non-pregnant cohort was exposed using the same cigarette smoke paradigm as the pregnant dams, over a corresponding adult time window.

The 5-day schedule for both nicotine administration and smoking exposure was designed to mimic a human chronic intermittent exposure pattern and avoid maternal stress induced by daily exposure.

### Tissue processing and RNA isolation and sequencing

Total RNA was extracted from samples using Trizol followed by purification with an RNeasy Micro kit (Qiagen). Paired-end strand-specific sequencing libraries were prepared and sequenced by Macrogen from 1ug total RNA using the TruSeq Stranded mRNA kit with ERCC Spike-in. Libraries were sequenced on an Illumina NovaSeq6000 S4, 150 bp paired end. Output was targeted at 60 M total reads (R1 30 M and R2 30 M, 150-bp paired-end reads).

### RNA-seq data processing and exploration

Raw sequencing reads were processed, aligned, and used to quantify expression of genes, transcripts, exons, and exon-exon junctions with *SPEAQeasy* [13] (Fig. 1B). Read counts were normalized by library size and composition, lowly expressed features excluded, and samples filtered according to quality control metrics (Fig. S1). Dimensionality reduction analysis identified drivers of expression variability across samples and detected additional poor-quality samples that were further excluded (Fig. S2–Fig. S5). Additional sources of gene expression variation were identified through variance partition analysis, which informed covariate selection in the models for differential expression analysis (DEA) (Fig. S6, Fig. S7).

### Differential expression analysis

Differential expression analysis was performed under the empirical Bayesian framework of *limma*-*voom* [14], comparing 1) nicotine vs vehicle exposure in pup brain, 2) smoking exposure vs control in pup brain, 3) nicotine vs vehicle administration in adult brain, 4) smoking exposure vs control in adult brain, and 5) smoking exposure vs control in adult blood (Fig. S8). Gene expression models were adjusted for batch effects, quality control metrics, sex (in pup brain), and pregnancy (in adult brain and blood). DEA of expression features other than genes were performed for smoking and nicotine exposures in pup brain. Only genes, transcripts, and exon-exon junctions with *p*-values adjusted for false discovery rate (FDR) <5%, as well as exons with adjusted *p* < 5% and |log_2_FC|>0.25, were called differentially expressed (DE).

Sex differences in smoking and nicotine effects were assessed by conducting DGE analysis in female and male pups separately, adjusting for same covariates. Sex-associated genes in the developing frontal cortex were identified by comparing transcriptomes of female and male pups, adjusting for exposure and same covariates.

Surrogate Variable Analysis (SVA) [15] estimated latent sources of gene expression variability in pup brain not explained by measured covariates. Differential expression analyses for PNE and MSDP at all four expression levels were repeated using the same *limma*-*voom* pipeline as in the primary analyses, additionally adjusting for estimated surrogate variables that captured potential unaccounted batch effects (Supplementary Materials and Methods: Surrogate Variable Analysis).

Spearman’s correlation was used to assess the similarity of exposure transcriptomic effects. The replication of DGE results across tissues and species was estimated based on the Storey π statistic [16] (Supplementary Materials and Methods: Differential expression analyses).

### Functional enrichment analysis

Genes annotated in Gene Ontology (GO) terms and in pathways of the Kyoto Encyclopedia of Genes and Genomes (KEGG) were assessed for over representation among sets of DEGs applying one-sided Fisher’s exact tests, as implemented in *clusterProfiler* [17], and were FDR controlled.

## Results

The frontal cortex was isolated from P0 offspring across two separate experiments: 1) pups born to dams exposed to gestational smoking (n = 46) or control dams (n = 49); 2) pups born to dams administered nicotine during gestation (n = 19) or dams administered vehicle during gestation (n = 23). The frontal cortex was also collected from the adult females that delivered the pups plus additional nonpregnant dams that were: 1) exposed to cigarette smoke (n = 12; 8 pregnant) or controls (n = 12; 7 pregnant), and 2) administered nicotine (n = 12; 3 pregnant) or vehicle-administered (n = 11; 3 pregnant). Additionally, blood samples were collected from all smoking-exposed and control adults (n = 24, Fig. 1A, Table S1). Total RNA was isolated from all 208 samples and bulk RNA sequencing was performed. From these data, the transcriptome was measured at four expression feature levels: genes, transcripts, exons, and exon-exon junctions (Fig. 1B). After quality control, this study analyzed 130 pup brain samples, 39 adult brain samples, and 23 blood samples (n = 192, Materials and Methods, Table S2).

### Molecular impact of gestational exposure to nicotine and smoking on developing frontal cortex of offspring

From the frontal cortex of P0 offspring from both the PNE and MSDP experiments, differential expression analysis was performed at the gene, transcript, exon, and exon-exon junction levels. Expression features other than genes were analyzed to support and complement gene-level results. Because gene-level expression is an aggregate of transcript-level expression [18–20], gene expression changes are often dominated by the highest expressed transcripts, obscuring subtler effects on low-abundance isoforms. Similarly, transcripts from the same gene that change in opposing directions can cancel each other out at the gene level, masking transcript-specific regulation [20–22]. Moreover, exons and exon-exon junction reads were analyzed to provide further insight into transcript abundance and potential alternative splicing events [23–26].

Comparing nicotine to vehicle exposure (PNE experiment; 19 nicotine-exposed vs 23 vehicle-exposed), 1010 differentially expressed genes (DEGs) were identified at FDR<0.05 (Fig. 2A, Table S3): 280 were downregulated and 730 upregulated. The top two most significantly up- and down-regulated genes were *Foxn3* and *Arrdc3*, and *Senp8* and *Coa4*, respectively (Fig. 2A). Comparing smoking exposure to control (MSDP experiment; 42 smoking-exposed vs 46 controls), 4165 genes were differentially expressed: 2106 were downregulated and 2059 upregulated (Fig. 2B, Table S4). *Top2a* and *Tpx2* were the most significant DEGs and were downregulated, followed by the upregulated *AB041806* and *Mt2* genes (Fig. 2B). While PNE and MSDP effects on gene expression were poorly correlated (*ρ*=0.13, *p*<8.2 × 10^–82^), 187 upregulated genes and 35 downregulated genes were shared across both (Fig. 2C). Among the shared upregulated DEGs, there were *Strap, Snrpd3*, and *Snrpb* that act in SMN-Sm protein complexes, and *Nsg1, Clstn1*, and *Rab4a* that participate in postsynaptic endosomes (Fig. S9A, Fig. S10A, B). Additionally, 496 genes were upregulated following nicotine exposure, but were not affected by cigarette smoke (Fig. 2C), of which 15 were associated with dopaminergic synapses and 8 with long-term synaptic depression (Fig. S9B, Fig. S10C,D). Similarly, 1855 genes were upregulated after smoking exposure, but not affected by nicotine, with 15 genes involved in the SNARE complex (Fig. S9A, Fig. S10E). Furthermore, 17 DEGs were upregulated by smoking exposure and downregulated by nicotine exposure; of these, *Stx17* and *Bnip1* were enriched for the SNARE complex (Fig. S9A–C, Fig. S10F). And 47 genes were upregulated by nicotine and downregulated by smoking, 4 showing enrichment for heat shock protein binding activity (Fig. S9C, Fig. S10G). A summary of the DGE results is provided in Table S5.

In order to examine sex differences in the transcriptomic effects of cigarette smoke and nicotine on the developing brain, DGE analyses comparing nicotine vs vehicle exposure, and smoking exposure vs control, were performed for female and male pups separately (11 nicotine- vs 11 vehicle-exposed females, 8 nicotine- vs 12 vehicle-exposed males, 22 smoking-exposed vs 22 control females, and 20 smoking-exposed vs 24 control males). While no genes at FDR<0.05 were identified for nicotine exposure within either sex, for smoking exposure 2686 and 886 genes passed the FDR<0.05 threshold in females and males, respectively (Fig. S11A). Sex-specific and shared effects were noted for both exposures, with moderate correlations in the gene expression signatures of both substances between sexes (*ρ*=0.21, *p*<2.7 × 10^–193^ for PNE and *ρ*=0.53, *p*<2.2 × 10^–16^ for MSDP, Fig. S11A). Notably, both sex-specific and shared effects were largely captured in the DGE results based on all pup samples analyzed together, adjusted for sex (Fig. S11B). Furthermore, DGE analysis comparing frontal cortex of female vs male pups (22 females vs 20 males in the PNE group, and 44 females vs 44 males in the MSDP group) revealed several sex-related genes that were significantly affected by PNE or MSDP (Fig. S11C). Together, these sensitivity analyses show that exposure effects are shared across sexes but several target genes may have sex-dependent implications in the pup brain.

Differential transcript expression (DTE) analysis identified 232 differentially expressed transcripts across 220 unique genes for PNE, and 4059 DE transcripts across 3451 unique genes for MSDP (Fig. S12, Table S6, Table S7). Comparing DTE against DGE results, PNE and MSDP effects were concordant at the gene and transcript levels (*ρ*=0.41, *p* < 2.2 × 10^–16^ for PNE, and *ρ*=0.50, *p* < 2.2 × 10^–16^ for MSDP, Fig. 3A, Table S8, Table S9), although several genes showed effects only at the transcript level and some presented oppositely regulated transcripts (Fig. S13). Functional profiles of differentially expressed transcripts corroborated and expanded DGE results: 1427 genes expressing upregulated transcripts under MSDP only, were enriched for the SNARE complex and transport vesicles (Fig. S9E, Fig. S10E,H), and 68 of them have been linked to Parkinson’s, Huntington’s, or prion-related diseases (Fig. S9F, Fig. S10I).

Differential exon expression (DEE) analysis identified 1115 and 5983 differentially expressed exons for PNE and MSDP, respectively (Table S10, Table S11), and exon- and gene-level effects were highly correlated (*ρ*=0.73, *p* < 2.2 × 10^–16^ for PNE and *ρ*=0.83, *p* < 2.2 × 10^–16^ for MSDP, Fig. 3B, Table S12, Table S13). In addition, analysis at the exon-exon junction level (DJE) revealed 205 and 9515 differentially expressed junctions for PNE and MSDP, respectively, including some previously unannotated that could be indicative of novel splicing events (Table S14, Table S15). Overall, agreement was found between results at all four expression levels, with 32% and 76.18% of the DEGs for PNE and MSDP affected in at least one additional feature level, respectively (Fig. 3C, Fig. S14–Fig. S16). DTE, DEE, and DJE results are further discussed (Supplementary File 1).

As a sensitivity analysis, the robustness of the results to unaccounted litter and batch effects was assessed by computing and adjusting expression models for latent sources of gene expression variation through surrogate variable analysis (SVA). Estimated PNE and MSDP transcriptomic effects at all four expression levels were largely correlated before and after adjustment for surrogate variables (*ρ*=0.85, *p* < 2.2 × 10^–16^ for PNE and *ρ*=0.72, *p* < 2.2 × 10^–16^ for MSDP gene-level effects, Fig. S17) and identified differentially expressed features substantially overlapped (99% and 52.6% of PNE DEGs, and 52.2% and 23.6% for MSDP DEGs, remained significant at *p* < 0.05 and FDR<0.05 after adjustment). Adjusted differential gene expression results for PNE and MSDP are provided in Table S16 and Table S17, respectively.

### Molecular impact of nicotine administration and smoking exposure on adult frontal cortex and blood

To ascertain whether identified molecular impacts of nicotine and cigarette smoke in pups are specific to the developing brain, PNE and MSDP effects were compared to those from nicotine vs vehicle administration, and smoking exposure vs control in the adult brain, respectively (Fig. 1A; 11 nicotine-administered vs 7 vehicle-administered, and 10 smoking-exposed vs 11 controls). Both substances impacted differently on gene expression in the adult brain (*ρ*=0.03, *p*<0.00017, Fig. 4A) but not significantly (Fig. S8A,B), and the effects of nicotine and cigarette smoke were variable between adult and pup brain (*ρ*=0.01, *p* < 0.055 for nicotine exposure and *ρ*=0.02, *p*<0.012 for smoking exposure, Fig. 4B,C, Table S5).

RNA was extracted from blood of smoking-exposed adult dams and controls to assess the replication of brain-level smoking effects in blood (Fig. 1A). Differential expression analysis comparing 12 smoking-exposed vs 11 control blood samples identified no DEGs at FDR<0.05 (Fig. S8C) and effects in blood and brain were uncorrelated (*ρ*=−0.01, *p*=0.17, Fig. S18A, Table S5). Nevertheless, according to the Storey π statistic (see Supplementary Materials and Methods: Differential expression analyses), 24% of nominally significant genes in adult brain for smoking exposure replicated in blood (*p* = 0.14). Similarly, 29% of MSDP-associated DEGs identified in pup brain replicated in adult blood at nominal significance (*p*=0.99, Fig. S18B). Cross-species comparison with human brain data further identified *Kcnn2*—previously reported as downregulated in prenatal human brain following smoking exposure (FDR<0.1) [12]—as also downregulated in smoking-exposed mouse blood (Fig. S18C, Table S18).

### Comparison of mouse transcriptomic changes with findings in human

In a previous study, the transcriptional impacts of prenatal and adult exposure to smoking on human prefrontal cortex were assessed using 33 prenatal and 207 adult, non-psychiatric postmortem brain samples. The smoking-exposed phenotype was defined by nicotine or cotinine detectability. MSDP was directly associated with differential expression of 13 genes (FDR<0.1; 16 smoking-exposed vs 17 unexposed prenatal tissue samples), whereas only 1 gene was found differentially expressed in adults (*MARCO* at FDR<0.1; 57 active smokers vs 150 non-smokers) [12]. The results from this study were used to assess the replication of differential gene expression from mouse to human, and vice versa.

Overall, transcriptomic smoking effects were largely uncorrelated in the brain of mouse and human for both prenatal (*ρ*=−0.06, *p* < 3.6 × 10^–12^, Fig. 5A) and adult exposure (*ρ*=−0.01, *p* = 0.29, Fig. 5B). Despite this, 43% of smoking-associated DEGs in pup brain replicated in the human prenatal brain with nominal significance (p = 0.87). Remarkably, *NRCAM* that encodes a cell adhesion protein involved in neuronal cell–cell interactions, and its mouse ortholog, were significantly down-regulated following prenatal smoking exposure (Fig. 5A). In contrast, replication was minimal in adults, with only 9 out of 525 nominally significant genes in the mouse brain replicating in the smoking-exposed human brain (1.71%, p = 0.91, Fig. 5B, Table S18).

Notably, *MARCO*, which encodes a pattern recognition receptor on immune cells, was significantly downregulated in the smoking-exposed adult human brain and the nicotine-exposed pup brain (Fig. 5C). In addition, the significantly upregulated genes *MPPED1* and *SDC1* in the smoking-exposed prenatal human brain replicated in the nicotine-exposed pup brain (Fig. 5D, Table S18).

## Discussion

### Transcriptomic changes induced by nicotine and smoking exposure in pup and adult frontal cortex

This study interrogated the transcriptomic effects of smoking and nicotine exposure in the developing and adult mouse brain. In the developing frontal cortex, a more widespread response to gestational smoking was identified compared to prenatal nicotine exposure, and both unique and shared transcriptional signatures were detected after PNE and MSDP.

Several of the most significantly affected genes by PNE and MSDP may help explain previously reported associations between these exposures and substance use disorders, as well as with neurodegenerative and neuropsychiatric conditions. In particular, PNE significantly upregulated *Foxn3* and *Arrdc3. Foxn3* has been associated with substance use and compulsive behaviors in human [27], raising the possibility that gestational nicotine exposure may contribute to addiction-related phenotypes later in life via dysregulation of this gene. However, the persistence and long-term consequences of such early-life dysregulation remain to be explored. In addition, *Arrdc3* has been proposed as a neuroprotective gene in Parkinson’s disease and acts as a regulator of locomotion [28,29] and may thus mediate PNE links to motor function [30,31]. Regarding downregulated genes by PNE, the most strongly affected were *Senp8* and *Coa4*, both implicated in neurodevelopment [32–34], and whose contribution to PNE neurodevelopmental consequences warrants further investigation.

Maternal smoking during pregnancy downregulated *Top2a*. This gene encodes the DNA topoisomerase II alpha that regulates pluripotency and differentiation of embryonic stem cells [35]. It has been demonstrated that maternal exposure to cigarette smoke components, such as metabolites of benzene, cause the transformation of the *Top2a* product into dangerous “molecular scissors” that fragment the genome in developing embryos, thereby increasing risk of neurodevelopmental conditions [36]. Indeed, it has been shown that the prenatal inhibition of *Top2a* causes autism-related behavioral defects in mice [37], consistent with epidemiological observations linking MSDP to increased autism risk in humans [38]. *Tpx2* was the second most downregulated gene by prenatal smoking and appears as a novel critical gene through which MSDP could elevate risk of neurodevelopmental disorders as it plays crucial functions in the division, positioning, and fate of neural stem cells in mice [39].

The most upregulated gene by MSDP was *Mt2*, a gene that encodes a metallothionein (MT) whose expression increases in response to neural injury and cellular stress [40–42]. Interestingly, MT genes have also been found upregulated in astrocytes of patients with Alzheimer’s and Parkinson’s diseases [40,43,44], conditions characterized by elevated cellular stress. These results are consistent with MSDP inducing cellular stress in the developing brain, and suggest that sustained or repeated activation of stress-response pathways could, in principle, contribute to long-term neural vulnerability [45–47]. In addition, MSDP upregulated transcripts of *Casp3, Psma6*, and *Nduvf1*, genes implicated in pathways underlying neurodegeneration. Together, these findings suggest that MSDP may be an understudied factor that could potentially contribute to neurodegenerative disorders in offspring through early perturbation of core pathogenic pathways, although evidence supporting this remains limited.

MSDP and PNE shared transcriptional signatures that indicate both, disruptions in regulatory mechanisms for brain development, and the activation of compensatory programs aimed at mitigating such perturbations. Genes upregulated after both exposures include *Strap, Snrpd3*, and *Snrpd*, critical components of the SMN-Sm protein complex. This complex is essential for pre-mRNA splicing, a process that ensures proper maturation and expression of transcripts during development [48]. The positive regulation of these genes may reflect compensatory mechanisms in the developing brain to preserve splicing events, suggesting that prenatal exposure to nicotine and smoking could perturb essential RNA-processing pathways crucial for neuronal differentiation and survival.

Additional shared upregulated DEGs were *Nsg1, Clstn1*, and *Rab4a*, all involved in the function of postsynaptic endosomes. These organelles are involved in trafficking and recycling of synaptic proteins that are essential for shaping developing neuronal connectivity [49]. The upregulation of these genes after PNE and MSDP may reflect an adaptive response to maintain intracellular trafficking, but could also result in imbalanced synaptic protein turnover, potentially altering synaptic plasticity and the formation of functional neuronal networks during critical periods of development.

Nicotine-specific dysregulated molecules were identified, which were enriched in dopaminergic synapses and synaptic long-term depression pathways. These results are consistent with the established role of nicotine in modulating dopamine signaling and synaptic plasticity [11,50–52]. Moreover, these findings indicate that nicotine alone can modulate neural pathways central to reward processing and learning during gestation, potentially contributing to increased vulnerability to addiction-related behaviors. The fact that these genes were not found significantly affected by cigarette smoke suggests that some transcriptomic effects of nicotine could be obscured or counteracted by the complex mixture of cigarette smoke constituents.

As expected, a substantially larger number of genes were affected by smoking and not nicotine alone. In total, 1855 genes were upregulated after MSDP, likely in response to non-nicotinic components of cigarette smoke, or due to interactions between nicotine and these additional constituents, and were enriched for the SNARE complex. This complex is made of a group of proteins that enable synaptic vesicles to fuse with the neuronal membrane, allowing neurotransmitter release into the synapse and thus being essential for neuronal communication and neural circuit development [53]. The upregulation of this core synaptic machinery following MSDP may therefore contribute to imbalanced circuit formation. More intriguingly, additional constituents of this complex were upregulated by MSDP but downregulated by PNE. This is further suggestive of the complex interplay between nicotine and other smoke components in regulating shared pathways, and emphasizes the relevance of accounting for such putative substance interactions when targeting smoking effects on neurodevelopment. Nevertheless, caution is warranted when interpreting contrasting effects of PNE and MSDP, as nicotine levels were not measured in this study and thus not compared across exposure paradigms. As such, any observed differences may be reflective of variable nicotine levels rather than substance composition.

Regarding sex differences, it is well known that sex influences the behavioral outcomes of prenatal tobacco and nicotine exposure [52,54]. This analysis identified a subset of genes exhibiting a response to PNE and MSDP that differs according to sex and may thus shed light on the mechanisms underlying such phenotypic differences. While detailed investigation of these effects was beyond the scope of this study, the dataset generated here provides a resource for future exploration.

Compared to the extensive differential expression signature in the developing brain, no significant transcriptomic alterations were observed in the mature brain. Similar results in smoking-exposed prenatal and adult human brains have been noted [12], which are consistent with a stronger response to early compared to adult exposure [55,56]. However, design aspects of this study may have also contributed to this observation. First, the adult cohort size (18 and 21 adults in the nicotine and smoking groups, respectively) has more limited statistical power to detect differential expression events compared to pup analyses. Second, all adult samples were derived from the frontal cortex of dams, precluding the assessment of sex-dependent responses and the identification of transcriptomic alterations in more specific, highly responsive regions such as the prefrontal cortex and nucleus accumbens [57,58]. Third, although nicotine administration and smoking exposure regimens were implemented to model moderate-to-heavy human smoking, exposure was limited to the experimental timeframe and may not fully recapitulate the cumulative effects of chronic human smoking. Future studies analyzing larger cohorts, both sexes, and additional brain regions will be necessary to characterize the long-term transcriptomic consequences of nicotine and smoking exposure in the adult brain.

### Gene expression changes induced by smoking in mouse brain and blood

Numerous epidemiological and toxicological studies have examined smoking-related molecular changes in blood as a proxy for effects in the brain. These approaches are generally motivated by the premise that smoking influences cardiovascular and brain health through shared biological mechanisms, such as inflammation and oxidative stress, or that brain perturbations may arise indirectly via vascular effects, including the circulation of pro-inflammatory mediators or ultrafine particulate matter capable of reaching the brain [59–61].

Concordant with previous reports, this study identified largely uncorrelated transcriptomic effects of cigarette smoke in adult brain and blood [62], suggesting that blood provides an imperfect proxy for molecular changes specific to the frontal cortex. Nonetheless, shared signatures were observed at nominal significance, consistent with systemic responses to smoking-induced cellular stress and inflammation. Among these, the downregulation of *Dusp14*, a negative regulator of MAPK and NF-κB signaling, is indicative of activated inflammatory pathways [63]. Reduced *Syt13* suggests disrupted cellular trafficking processes across tissues [64], and downregulated *Arhgef25*, a Rho guanine nucleotide exchange factor, points to impaired cytoskeletal remodeling that could affect axon and dendrite formation, as well as immune cell function [65–67].

In addition, the decreased expression of *KCNN2*, a calcium-activated potassium channel, in both prenatal human brain and mouse blood, suggests disruption of calcium-dependent signaling pathways with potential implications for neuronal excitability in the developing brain and immune function in the periphery. Consistent with its role in the CNS, mutations disrupting the function of *KCNN2* have been shown to cause neurodevelopmental movement disorders and locomotor deficits in both humans and rodents [68,69]. Notably, pharmacological inhibition of Kcnn2 channels in the nucleus accumbens has been shown to increase alcohol intake in mice, which further reduces channel expression [70]. Whether the downregulation of *KCNN2* following smoking contributes to reinforcement-related behaviors—and thus could be a target for addiction—warrants investigation. Importantly, exploring whether the observed prenatal downregulation of this gene in the human brain could underlie later addictive behaviors will be crucial to understand early molecular determinants of addiction risk.

### Shared smoking gene targets in mouse and human brain

Studying prenatal and adult smoking exposure in mice helps circumvent key confounders present in human studies, such as coexposure to other substances and poor prenatal care. However, important between-species differences—including gestational timing, dosing and exposure patterns, routes of administration, and pharmacokinetics—limit direct translatability to humans [4]. In fact, the present study found weak overall concordance in smoking gene expression effects between mouse and human brain, although shared disrupted genes were identified.

*NRCAM* and its mouse ortholog were consistently downregulated in the developing brain following cigarette smoke exposure [12]. This gene encodes a neuronal cell adhesion protein with essential roles in axon growth, guidance, and the formation of neural circuitry during brain development [71–74]. Consistent with its functional importance, *Nrcam*-null or deficient mice exhibit autism-related behavioral alterations and its dysregulation has been linked to psychiatric disorders and substance use [71–73]. Future studies are required that determine substance-specific effects of cigarette smoke on this gene and interrogate its potential behavioral and neuropsychiatric implications.

*MARCO* was downregulated in both the smoking-exposed human adult brain and the nicotine-exposed pup brain. The product of this gene is a scavenger receptor expressed in macrophages and microglia that mediates innate immune recognition and phagocytic clearance of pathogens and cellular debris. Prior studies in murine macrophage cell lines and human cell line-derived macrophages have shown that cigarette smoke reduces the expression of this gene, leading to impaired pathogen clearance [75,76]. The results from this study extend this evidence to the brain and suggest nicotine may be the one component of cigarette smoke driving such dysregulation, which can compromise brain immune function during development and adulthood [47,61].

Several limitations should be considered when interpreting the findings of this study, particularly in the pup brain. First, pups from the same litter are not independent, as they share genetic background and prenatal environment, reducing the effective sample size for estimating PNE and MSDP effects. Sensitivity analyses accounting for potential litter-related effects through surrogate variable adjustment yielded effect estimates that highly correlated with the primary results but identified fewer differentially expressed features at FDR<0.05. This supports the overall robustness of the estimated PNE and MSDP effects while warranting caution in the interpretation of individual features as significantly differentially expressed—particularly those with smaller effect sizes—as unmodeled litter dependence may have inflated statistical significance. Second, comparisons between MSDP and PNE effects may be confounded by between-experiment differences in housing conditions, as well as by inherent differences in the route of administration, exposure dynamics, timing, and levels of nicotine exposure across paradigms. Future studies incorporating nicotine and/or cotinine measurements will be necessary to establish exposure equivalence and enable more definitive comparisons across exposure models. Third, no functional assays or behavioral monitoring were conducted in this study, which aimed to identify target genes for hypothesis generation and potential future validation. Despite these limitations, this work identified known target genes of cigarette smoke in human with key neurological functions, as well as additional genes with experimentally supported or suggestive roles in neurodevelopmental and behavioral phenotypes.

In summary, this study identified nicotine-specific and broader cigarette smoke molecular alterations during mouse brain development. Gene-level findings were supported and extended by analyses at finer levels of expression, underscoring the complex, multi-level effects of smoking on the brain and highlighting candidates relevant to neurodevelopment, neurodegeneration, and addiction. Distinct effects of nicotine and cigarette smoke were observed in pup and adult brain, alongside limited concordance between blood and brain responses. Notably, this study replicated the effects of smoking on *KCNN2, NRCAM*, and *MARCO* in the human brain, meriting further investigation. By making all generated data publicly available through the *smokingMouse* [78] package, this work provides a foundation for future studies that may ultimately inform strategies to mitigate the pathological consequences of maternal smoking in both newborns and mothers.

## Supplementary Material

1

2

3

4

5

6

7

8

9

10

11

12

13

14

15

16

17

18

19

20

21

22

23

Supplementary material associated with this article can be found, in the online version, at doi:10.1016/j.addicn.2026.100270.

## Figures and Tables

**Fig. 1.**
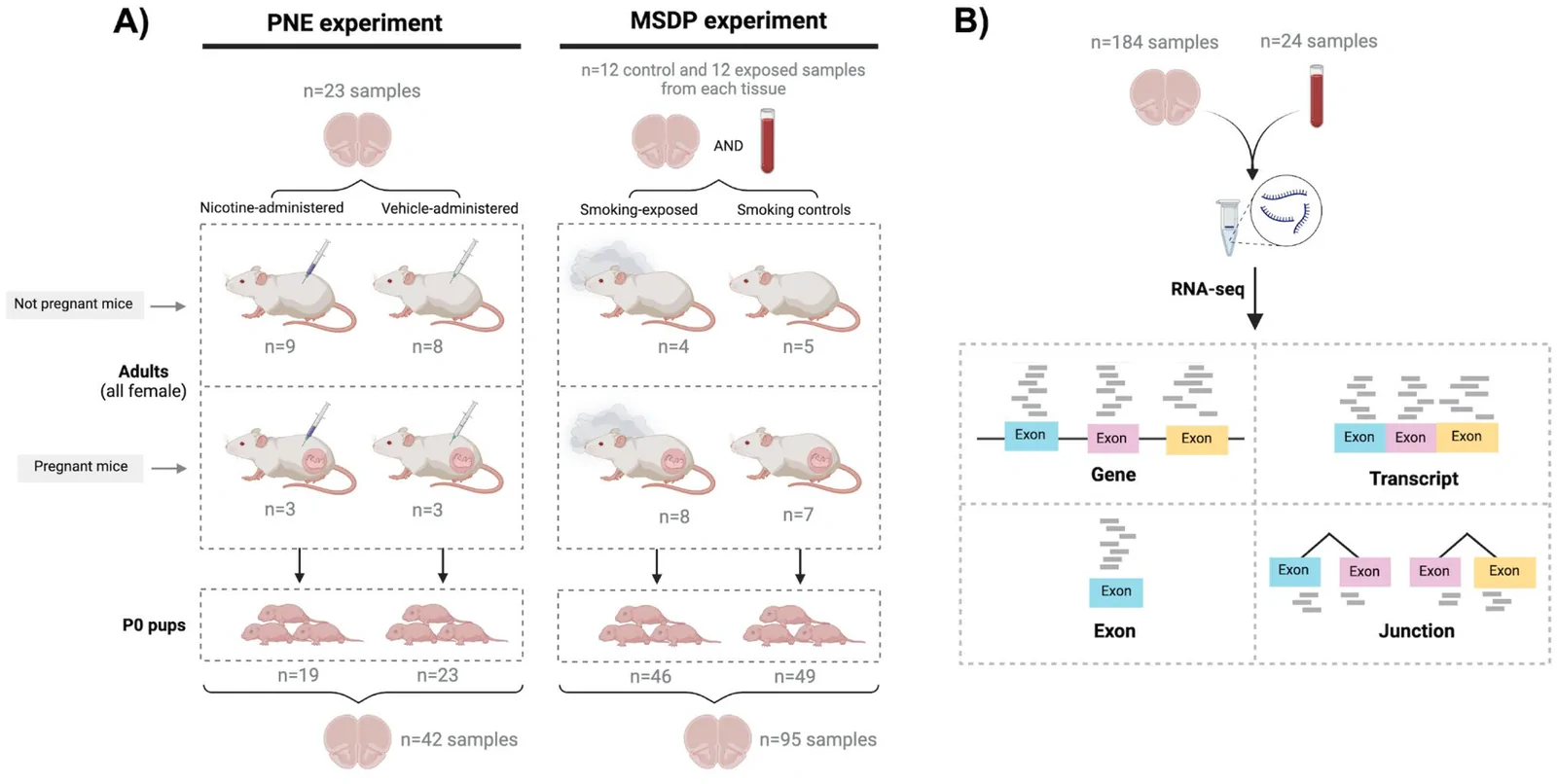
Study design. **A)** 21 pregnant mice were split into two experiments: in the first one prenatal nicotine exposure (PNE) was modeled administering nicotine (n = 3) or vehicle (n = 3) to the dams during gestation, and in the second maternal smoking during pregnancy (MSDP) was modeled exposing dams to cigarette smoke (n = 8) or filtered air (n = 7) during gestation. A total of 137 pups were born: 19 were born to nicotine-administered dams, 23 to vehicle-administered dams, 46 to smoking-exposed dams, and 49 to smoking control dams. 17 nonpregnant adult females were also nicotine-administered (n = 9) or vehicle-administered (n = 8) to model adult nicotine exposure, and 9 additional nonpregnant dams were smoking-exposed (n = 4) or controls (n = 5) to model adult smoking. Frontal cortex samples of all P0 pups (n = 137: 42 for PNE and 95 for MSDP) and adults (n = 47: 23 for the nicotine experiment and 24 for the smoking experiment) were obtained, as well as blood samples from the smoking-exposed and smoking control adults (n = 24), totaling 208 samples. **B)** RNA was extracted and bulk RNA-seq experiments performed, quantifying expression of genes, transcripts, exons, and exon-exon junctions.

**Fig. 2.**
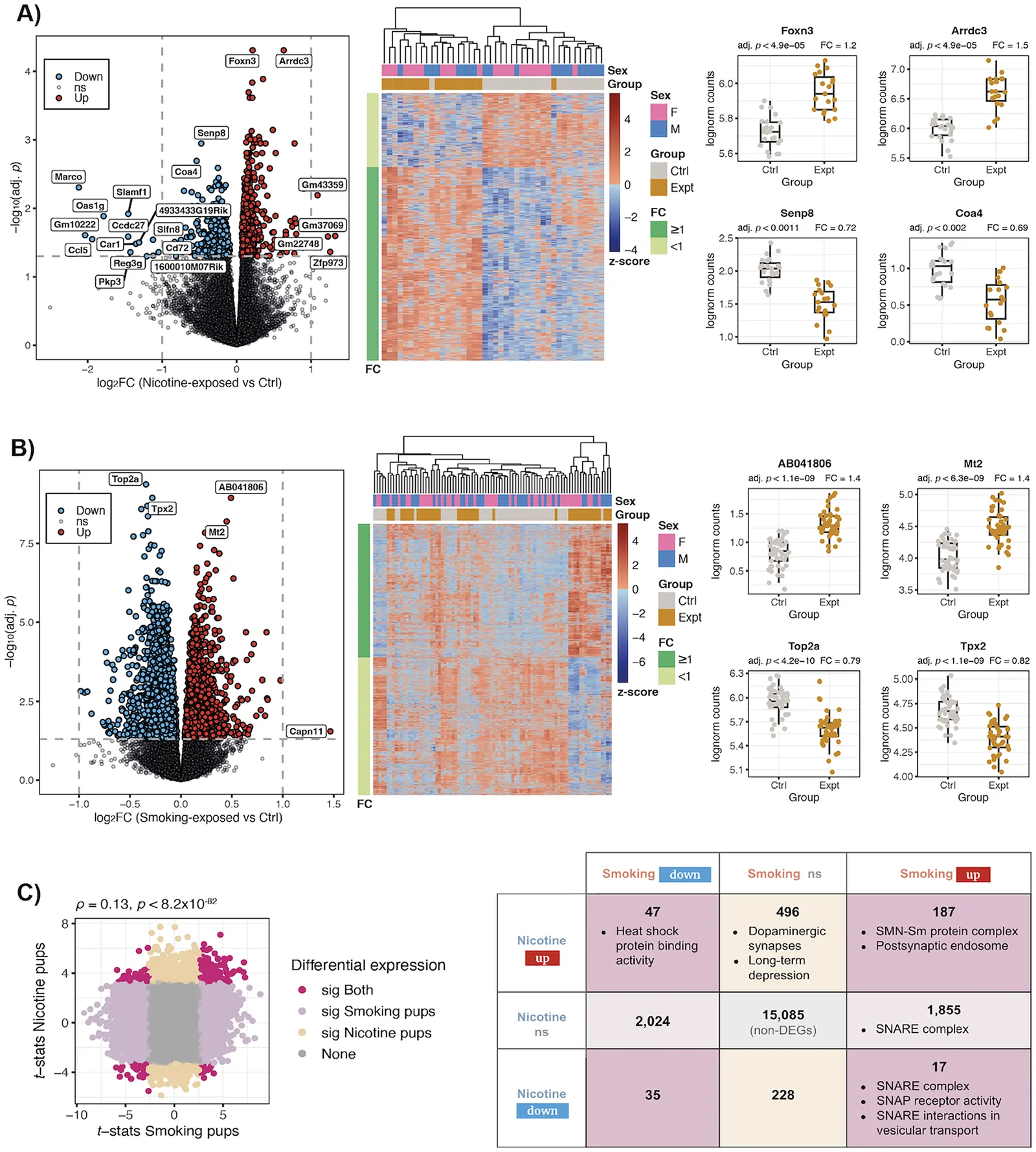
Differential gene expression following PNE and MSDP in pup brain. Results of differential gene expression analysis for **A)** prenatal nicotine vs vehicle exposure (PNE experiment) and **B)** prenatal smoking exposure vs control (MSDP experiment). Volcano plots show gene log_2_-fold-changes (log_2_FC) vs. FDR-adjusted *p*-values. Labeled genes had |log_2_FC|>1 or were the top 2 most significantly up- or down-regulated genes. Heat maps show the scaled log-normalized expression of DEGs across samples; left bars show gene FC direction. Box plots show the log_2_CPM of the top 2 most significant up- and down-regulated DEGs. **C)** Moderated *t*-statistics for gene expression following PNE and MSDP. In dark pink, genes significantly affected by both exposures; in light pink, genes significantly affected by smoking only; in beige, genes significantly affected by nicotine only, and in gray unaffected genes. Spearman’s correlation coefficient (*ρ*) and its *p*-value are shown. The right table shows the overlap between up- and down-regulated DEGs, as well as non-significant genes (n.s.), across PNE and MSDP. Molecular functions, cellular components, and pathways that were significantly enriched in the given sets of DEGs are indicated. Related to Fig. S9, Fig. S10, Table S3, Table S4 and Table S5.

**Fig. 3.**
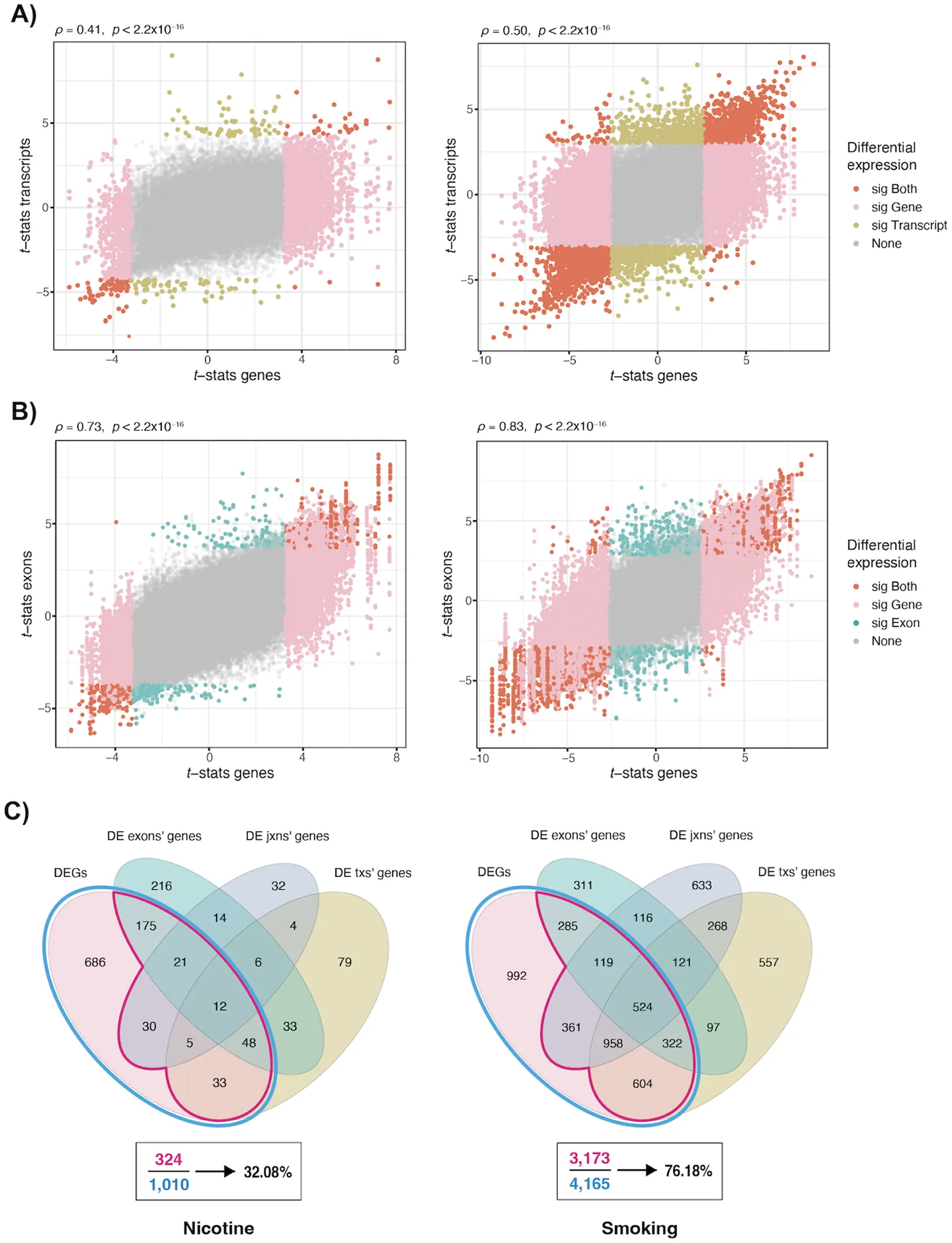
Differential expression at gene, transcript, exon, and exon-exon junction levels following PNE and MSDP in pup brain. Moderated *t*-statistics for differential expression of **A)** transcripts and **B)** exons vs. moderated *t*-statistics of their genes for PNE (left) and MSDP (right). In orange, differentially expressed (DE) features whose genes were also DE; in yellow and blue DE features of non-DEGs; in pink non-DE features of DEGs, and in gray, non-DE features of non-DEGs. Spearman’s correlation coefficient (*ρ*) and its *p*-value are shown. DE transcripts and genes were defined with an FDR<5% and DE exons with FDR<5% and | logFC|>0.25. **C)** Overlap between DEGs and genes expressing DE transcripts (txs), exons, and exon-exon junctions (jxns) in PNE and MSDP. The percentages of DEGs with any other DE feature are shown. Related to Fig. S13, Table S8, Table S9, Table S12, Table S13 and Supplementary File 1.

**Fig. 4.**
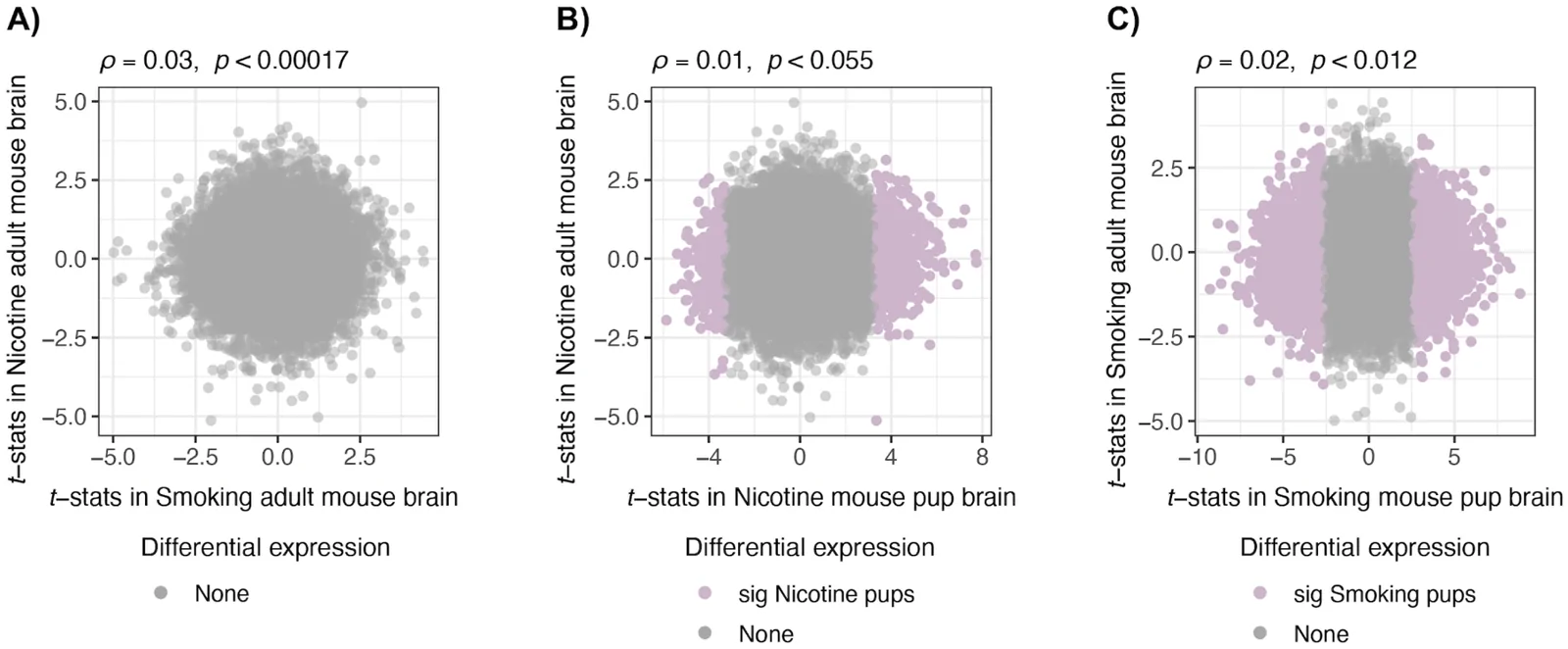
Differential gene expression following nicotine and smoking exposure in adult brain. Comparison of moderated *t*-statistics for **A)** nicotine vs smoking exposure in adult brain, and **B)** nicotine exposure and **C)** smoking exposure in adult vs pup brain. In pink DEGs in pup brain and in gray non-DEGs. Spearman’s correlation coefficient (*ρ*) and its *p*-value are shown. Related to Table S5.

**Fig. 5.**
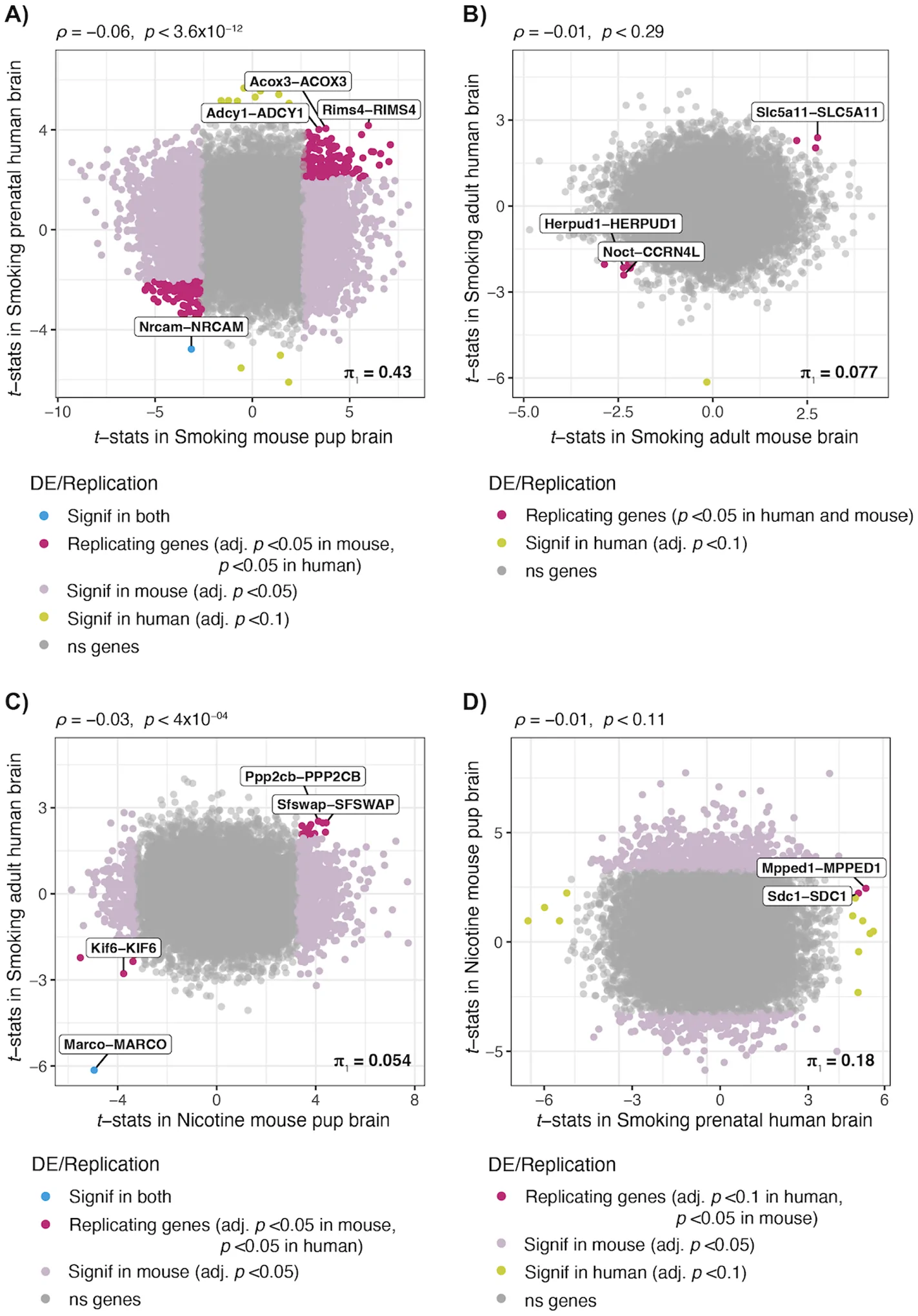
Differential gene expression following smoking exposure in mouse and human brain. Moderated *t*-statistics for (**A,B**) smoking exposure and (**C,D**) nicotine exposure in (**A,C,D**) pup and (**B**) adult mouse brain, compared against the moderated *t*-statistics for smoking exposure in (**A,D**) prenatal and (**B,C**) adult human brain. In dark pink, mouse (A-C) and human (D) brain genes that replicate in the other species (with *p*-value<0.05 and same logFC sign); in light pink, DEGs in pup brain (FDR<0.05); in yellow, DEGs in human brain (FDR<0.1); in blue, DEGs in both species, and in gray non-DEGs. Genes differentially expressed in both species, as well as the top 3 replicating genes most significant in human are labeled with their mouse and human gene names. Spearman’s correlation coefficient (*ρ*) and its *p*-value are shown, as well as the replication rate (*π*_1_). Related to Table S18.

## Data Availability

Unfiltered and normalized expression data generated and used in this project is available through the *smokingMouse* [78] Bioconductor data package, which also includes sample and feature-level data and the results from the DEA on human frontal cortex [12]. Raw bulk RNA-seq files are available from the NCBI Sequence Read Archive (BioProject PRJNA1175674).

## Code availability

Code for data analysis of this project can be found in the GitHub repository https://github.com/LieberInstitute/smoking-nicotine-mouse [77].

## Abbreviations

DE: differentially expressed or differential expression
DEA: differential expression analysis
DEE: differential exon expression
DEGs: differentially expressed genes
DGE: differential gene expression
DJE: differential exon-exon junction expression
DTE: differential transcript expression
FDR: false discovery rate
GO: Gene Ontology
GWAS: genome-wide association study
KEGG: Kyoto Encyclopedia of Genes and Genomes
MSDP: maternal smoking during pregnancy
P0: postnatal day 0
PNE: prenatal nicotine exposure
SNP: single nucleotide polymorphisms
TUD: tobacco use disorder
