# Supplementary material for: Molecular impact of nicotine and smoking exposure on the developing and adult mouse brain
Source: Addict Neurosci. Author manuscript; Available in PMC 2026 Oct 15. (PMC13662931; doi:10.1016/j.addicn.2026.100270)
Supplement: Supplementary file 1 [file NIHMS2211658-supplement-1.pdf]

SUPPLEMENTARY MATERIAL

Supplementary Materials and Methods

RNA-seq data processing

Read alignment and expression quantification

Quality assessment of sequencing reads and expression quantification at gene, exon, transcript, and exon-exon junction levels were performed running the RNA-seq processing pipeline *SPEAQeasy* version 6c1dab0 (13), using default settings, which involved alignment of reads to the Mus musculus genome GENCODE M25 (79,80). As part of *SPEAQeasy, featureCounts* (18) was used for gene and exon quantification, using the `-O` argument for exon features in order to assign reads to all the overlapping exons. This setting has the drawback that it can inflate exon read counts. *RegTools* (81) was used for junction quantification and *Kallisto* (82) performed the pseudoalignment of the reads to the transcriptome.

Count normalization

Raw counts were normalized by sample library size calculating normalization factors with `calcNormFactors()` from *edgeR* v3.43.7 (83), using the Trimmed Mean of M-values (TMM) method (84) for genes and exons, and the TMM with singleton pairing (TMMwsp) method for junctions. After rescaling library sizes, counts were transformed into counts per million (CPM) in a logarithmic scale ($\log_2$(CPM + 0.5)). For transcripts, transcripts per million (TPM) were transformed to $\log_2$(TPM + 0.5).

Feature filtering

Lowly expressed genes, exons, and exon-exon junctions were filtered with `filterByExpr()` from *edgeR* v3.43.7 (83), which retains features that have a minimum of 15 reads across all samples and at least 10 reads in *n* or more samples, where *n* is 70% the size of the smallest sample group. Transcripts were filtered by defining a mean TPM expression cutoff of 0.28 according to `expression_cutoff()` from *jaffelab* v0.99.32 (85).

RNA-seq data analysis

Exploratory data analysis

Quality control (QC) metrics of samples were direct outputs of *SPEAQeasy* (13) and additional ones were computed with `addPerCellQC()` from *scuttle* v1.9.4 (86). These metrics were calculated from raw counts of genes before normalization and filtering and were examined separately for adult and pup brain samples, and adult blood samples. Samples with low outlier values for library size or number of detected genes, or high outlier values for the percentage of

mitochondrial or ribosomal read counts, were excluded (**Fig. S1**).

Principal Component Analysis (PCA) at gene, transcript, and exon levels exhibited additional poor-quality samples that were further excluded from analysis (**Fig. S2**, **Fig. S3**). Multidimensional scaling (MDS) analysis supported PCA results among filtered adult and pup brain samples (**Fig. S4**, **Fig. S5**).

To inform covariate selection for gene expression modeling, the percentage of variance explained by each variable was computed for each gene using `getVarianceExplained()` from *scater* v1.29.1 (86) (**Fig. S6**). In addition, the fraction of variance explained (FVE) by each variable while accounting for all other covariates in the model was estimated using `fitExtractVarPartModel()` from *variancePartition* v1.32.2 (87) (**Fig. S7**). To account for potential confounding and avoid redundancy among covariates, canonical correlation analysis (CCA) was performed using the `canCorPairs()` function from *variancePartition*. Variables highly correlated with the exposure of interest were included, and for pairs of highly correlated covariates, only the variable with the highest median fraction of variance explained (FVE) was included in the final model (**Fig. S7**).

Differential expression analyses

Differentially expressed features for nicotine vs vehicle exposure, and smoking exposure vs control were identified in pup brain, and adult brain and blood by defining the following models:

- For smoking exposure vs control in adult blood, and both nicotine vs vehicle administration and smoking exposure vs control in adult brain (analysis only at the gene level):
$$y_{ij} = \beta_{0i} + \beta_{1i}Group_j + \beta_{2i}Pregnancy_j + \beta_{3i}plate_j + \beta_{4i}flowcell_j + \beta_{5i}rRNA\_rate_j + \beta_{6i}totalAssignedGene_j + \beta_{7i}ERCCsumLogErr_j + \beta_{8i}overallMapRate_j + \varepsilon_{ij}$$
- For nicotine vs vehicle exposure, and smoking exposure vs control in pup brain (analysis at the four levels of expression features):
$$y_{ij} = \beta_{0i} + \beta_{1i}Group_j + \beta_{2i}Sex + \beta_{3i}plate_j + \beta_{4i}flowcell_j + \beta_{5i}rRNA\_rate_j + \beta_{6i}totalAssignedGene_j + \beta_{7i}ERCCsumLogErr_j + \beta_{8i}overallMapRate_j + \beta_{9i}mitoRate_j + \varepsilon_{ij}$$

Where y_{ij} denotes the expression of the ith feature in the jth sample, modeled by selected covariates (see **Table S19** for their description).

DGE analyses comparing nicotine vs vehicle exposure, and smoking exposure vs control in pup brain were performed for females and males separately, fitting the same models used for the whole sample (excluding Sex). DGE analysis comparing frontal cortex of female vs male pups was performed in PNE and MSDP samples separately, using the same models adjusted for nicotine/smoking exposure.

Differential expression analysis was performed using the *limma-voom* pipeline (limma v3.57.6) (14). For gene, transcript, and exon counts, `voom()` was applied to transform normalized counts to log-counts per million (log-CPM) and to estimate observational-level inverse variance weights based on the mean–variance relationship, thereby accounting for heteroscedasticity in count data (**Fig. S8**). The resulting log-CPM (or log-TPM) values and associated weights were then analyzed using `lmFit()` to fit linear models for each feature via weighted least squares. Subsequently, `eBayes()` was applied to perform empirical Bayes moderation of the feature residual variances, improving the stability of variance estimates and reliability of differential expression inference. *P*-values of moderated *t*-statistics were adjusted for multiple testing with Benjamini and Hochberg's method (88) (**Fig. S8**). Only genes, transcripts, and exon-exon junctions with FDR<0.05, as well as exons with FDR<0.05 and $|\log_2 FC|>0.25$, were considered differentially expressed.

The monotonic correlation between transcriptomic exposure effects (i.e. the moderated *t*-statistics for nicotine/smoking exposure) across experiments, sexes, expression features, ages, tissues, and species was assessed using Spearman's correlation coefficient (ϱ) along its corresponding *p*-value (89).

Replication of mouse brain differential gene expression results in blood and human brain was defined by FDR<0.05 in pup brain or *p*-value<0.05 in adult brain, *p*-value<0.05 in blood or human brain, and concordant effect directionality. Replication of human brain differential gene expression in mouse blood or brain was defined by FDR<0.1 in human, *p*-value<0.05 in mouse, and concordant effect directionality. Note that when comparing mice and human, analyses were performed on gene pairs (mouse-human orthologs) rather than individual genes. The significance of the number of replicating genes was evaluated using one-sided Fisher's exact test.

The rate of replication of mouse or human brain DEGs (or nominally significant genes) in mouse blood and in the brain of the other species, was calculated as $\hat{\pi}_1 = 1 - \hat{\pi}_0$, where $\hat{\pi}_0$ is the estimated Storey π statistic (16), representing the proportion of such brain DEGs (or nominally significant genes) expected to be true nulls in the other tissue or species. π_0 was estimated using `qvalue()` of *qvalue* v2.33.0 (90), following the approach of Hoffman et al. (91).

Surrogate variable analysis

Surrogate variable analysis (SVA) was implemented to estimate latent sources of variation in pup brain gene expression related to unaccounted litter and batch effects. Following the two-step procedure of Leek et al. 2007 (15), the effects of modeled variables were first regressed out from the log-normalized gene expression matrices for both PNE and MSDP analyses. Singular value decomposition was applied to the residual matrices to identify orthogonal residual eigengenes representing independent sources of variability not explained by adjusted covariates. Residual eigengenes significantly explaining more variation than expected by chance were identified through the permutation procedure of Buja and Eyuboglu, 1992 (92).

For each significant residual eigengene, signature genes were identified based on the association of their log-normalized expression, and singular value decomposition was applied to the subset of the log-normalized expression matrix for such genes. The resulting singular vector most highly correlated with the original residual eigengene was used as surrogate variable (SV). By estimating SVs from the full normalized expression of signature genes, these were allowed to represent additional sources of variability that might overlap with accounted covariates. These analyses were implemented through `twostepsva.build()` of *sva* v3.58.0 (93).

Differential expression analysis at the gene, transcript, exon, and exon-exon junction levels for PNE and MSDP in the pup brain were conducted as previously described but including estimated SVs in the models. Results were compared to those from the unadjusted models at $p<0.05$ and FDR<0.05 (**Fig. S17**).

Differential gene expression visualization

Z-scores from log-normalized counts of DEGs were visualized in heat maps, using complete-linkage hierarchical clustering and euclidean distance.

Novel exon-exon junction gene annotation

The nearest (overlapping) neighbor and closest downstream and upstream (non-overlapping) genes of DE novel junctions were found using the functions `nearest()`, `precede()` and `follow()`, respectively, from *IRanges* v2.36.0 (94).

Software

ggplot2 v3.4.4 (95), *R* version 4.3.0 (89), and *Bioconductor* version 3.18 (96) were used to perform all the analyses and visualize the results.

Supplementary Tables

Supplementary Table 1: Study design. Number of samples from each pair of sample-level variables. See **Table S19** for sample variable description.

Supplementary Table 2: Analyzed samples. Number of samples from each pair of sample-level variables after sample filtering based on QC metrics and PCA plots. See **Table S19** for sample variable description.

Supplementary Table 3: DEGs in the nicotine-exposed pup brain. Metadata of DEGs in the nicotine pup brain and their logFC, moderated *t*-stats, *p*-value, and adjusted *p*-value for DE. The statistics were computed with `topTable()` from *limma*; see its documentation for the definition of the variable names. Related to **Fig. 2A**.

Supplementary Table 4: DEGs in the smoking-exposed pup brain. Same as in **Table S3** but for DEGs in the smoking pup brain. Related to **Fig. 2B**.

Supplementary Table 5: Differential gene expression results for the complete gene dataset. Gene-level metadata and the logFC, moderated *t*-stats, *p*-value, and adjusted *p*-value for DE of each gene across the 5 experimental groups: nicotine vs vehicle exposure in pup brain, smoking exposure vs control in pup brain, nicotine vs vehicle administration in adult brain, smoking exposure vs control in adult brain, and smoking exposure vs control in adult blood. Also included are the replication results of the genes in mouse blood. The statistics were computed with `topTable()` from *limma*; see its documentation for the definition of the variable names. Related to **Fig. 2C, Fig. 4** and **Fig. S18A-B**.

Supplementary Table 6: Differentially expressed transcripts in the nicotine-exposed pup brain. Metadata of DE transcripts in the nicotine pup brain and their logFC, moderated *t*-stats, *p*-value, and adjusted *p*-value. The statistics were computed with `topTable()` from *limma*; see its documentation for the definition of the variable names.

Supplementary Table 7: Differentially expressed transcripts in the smoking-exposed pup brain. Same as in **Table S6** but for DE transcripts in the smoking pup brain.

Supplementary Table 8: Differential expression of transcripts vs genes for nicotine exposure in pup brain. DE statistics (logFC, moderated *t*-stats, *p*-value, and adjusted *p*-value) for transcripts and their respective genes for nicotine exposure in pup brain, and whether transcripts and genes were both DE. Only transcripts of genes present in the gene dataset are shown. The statistics were computed with `topTable()` from *limma*; see its documentation for the definition of the variable names. Related to **Fig. 3A**.

Supplementary Table 9: Differential expression of transcripts vs genes for smoking exposure in pup brain. Same as in **Table S8** but for the smoking exposure. Related to **Fig. 3A**.

Supplementary Table 10: Differentially expressed exons in the nicotine-exposed pup brain. Metadata of DE exons in the nicotine pup brain and their logFC, moderated *t*-stats, *p*-value, and adjusted *p*-value. The statistics were computed with `topTable()` from *limma*; see its documentation for the definition of the variable names.

Supplementary Table 11: Differentially expressed exons in the smoking-exposed pup brain. Same as in **Table S10** but for DE exons in the smoking pup brain.

Supplementary Table 12: Differential expression of exons vs genes for nicotine exposure in pup brain. DE statistics (logFC, moderated *t*-stats, *p*-value, and adjusted *p*-value) for exons and their respective genes for nicotine exposure in pup brain, and whether exons and genes were both DE. Only exons of genes present in the gene dataset are shown. The statistics were computed with `topTable()` from *limma*; see its documentation for the definition of the variable names. Related to **Fig. 3B**.

Supplementary Table 13: Differential expression of exons vs genes for smoking exposure in pup brain. Same as in **Table S12** but for the smoking exposure. Related to **Fig. 3B**.

Supplementary Table 14: Differentially expressed exon-exon junctions in the nicotine-exposed pup brain. Metadata of DE exon-exon junctions in the nicotine pup brain, including for each:

- whether both the donor and acceptor sites together are known and annotated in GENCODE M25 (`inGencode` variable);
- whether the donor and acceptor sites are individually annotated in GENCODE M25 (`inGencodeStart` and `inGencodeEnd` variables, respectively);
- junction class: *Novel* (if both start and end sites are unknown, also known as fully novel junctions), *InGen* (already annotated in GENCODE M25), *AltStartEnd* (if it has only one known site), or *ExonSkip* (with sites from non-successive exons, both known individually but not together), and
- whether they are fusion junctions, meaning that they connect exons from different genes (`isFusion` variable).

Their logFC, moderated *t*-stats, *p*-value, and adjusted *p*-value are provided. These statistics were computed with `topTable()` from *limma*; see its documentation for the definition of the variable names.

Supplementary Table 15: Differentially expressed exon-exon junctions in the smoking-exposed pup brain. Same as in **Table S14** but for DE exon-exon junctions in the smoking pup brain.

Supplementary Table 16: Differential gene expression results for nicotine exposure after SV adjustment. Gene-level metadata and the logFC, moderated *t*-stats, *p*-value, and adjusted *p*-value for DE of each gene comparing nicotine vs vehicle exposure in the pup brain, after adjusting DGE models for batch-related surrogate variables. The statistics were computed with `topTable()` from *limma*; see its documentation for the definition of the variable names. Related to **Fig. S17**.

Supplementary Table 17: Differential gene expression results for smoking exposure after SV adjustment. Same as in **Table S16** but for the smoking exposure. Related to **Fig. S17**.

Supplementary Table 18: Differential gene expression results for gene pairs of mouse-human orthologs. For human-mouse ortholog pairs, their logFC, moderated *t*-stats, *p*-value, and adjusted *p*-value for nicotine and smoking exposure in prenatal and adult human brain, pup and adult mouse brain, and mouse blood, are presented. Only mouse genes with human ortholog(s) present in the human dataset from (12) are included. The DE statistics were computed with `topTable()` from *limma*; see its documentation for the definition of the variable names. Related to **Fig. 5** and **Fig. S18C**.

Supplementary Table 19: Dictionary of sample variables. Description of sample variables used throughout this project.

Supplementary Table 20: Associated genes of fully novel DE exon-exon junctions in pup brain. Nearest, following, and preceding genes of fully novel DE exon-exon junctions without assigned gene for

the nicotine and smoking exposure in pup brain.

Supplementary Figures

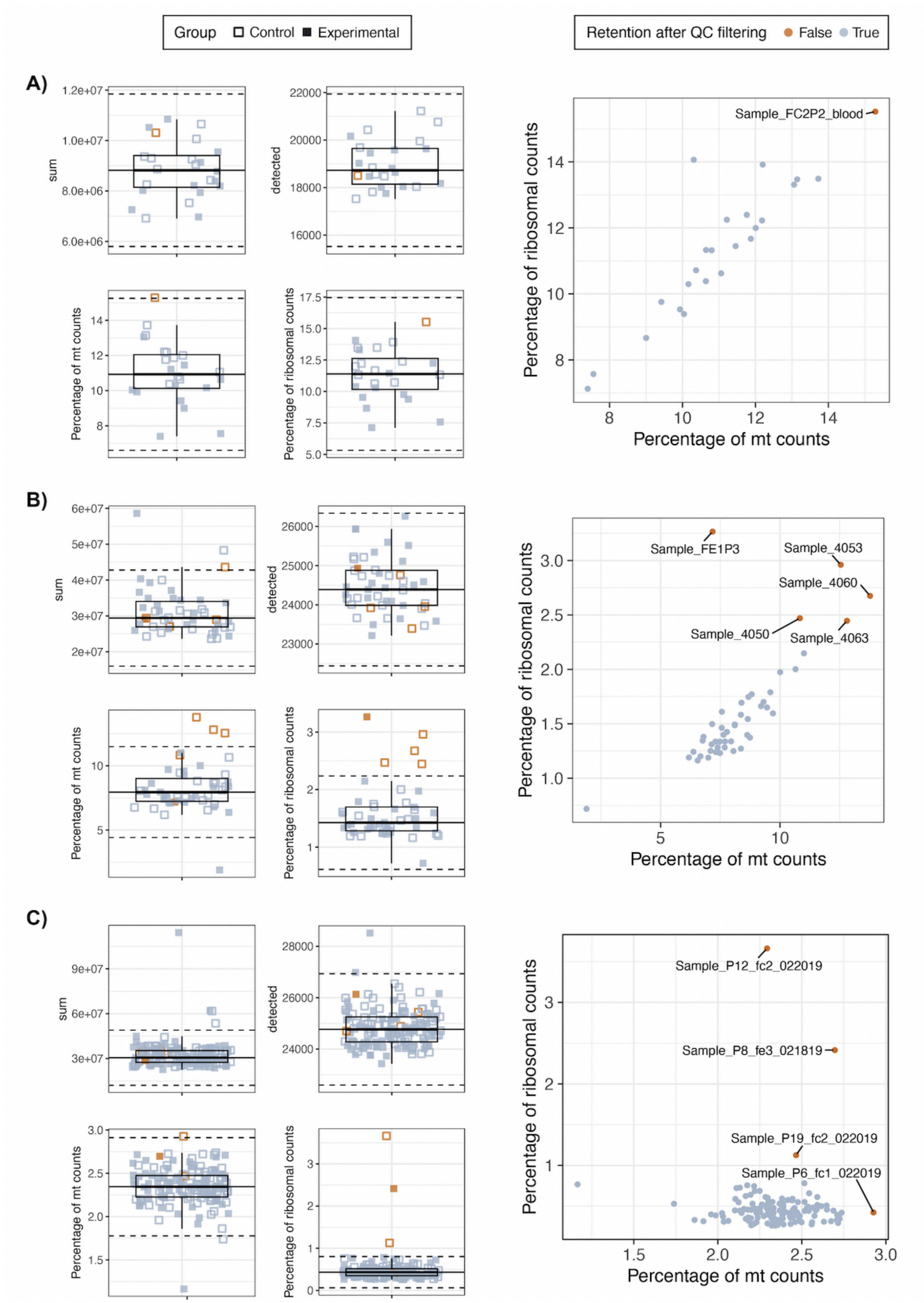

Group
Control
Experimental
Retention after QC filtering
False
True
A)
sum
detected
Percentage of mt counts
Percentage of ribosomal counts
Sample_FC2P2_blood
B)
Sample_FE1P3
Sample_4053
Sample_4060
Sample_4050
Sample_4063
C)
Sample_P12_fc2_022019
Sample_P8_fe3_021819
Sample_P19_fc2_022019
Sample_P6_fc1_022019

Supplementary Figure 1: Sample filtering by QC metrics. Box plots show the library size (sum), the number of detected genes (detected), the percentage of total sample reads mapping to mitochondrial (mt) and ribosomal genes in **A)** adult blood, **B)** adult brain, and **C)** pup brain samples. Dotted lines are 3 median-absolute-deviations away from the median (solid line) and set the cutoff values to detect outliers; low outlier samples in library size or detected number of genes, and high outlier samples in mitochondrial or ribosomal percentages were considered poor-quality and were excluded (in orange). Scatter plots on the right show the same percentages of mitochondrial and ribosomal counts, labeling excluded samples.

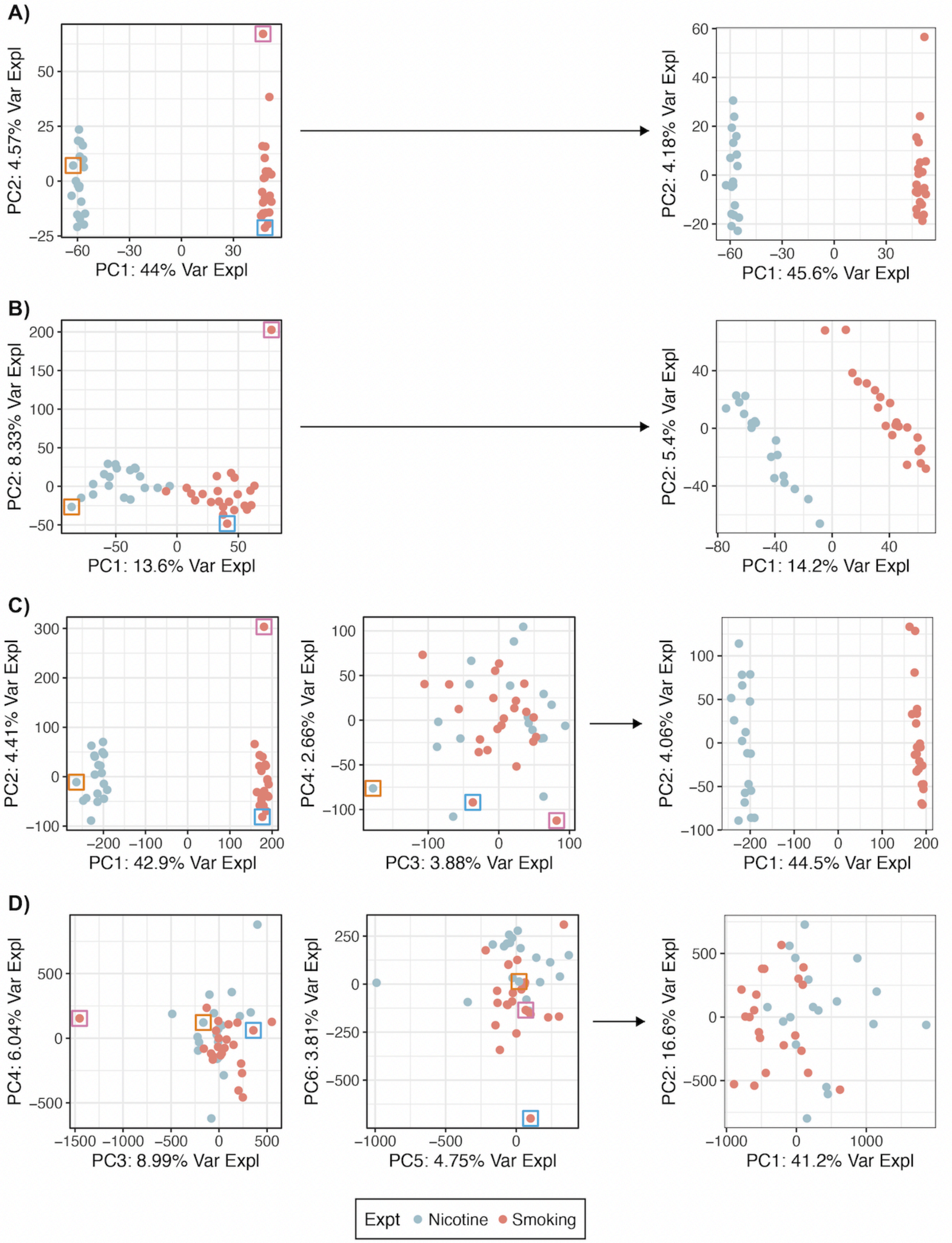
A)
PC1: 44% Var Expl
PC2: 4.57% Var Expl
PC1: 45.6% Var Expl
PC2: 4.18% Var Expl
B)
PC1: 13.6% Var Expl
PC2: 8.33% Var Expl
PC1: 14.2% Var Expl
PC2: 5.4% Var Expl
C)
PC1: 42.9% Var Expl
PC2: 4.41% Var Expl
PC3: 3.88% Var Expl
PC4: 2.66% Var Expl
PC1: 44.5% Var Expl
PC2: 4.06% Var Expl
D)
PC3: 8.99% Var Expl
PC4: 6.04% Var Expl
PC5: 4.75% Var Expl
PC6: 3.81% Var Expl
PC1: 41.2% Var Expl
PC2: 16.6% Var Expl
Expt Nicotine Smoking

Supplementary Figure 2: Manual filtering of adult brain samples. Principal Components (PCs) for **A)** gene, **B)** transcript, **C)** exon, and **D)** exon-exon junction expression in brain samples from adult mice. Samples are colored by nicotine and smoking exposure experiment. Left and middle plots contain all samples that passed QC metric-based sample filtering (see **Fig. S1**). Right plots resulted from removing samples that appeared isolated in PC plots, boxed in different colors. Dots boxed in the same color correspond to the same sample. PCs shown were chosen based on where effects were clearly visible and outlying samples evident. Note the cleaner sample separation by experiment after excluding these samples.

- Pink-boxed sample: sample with the highest proportion of rRNA counts.
- Orange-boxed sample: sample with the highest proportion of reads that mapped to the mitochondrial chromosome, highest percentage of mitochondrial and ribosomal counts, lowest proportion of reads assigned to genes, and the smallest number of detected genes.
- Blue-boxed sample: sample with the lowest fraction of reads which successfully mapped to the reference genome and the smallest library size.

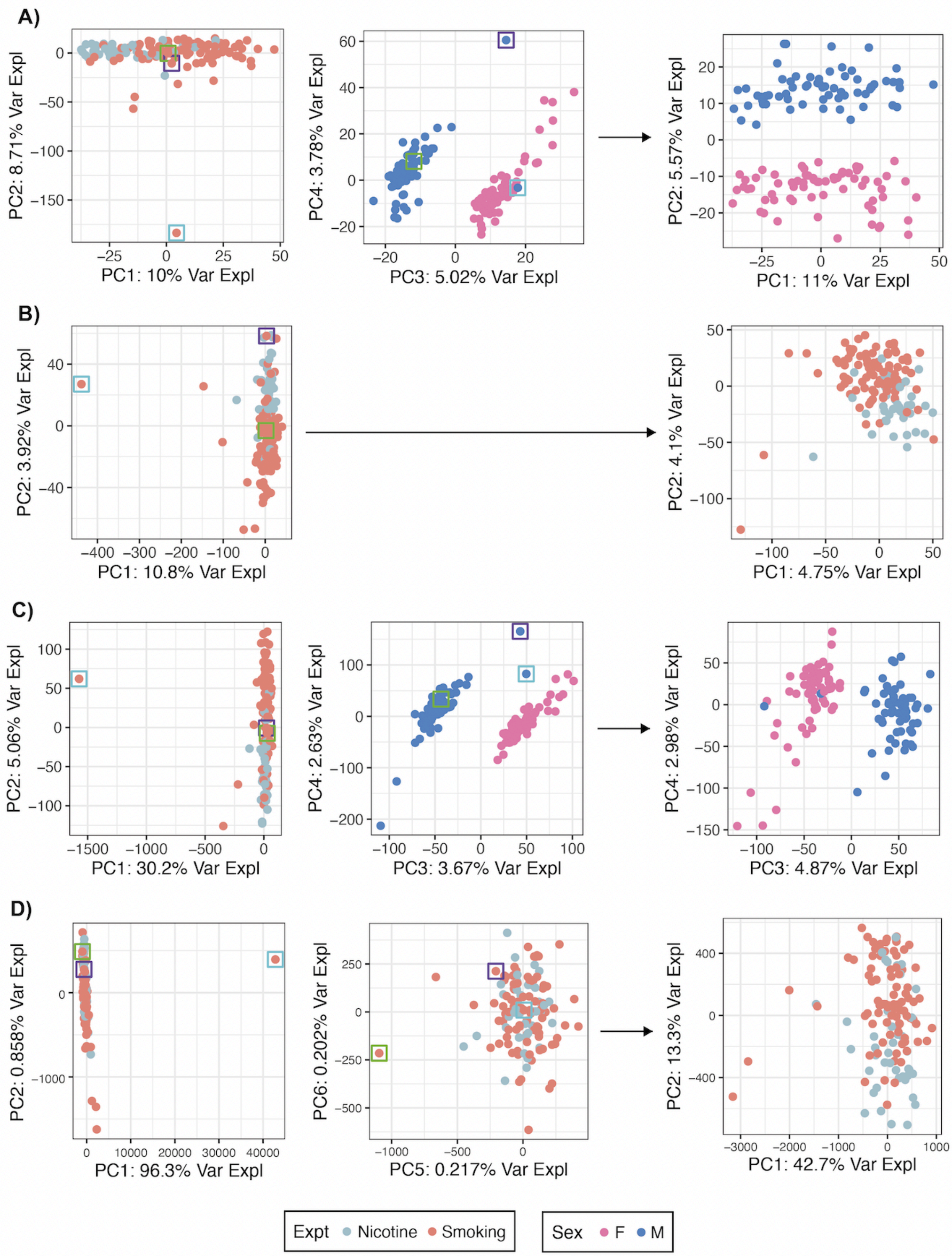
A)
B)
C)
D)
PC1: 10% Var Expl
PC2: 8.71% Var Expl
PC3: 5.02% Var Expl
PC4: 3.78% Var Expl
PC1: 11% Var Expl
PC2: 5.57% Var Expl
PC1: 10.8% Var Expl
PC2: 3.92% Var Expl
PC1: 4.75% Var Expl
PC2: 4.1% Var Expl
PC1: 30.2% Var Expl
PC2: 5.06% Var Expl
PC3: 3.67% Var Expl
PC4: 2.63% Var Expl
PC3: 4.87% Var Expl
PC4: 2.98% Var Expl
PC1: 96.3% Var Expl
PC2: 0.858% Var Expl
PC5: 0.217% Var Expl
PC6: 0.202% Var Expl
PC1: 42.7% Var Expl
PC2: 13.3% Var Expl
Expt Nicotine Smoking
Sex F M

Supplementary Figure 3: Manual filtering of pup brain samples. Principal components for **A)** gene, **B)** transcript, **C)** exon, and **D)** exon-exon junction expression in brain samples from pups. Left and middle plots contain all samples that passed QC metric-based filtering (see **Fig. S1**). Right plots resulted from removing samples that appear segregated or atypical in PC plots, which are boxed in different colors. Dots boxed in the same color correspond to the same sample. PCs shown were chosen based on where effects were clearly visible and outlying samples evident. Note the cleaner sample separation by sex after excluding these samples.

- Blue-boxed sample: sample with the lowest proportion of reads assigned to genes.
- Purple-boxed sample: sample with the highest overall difference between expected and observed External Control Consortium (ERCC) RNA concentrations.
- Green-boxed sample: sample with the lowest fraction of reads which successfully mapped to the reference genome and the smallest library size.

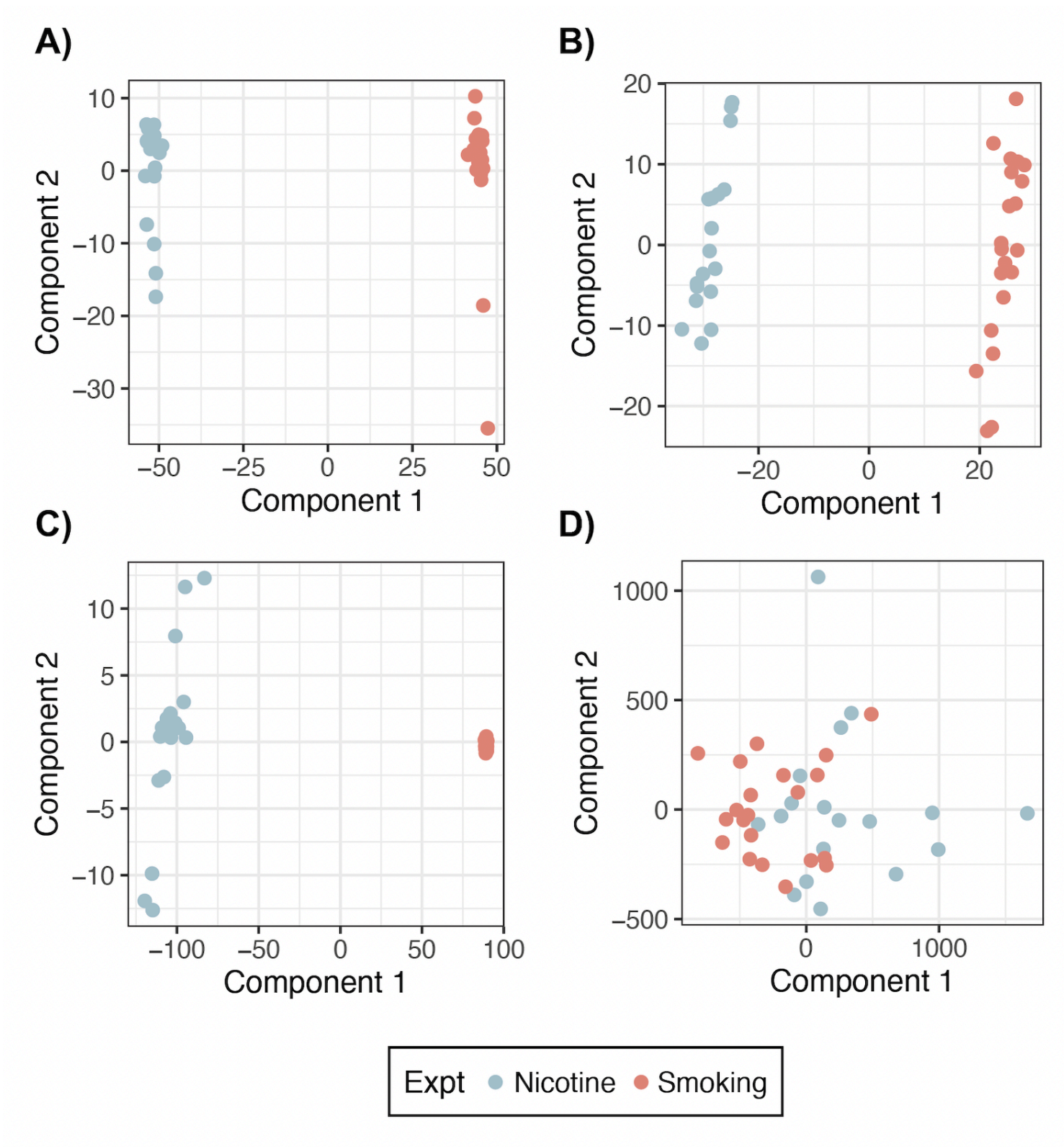

Supplementary Figure 4: Multidimensional scaling in adult brain samples. Component 1 vs Component 2 for **A)** gene, **B)** transcript, **C)** exon, and **D)** exon-exon junction expression in adult brain samples from the nicotine and smoking experiments, including exposed and control samples each. This analysis was done with samples that passed QC and manual sample filtering only (see **Fig. S1** and **Fig. S2**).

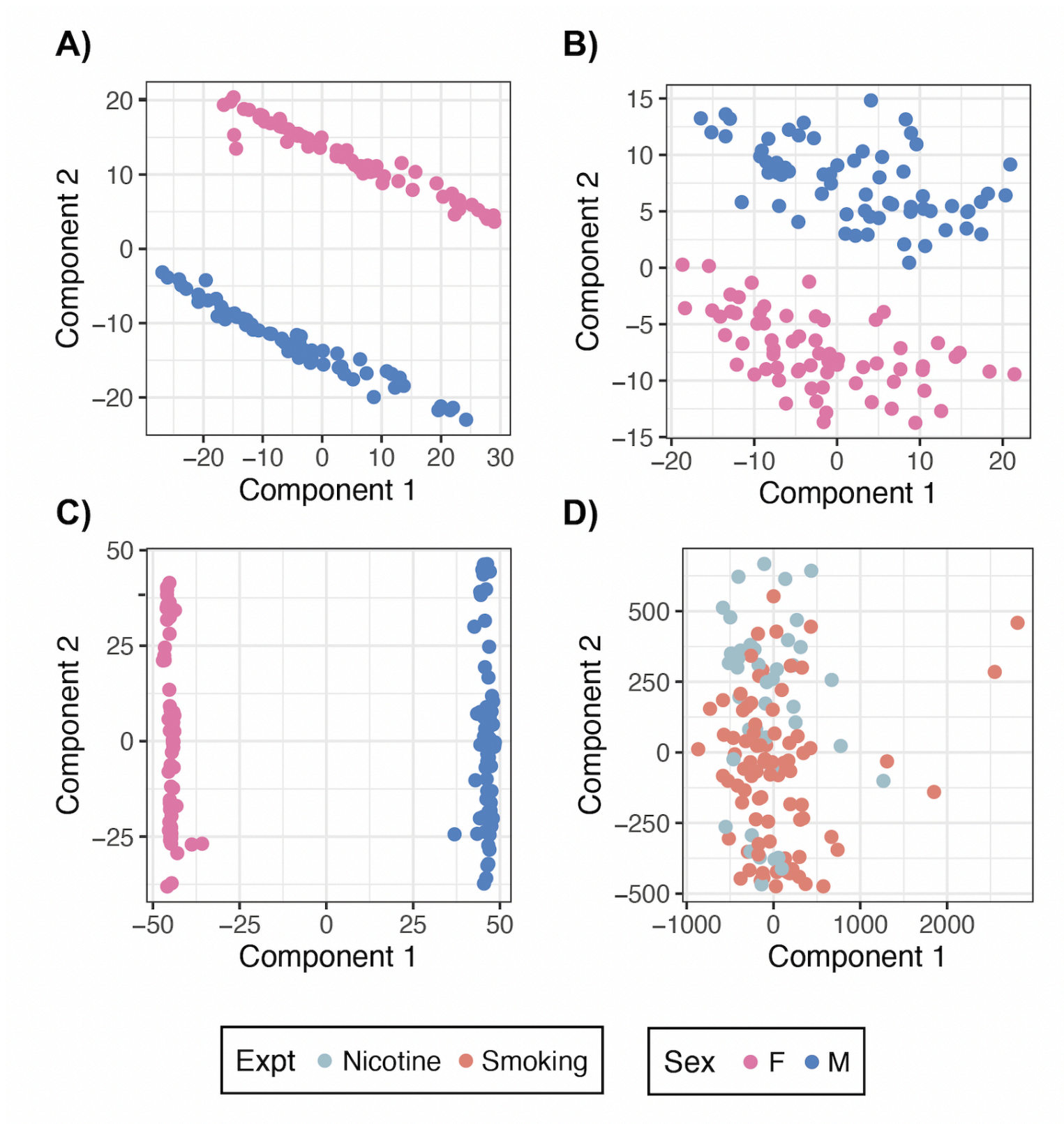

Supplementary Figure 5: Multidimensional scaling in pup brain samples. Component 1 vs Component 2 for **A)** gene, **B)** transcript, **C)** exon, and **D)** exon-exon junction expression in pup brain samples that passed QC and manual sample filtering (see **Fig. S1** and **Fig. S3**).

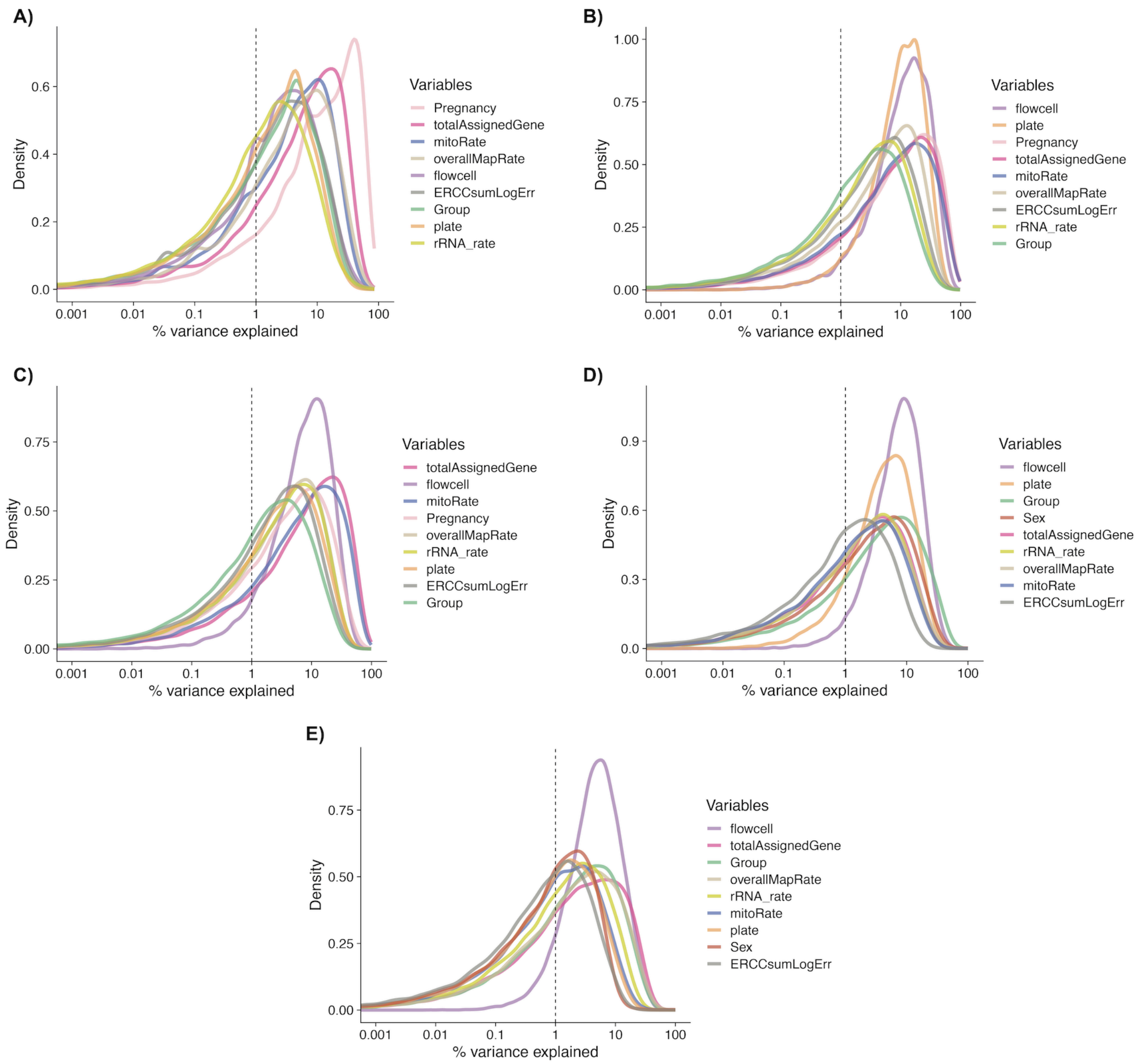

Supplementary Figure 6: Analysis of variance in gene expression explained by sample-level variables. Density plots for the percentage of variance in the expression of each gene explained by each variable in **A)** blood samples, **B)** adult brain samples from the nicotine and **C)** smoking experiment, and **D)** pup brain samples from the nicotine and **E)** smoking experiment. See **Table S19** for the description of these variables.

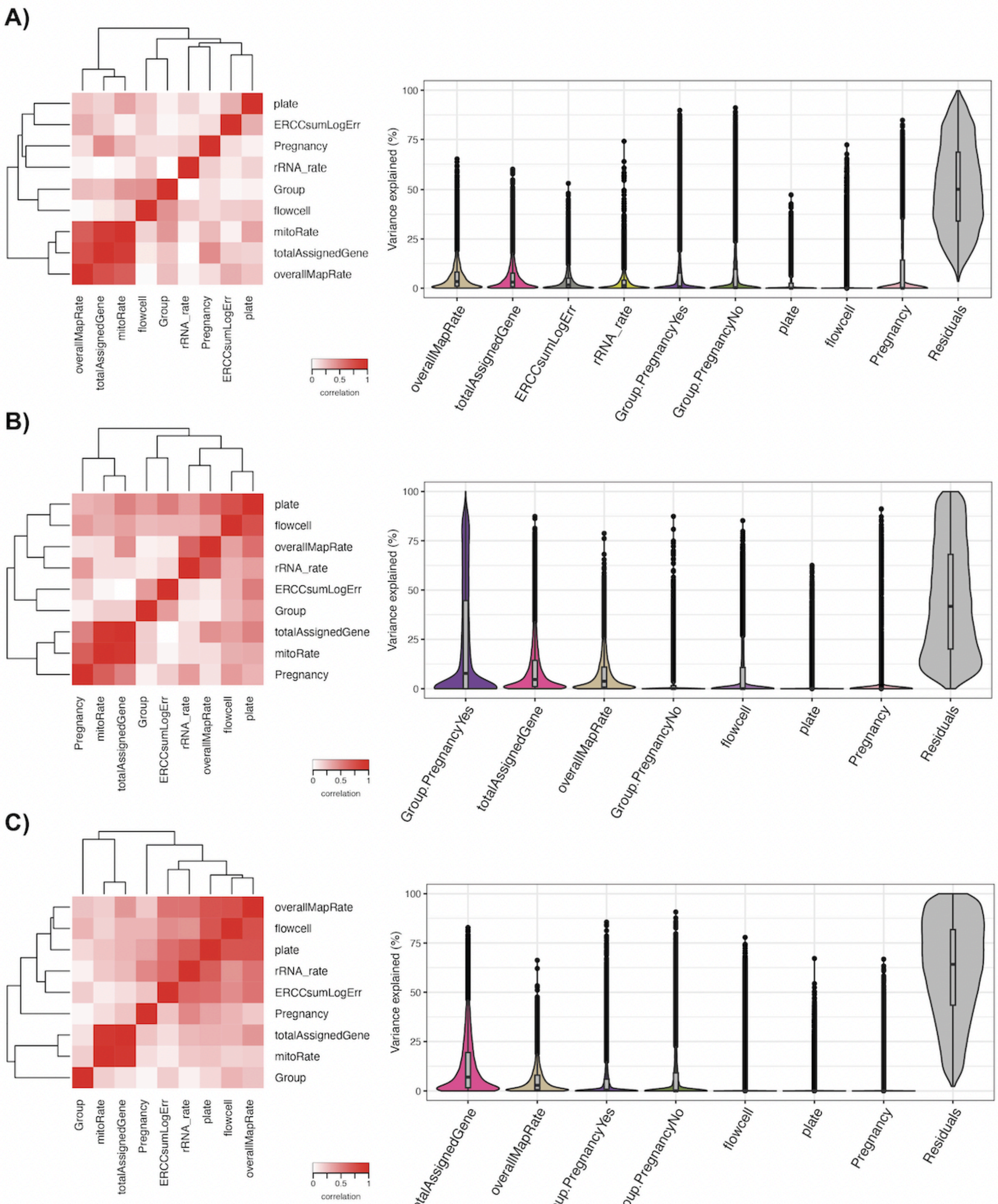
A)
plate
ERCCsumLogErr
Pregnancy
rRNA_rate
Group
flowcell
mitoRate
totalAssignedGene
overallMapRate
overallMapRate
totalAssignedGene
mitoRate
flowcell
Group
rRNA_rate
Pregnancy
ERCCsumLogErr
plate
0
0.5
1
correlation
Variance explained (%)
100
75
50
25
0
overallMapRate
totalAssignedGene
ERCCsumLogErr
rRNA_rate
Group.PregnancyYes
Group.PregnancyNo
plate
flowcell
Pregnancy
Residuals
B)
plate
flowcell
overallMapRate
rRNA_rate
ERCCsumLogErr
Group
totalAssignedGene
mitoRate
Pregnancy
Pregnancy
mitoRate
totalAssignedGene
Group
ERCCsumLogErr
rRNA_rate
overallMapRate
flowcell
plate
0
0.5
1
correlation
Variance explained (%)
100
75
50
25
0
Group.PregnancyYes
totalAssignedGene
overallMapRate
Group.PregnancyNo
flowcell
plate
Pregnancy
Residuals
C)
overallMapRate
flowcell
plate
rRNA_rate
ERCCsumLogErr
Pregnancy
totalAssignedGene
mitoRate
Group
Group
mitoRate
totalAssignedGene
Pregnancy
ERCCsumLogErr
rRNA_rate
plate
flowcell
overallMapRate
0
0.5
1
correlation
Variance explained (%)
100
75
50
25
0
totalAssignedGene
overallMapRate
Group.PregnancyYes
Group.PregnancyNo
flowcell
plate
Pregnancy
Residuals

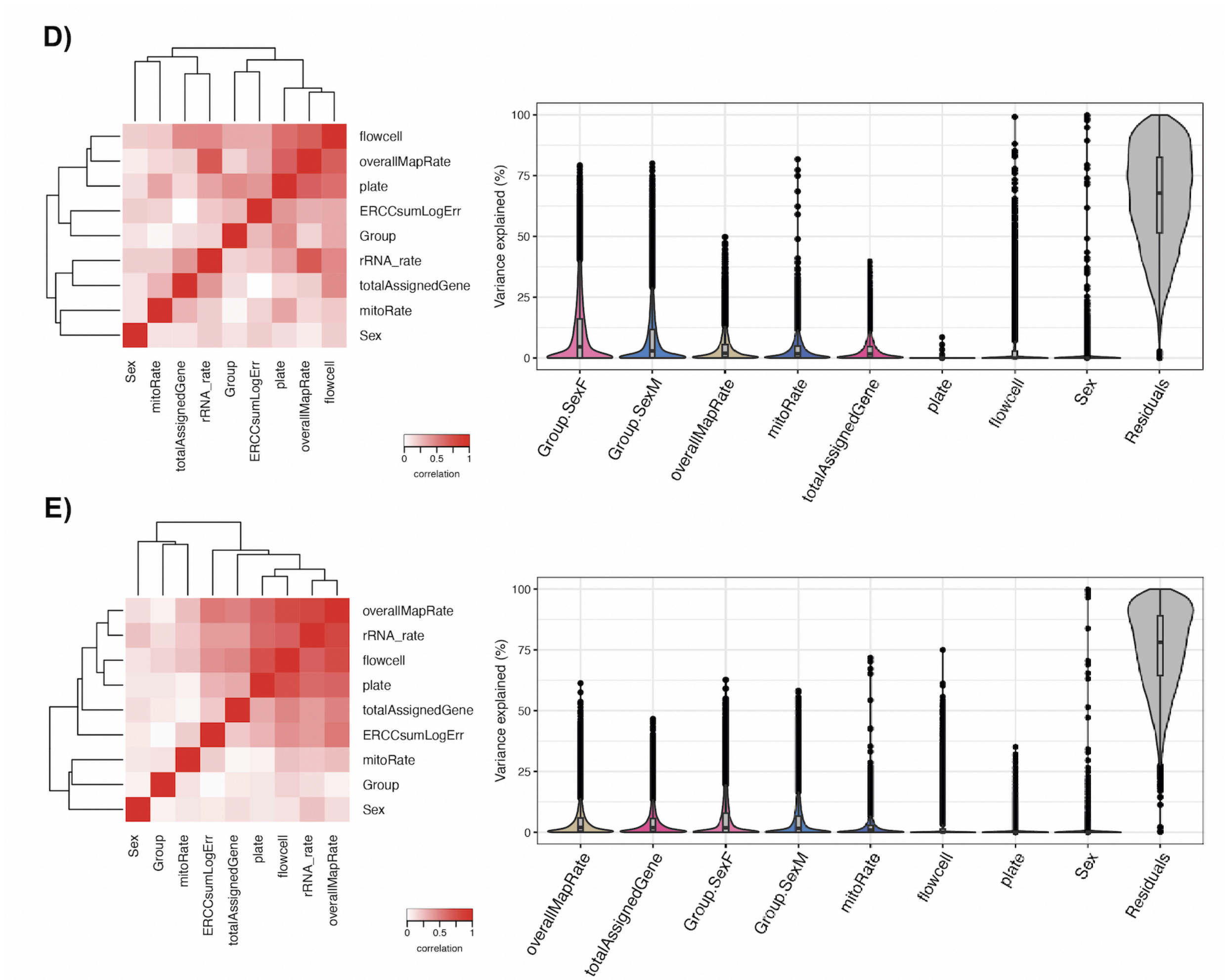

Supplementary Figure 7: Gene expression variance partition analysis. Left heat maps show the squared correlation between each pair of sample-level variables, obtained through a Canonical Correlation Analysis (CCA). Violin plots show, for each gene, the percentage of variance in its expression that is explained by each variable in **A)** blood samples of the smoking experiment, **B)** adult brain samples of the nicotine experiment, **C)** adult brain samples of the smoking experiment, **D)** pup brain samples of the nicotine experiment, and **E)** pup brain samples of the smoking experiment. Variables are ordered by decreasing mean fraction of variance explained (FVE). Variables in the heat maps not present in the corresponding violin plots were highly correlated with any other variable with a higher median FVE and thus were not included in the models for DEA. `ERCCsumLogErr` and `rRNA_rate` were not considered in the brain samples because their scales differ considerably and therefore were not suitable for variance partition. Labels `Group.Pregnancy[Yes/No]` in **A)-C)** and `Group.Sex[F/M]` in **D)** and **E)** refer to the contribution in gene expression variance of nicotine/smoking exposure (`Group`) within each pregnancy and sex group, respectively. See **Table S19** for the description of these sample variables. Residuals correspond to the fractions of gene expression variation that could not be attributed to any of the included sample-level variables.

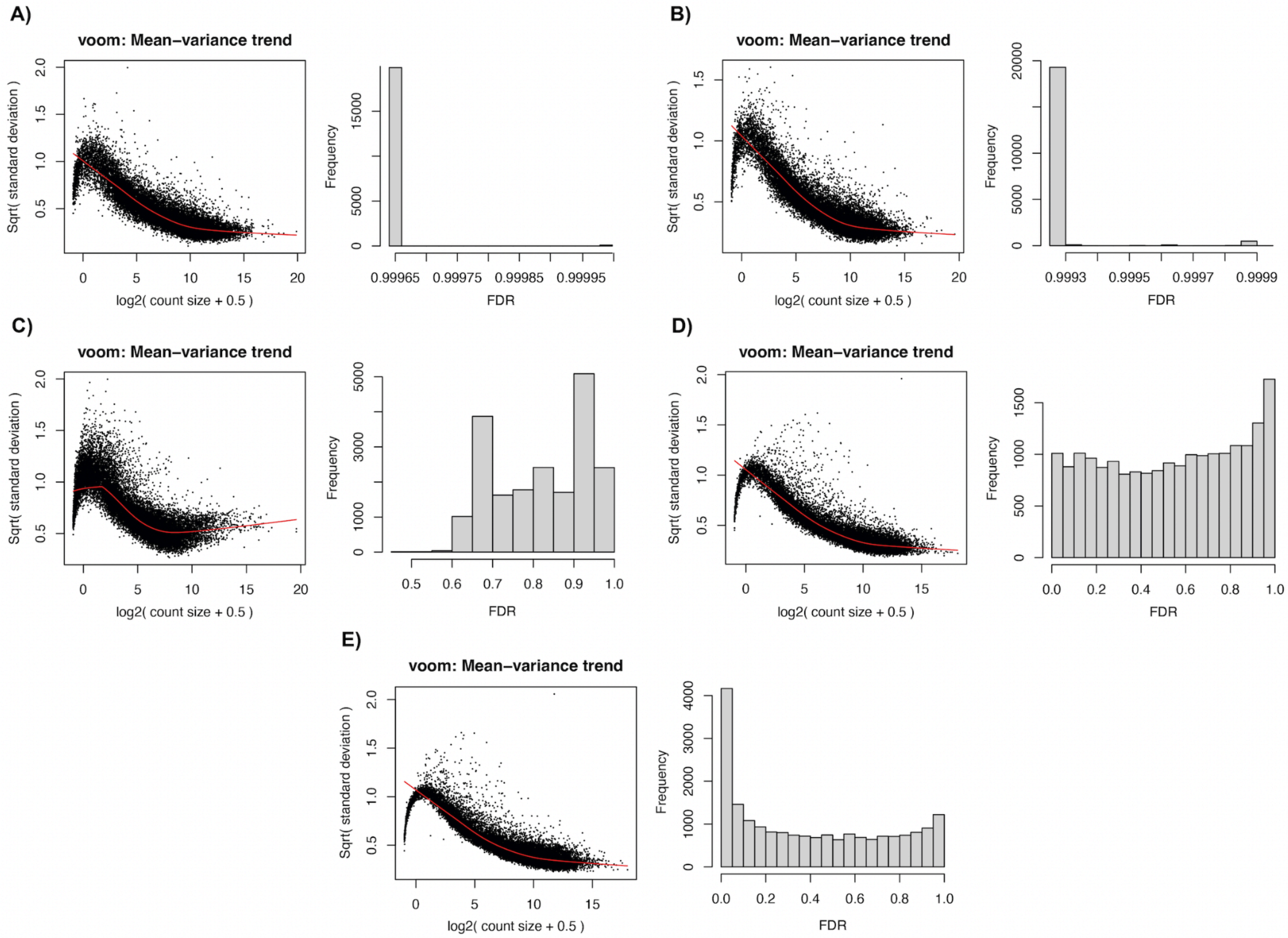

Supplementary Figure 8: **Differential gene expression analyses**. Left plots show across genes their mean expression (in $\log_2$-counts) compared to the square-root of their residual standard deviation in **A)** adult brain samples of the nicotine experiment, **B)** adult brain samples of the smoking experiment, **C)** adult blood samples of the smoking experiment, **D)** pup brain samples of the nicotine experiment, and **E)** pup brain samples of the smoking experiment. The red line corresponds to the global mean-variance trend. Histograms present FDR-adjusted *p*-values for differential expression in the same sample groups.

A)

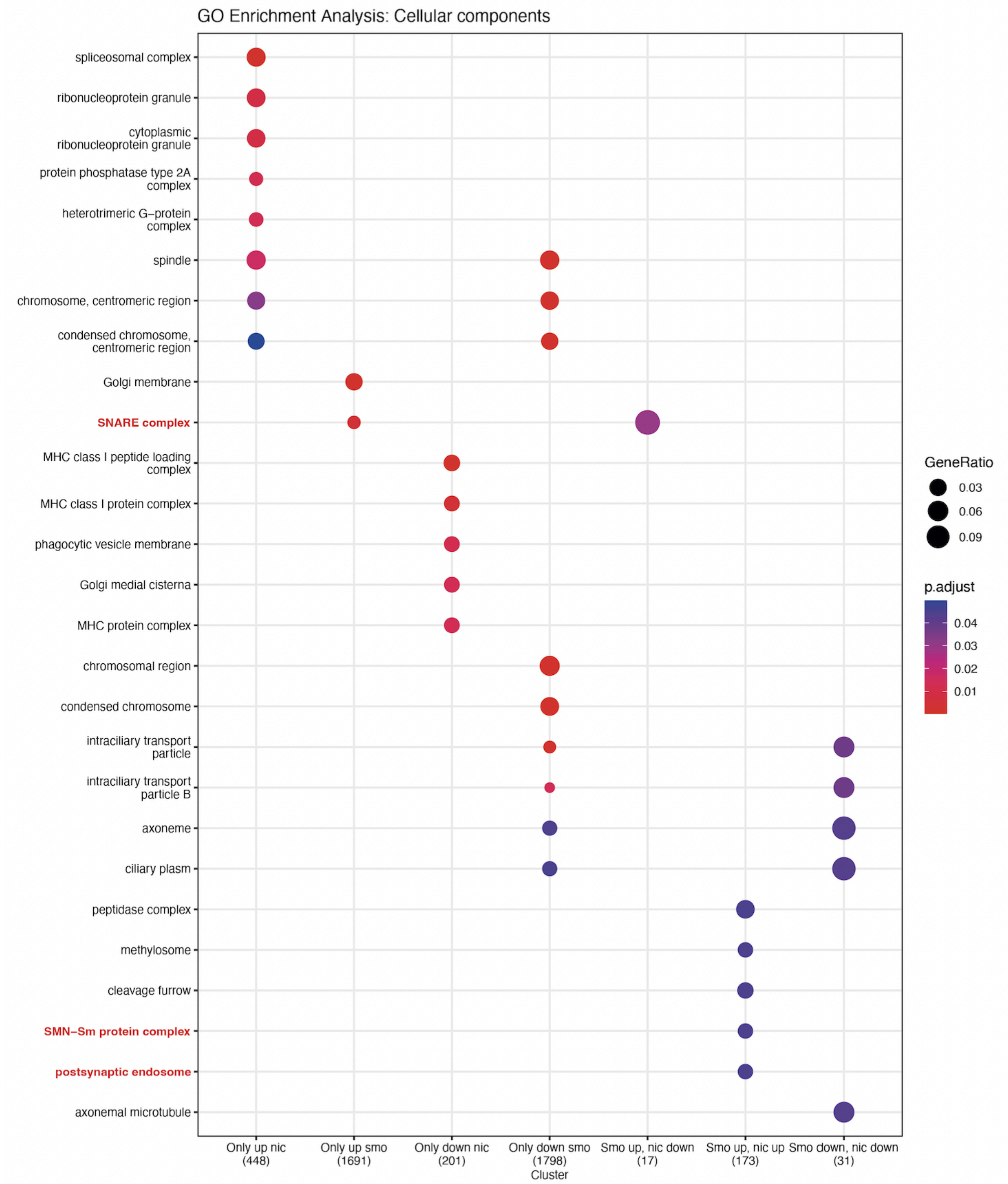

GO Enrichment Analysis: Cellular components
spliceosomal complex
ribonucleoprotein granule
cytoplasmic ribonucleoprotein granule
protein phosphatase type 2A complex
heterotrimeric G−protein complex
spindle
chromosome, centromeric region
condensed chromosome, centromeric region
Golgi membrane
SNARE complex
MHC class I peptide loading complex
MHC class I protein complex
phagocytic vesicle membrane
Golgi medial cisterna
MHC protein complex
chromosomal region
condensed chromosome
intraciliary transport particle
intraciliary transport particle B
axoneme
ciliary plasm
peptidase complex
methylosome
cleavage furrow
SMN−Sm protein complex
postsynaptic endosome
axonemal microtubule
Only up nic (448)
Only up smo (1691)
Only down nic (201)
Only down smo (1798)
Smo up, nic down (17)
Smo up, nic up (173)
Smo down, nic down (31)
Cluster
GeneRatio
0.03
0.06
0.09
p.adjust
0.04
0.03
0.02
0.01

B)

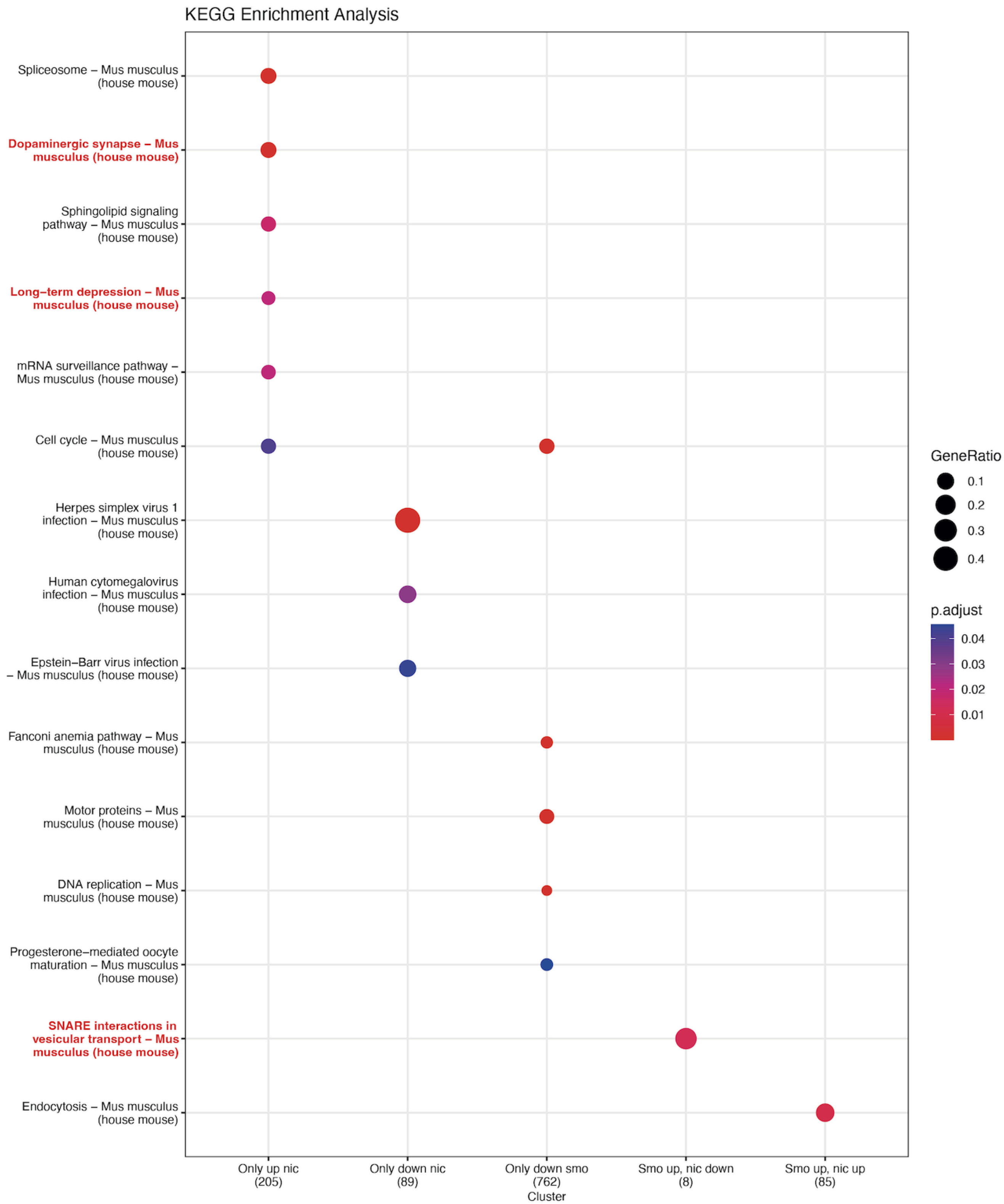
KEGG Enrichment Analysis
Spliceosome – Mus musculus (house mouse)
Dopaminergic synapse – Mus musculus (house mouse)
Sphingolipid signaling pathway – Mus musculus (house mouse)
Long–term depression – Mus musculus (house mouse)
mRNA surveillance pathway – Mus musculus (house mouse)
Cell cycle – Mus musculus (house mouse)
Herpes simplex virus 1 infection – Mus musculus (house mouse)
Human cytomegalovirus infection – Mus musculus (house mouse)
Epstein–Barr virus infection – Mus musculus (house mouse)
Fanconi anemia pathway – Mus musculus (house mouse)
Motor proteins – Mus musculus (house mouse)
DNA replication – Mus musculus (house mouse)
Progesterone–mediated oocyte maturation – Mus musculus (house mouse)
SNARE interactions in vesicular transport – Mus musculus (house mouse)
Endocytosis – Mus musculus (house mouse)
Only up nic (205)
Only down nic (89)
Only down smo (762)
Smo up, nic down (8)
Smo up, nic up (85)
Cluster
GeneRatio
0.1
0.2
0.3
0.4
p.adjust
0.04
0.03
0.02
0.01

C)

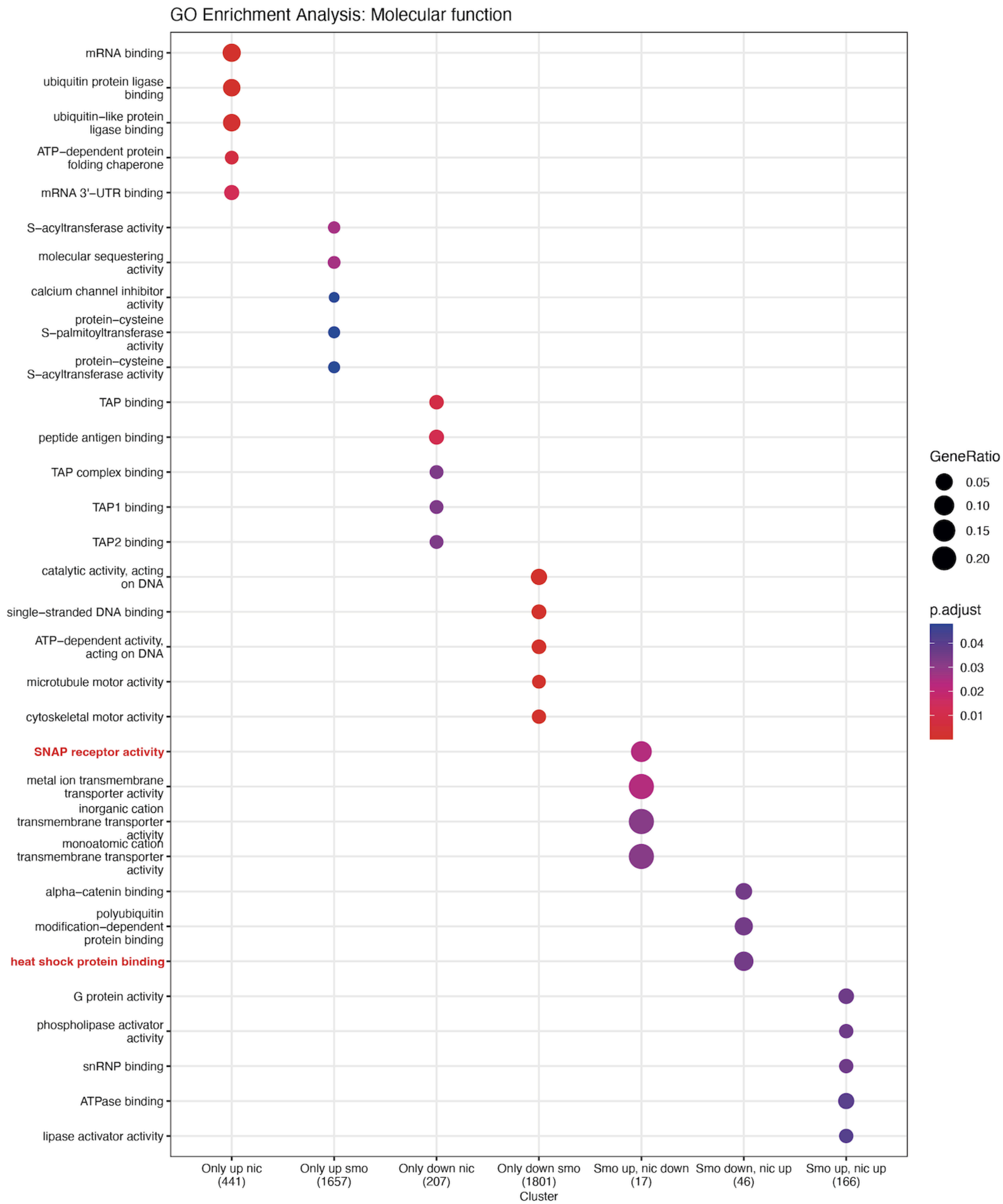

GO Enrichment Analysis: Molecular function
mRNA binding
ubiquitin protein ligase binding
ubiquitin−like protein ligase binding
ATP−dependent protein folding chaperone
mRNA 3'−UTR binding
S−acyltransferase activity
molecular sequestering activity
calcium channel inhibitor activity
protein−cysteine S−palmitoyltransferase activity
protein−cysteine S−acyltransferase activity
TAP binding
peptide antigen binding
TAP complex binding
TAP1 binding
TAP2 binding
catalytic activity, acting on DNA
single−stranded DNA binding
ATP−dependent activity, acting on DNA
microtubule motor activity
cytoskeletal motor activity
SNAP receptor activity
metal ion transmembrane transporter activity
inorganic cation transmembrane transporter activity
monoatomic cation transmembrane transporter activity
alpha−catenin binding
polyubiquitin modification−dependent protein binding
heat shock protein binding
G protein activity
phospholipase activator activity
snRNP binding
ATPase binding
lipase activator activity
Only up nic (441)
Only up smo (1657)
Only down nic (207)
Only down smo (1801)
Smo up, nic down (17)
Smo down, nic up (46)
Smo up, nic up (166)
Cluster
GeneRatio
0.05
0.10
0.15
0.20
p.adjust
0.04
0.03
0.02
0.01

D)

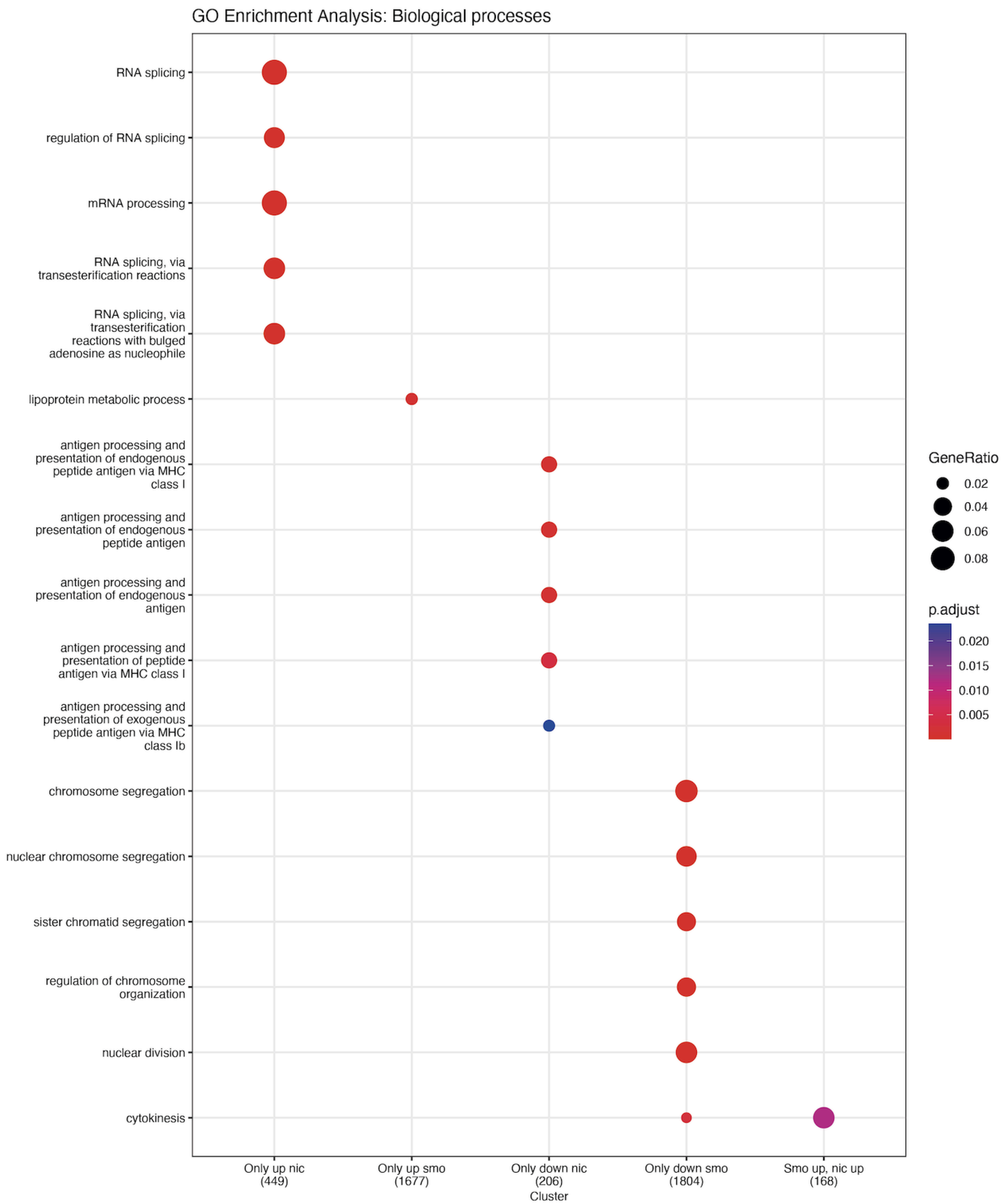

GO Enrichment Analysis: Biological processes
RNA splicing
regulation of RNA splicing
mRNA processing
RNA splicing, via transesterification reactions
RNA splicing, via transesterification reactions with bulged adenosine as nucleophile
lipoprotein metabolic process
antigen processing and presentation of endogenous peptide antigen via MHC class I
antigen processing and presentation of endogenous peptide antigen
antigen processing and presentation of endogenous antigen
antigen processing and presentation of peptide antigen via MHC class I
antigen processing and presentation of exogenous peptide antigen via MHC class Ib
chromosome segregation
nuclear chromosome segregation
sister chromatid segregation
regulation of chromosome organization
nuclear division
cytokinesis
Only up nic (449)
Only up smo (1677)
Only down nic (206)
Only down smo (1804)
Smo up, nic up (168)
Cluster
GeneRatio
0.02
0.04
0.06
0.08
p.adjust
0.020
0.015
0.010
0.005

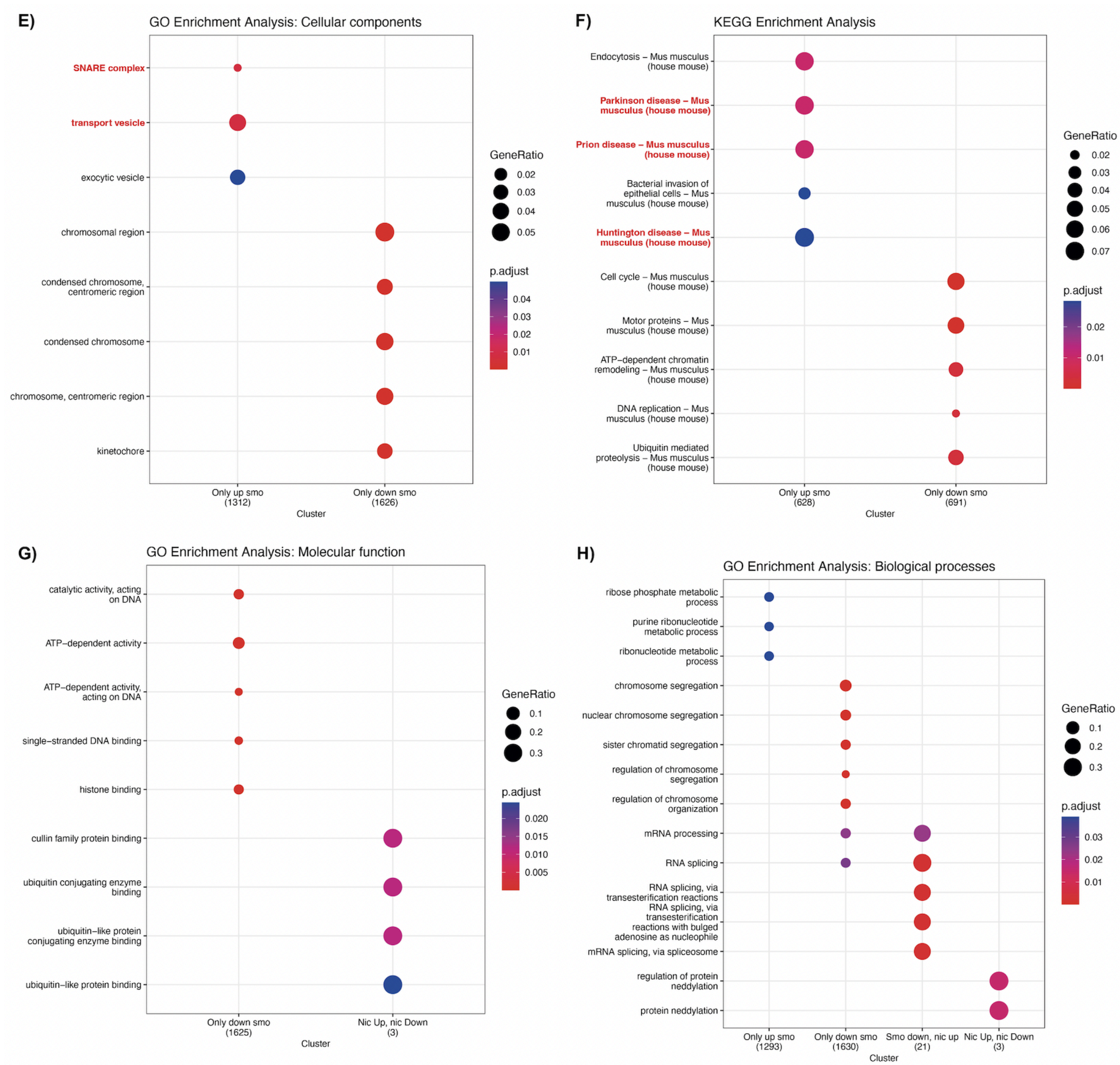

Supplementary Figure 9: Functional enrichment analysis for DEGs and genes expressing DE transcripts in the pup brain. Cellular components (CC), KEGG pathways, molecular functions (MF), and biological processes (BP) significantly overrepresented (FDR-adjusted *p*-value<0.05) in the clusters of (**A-D**) DEGs and (**E-H**) genes expressing DE transcripts, indicated on the x-axis. Clusters without significant results are excluded. `Up` and `down` labels stand for upregulated and downregulated, respectively, and `only` refers to genes that were significantly affected (**A-D**) or had significantly affected transcripts (**E-H**) in either the smoking (`smo`) or nicotine (`nic`) experiment only. Note that cluster numbers (in parentheses) correspond to the number of genes in the specified cluster that are annotated in at least one GO/KEGG term. Gene ratio is the number of genes in each cluster annotated in a term (y-axis) over the total in the respective cluster. Only the top 5 most significant enriched terms are reported per cluster, unless they share additional significant terms with any other clusters. Terms of interest appear in red (see genes involved in each in **Fig. S10**). Related to **Fig. 2**.

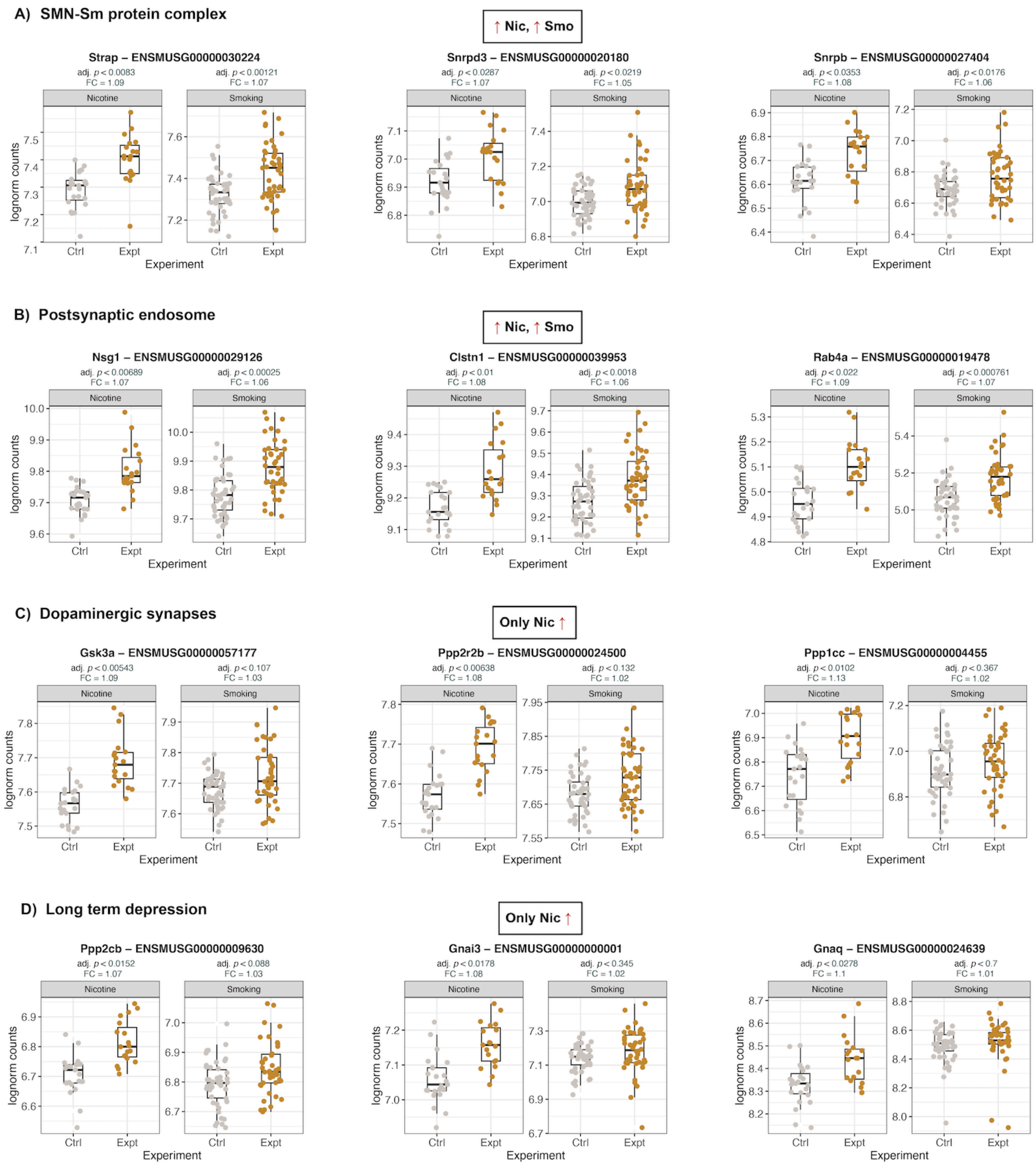
A) SMN-Sm protein complex
↑ Nic, ↑ Smo
Strap – ENSMUSG00000030224
adj. p < 0.0083
FC = 1.09
adj. p < 0.00121
FC = 1.07
Snrpd3 – ENSMUSG00000020180
adj. p < 0.0287
FC = 1.07
adj. p < 0.0219
FC = 1.05
Snrpb – ENSMUSG00000027404
adj. p < 0.0353
FC = 1.08
adj. p < 0.0176
FC = 1.06
B) Postsynaptic endosome
↑ Nic, ↑ Smo
Nsg1 – ENSMUSG00000029126
adj. p < 0.00689
FC = 1.07
adj. p < 0.00025
FC = 1.06
Clstn1 – ENSMUSG00000039953
adj. p < 0.01
FC = 1.08
adj. p < 0.0018
FC = 1.06
Rab4a – ENSMUSG00000019478
adj. p < 0.022
FC = 1.09
adj. p < 0.000761
FC = 1.07
C) Dopaminergic synapses
Only Nic ↑
Gsk3a – ENSMUSG00000057177
adj. p < 0.00543
FC = 1.09
adj. p < 0.107
FC = 1.03
Ppp2r2b – ENSMUSG00000024500
adj. p < 0.00638
FC = 1.08
adj. p < 0.132
FC = 1.02
Ppp1cc – ENSMUSG00000004455
adj. p < 0.0102
FC = 1.13
adj. p < 0.367
FC = 1.02
D) Long term depression
Only Nic ↑
Ppp2cb – ENSMUSG00000009630
adj. p < 0.0152
FC = 1.07
adj. p < 0.088
FC = 1.03
Gnai3 – ENSMUSG00000000001
adj. p < 0.0178
FC = 1.08
adj. p < 0.345
FC = 1.02
Gnaq – ENSMUSG00000024639
adj. p < 0.0278
FC = 1.1
adj. p < 0.7
FC = 1.01
Nicotine
Smoking
lognorm counts
Ctrl
Expt
Experiment

E) SNARE complex

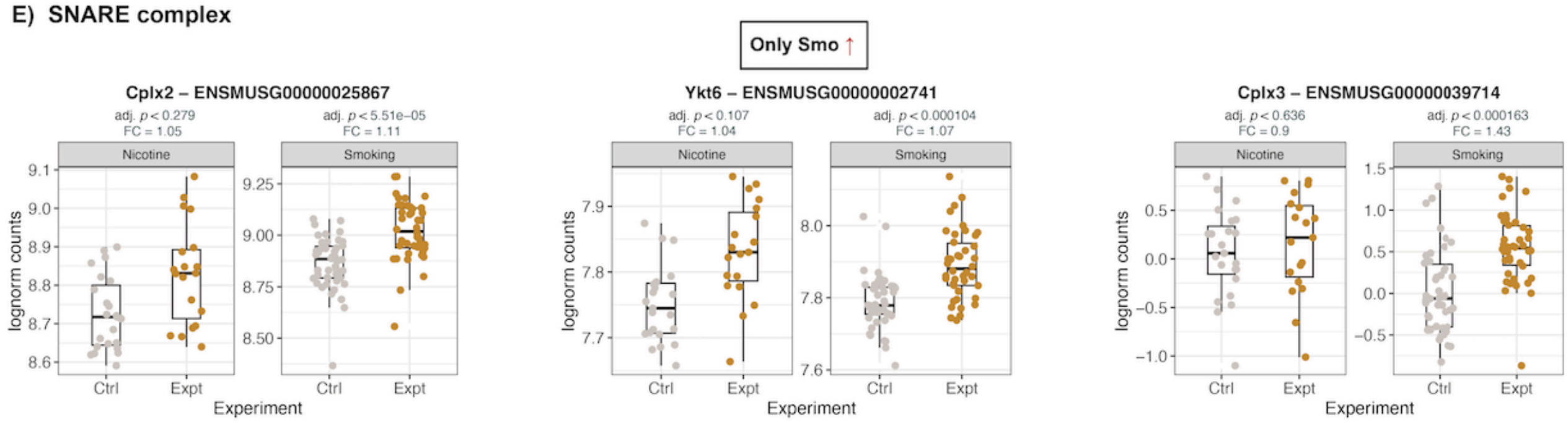

F) SNARE complex, SNARE interactions in vesicle transport and SNAP receptor activity

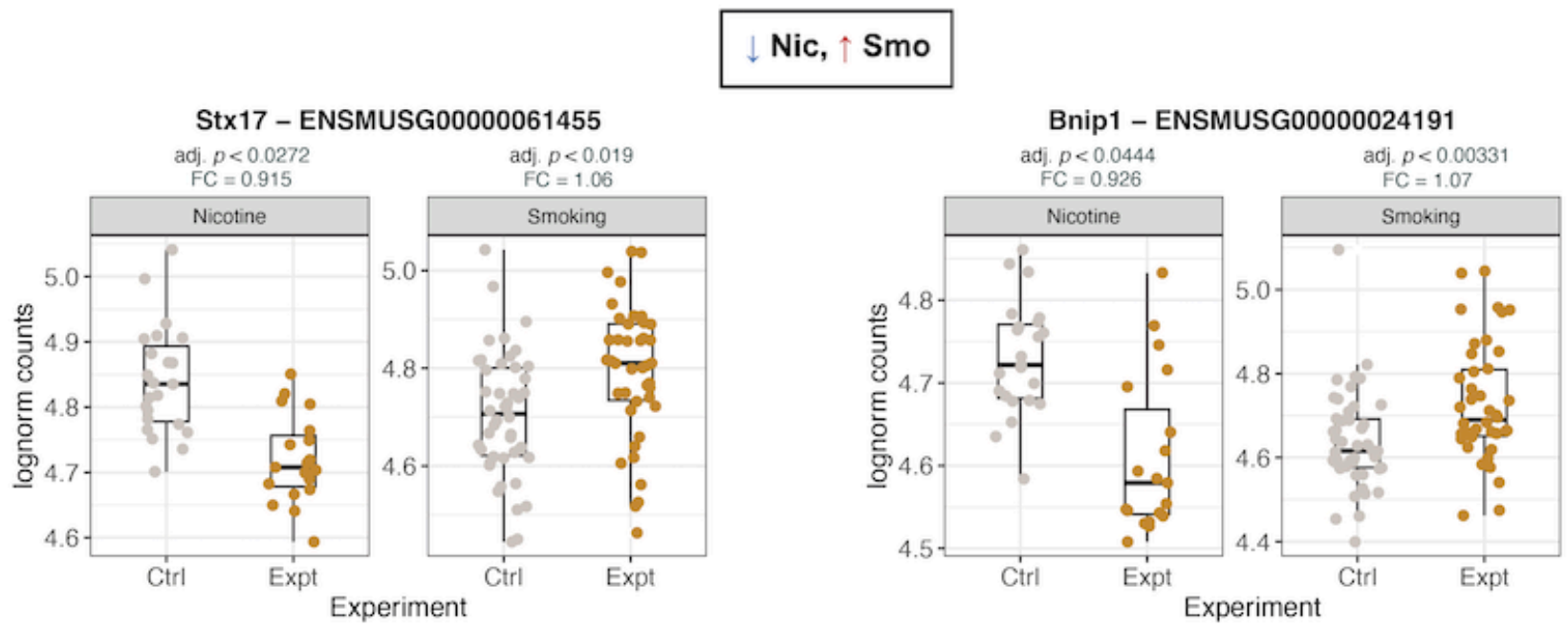

G) Heat shock protein binding activity

↑ Nic, ↓ Smo

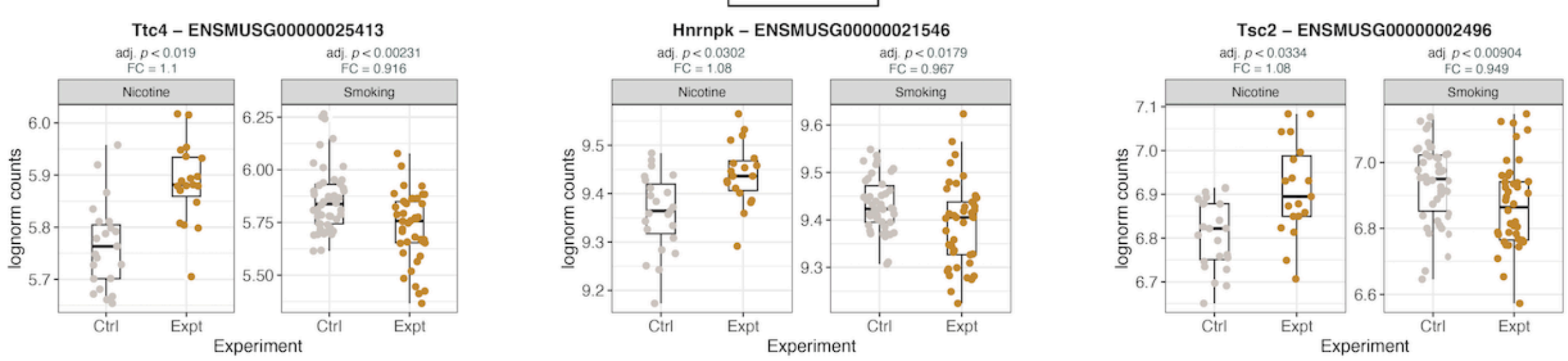

H) Transport vesicles

Only Smo ↑

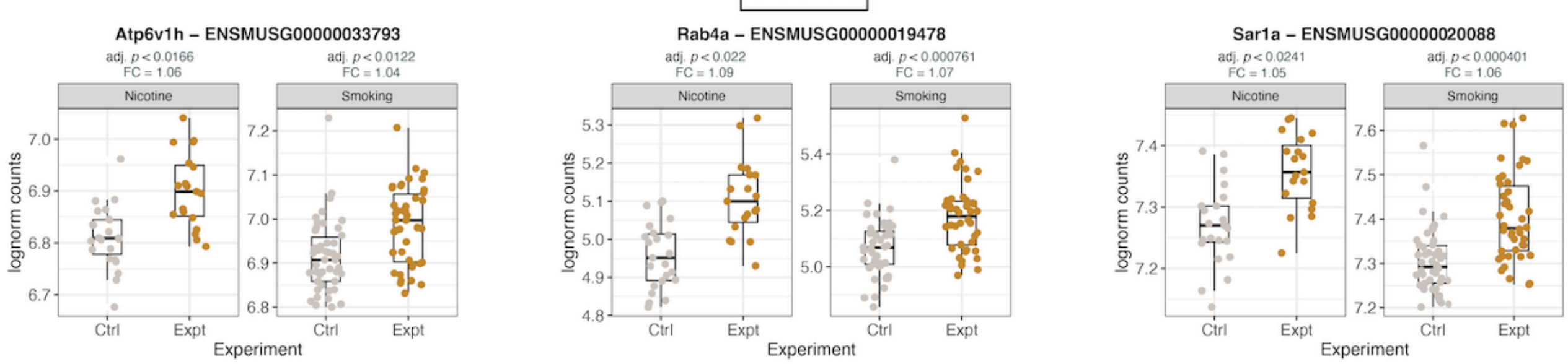

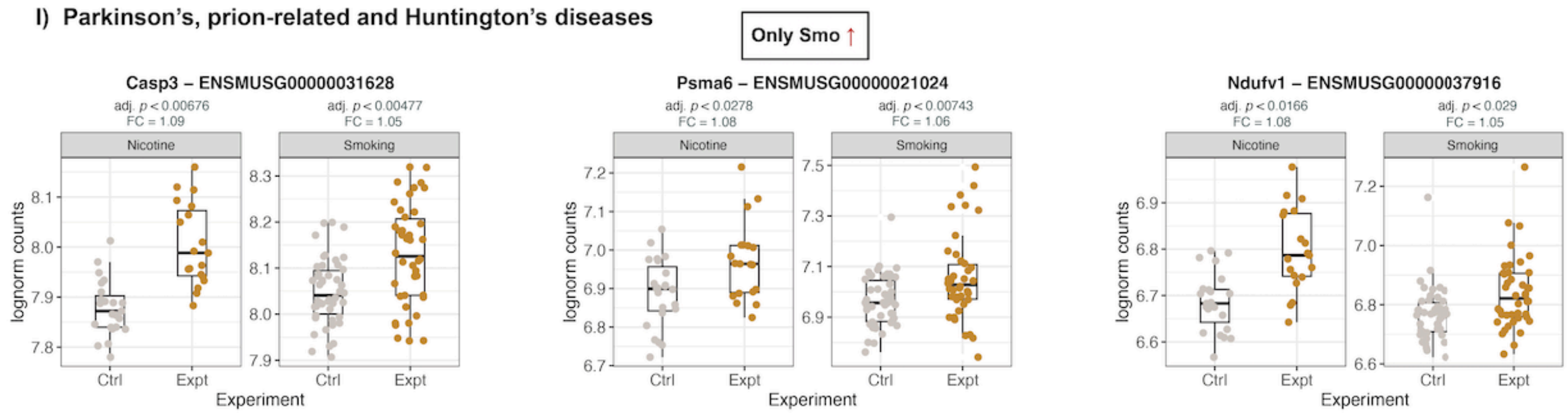

Supplementary Figure 10: **Expression of genes implicated in processes of interest in the pup brain**. Gene lognorm-counts of **A)** the top 3 most significant DEGs upregulated in nicotine and smoking-exposed samples that act in SMN-Sm protein complexes and **B)** postsynaptic endosomes. **C)** The top 3 most significant DEGs upregulated in nicotine-exposed samples only that are involved in dopaminergic synapses and **D)** in long-term depression. **E)** The top 3 most significant DEGs whose products work in the SNARE complex and are upregulated in smoking-exposed samples only (expressing DE transcripts also) or **F)** are upregulated under smoking exposure and downregulated under nicotine exposure and also have SNAP receptor activity and are implicated in SNARE interactions in vesicle transport. **G)** The top 3 most significant DEGs upregulated under nicotine exposure and downregulated under smoking exposure, whose gene products have heat shock protein binding activity. **H)** The top 3 most significant genes expressing DE transcripts that were upregulated under smoking only and act in transport vesicles and **I)** are involved in Parkinson's, prion-related, and Huntington's diseases. Related to **Fig. 2** and **Fig. S9**.

FDR: false discovery rate; FC: fold-change; Ctrl: control samples; Expt: experimental (exposed) samples.

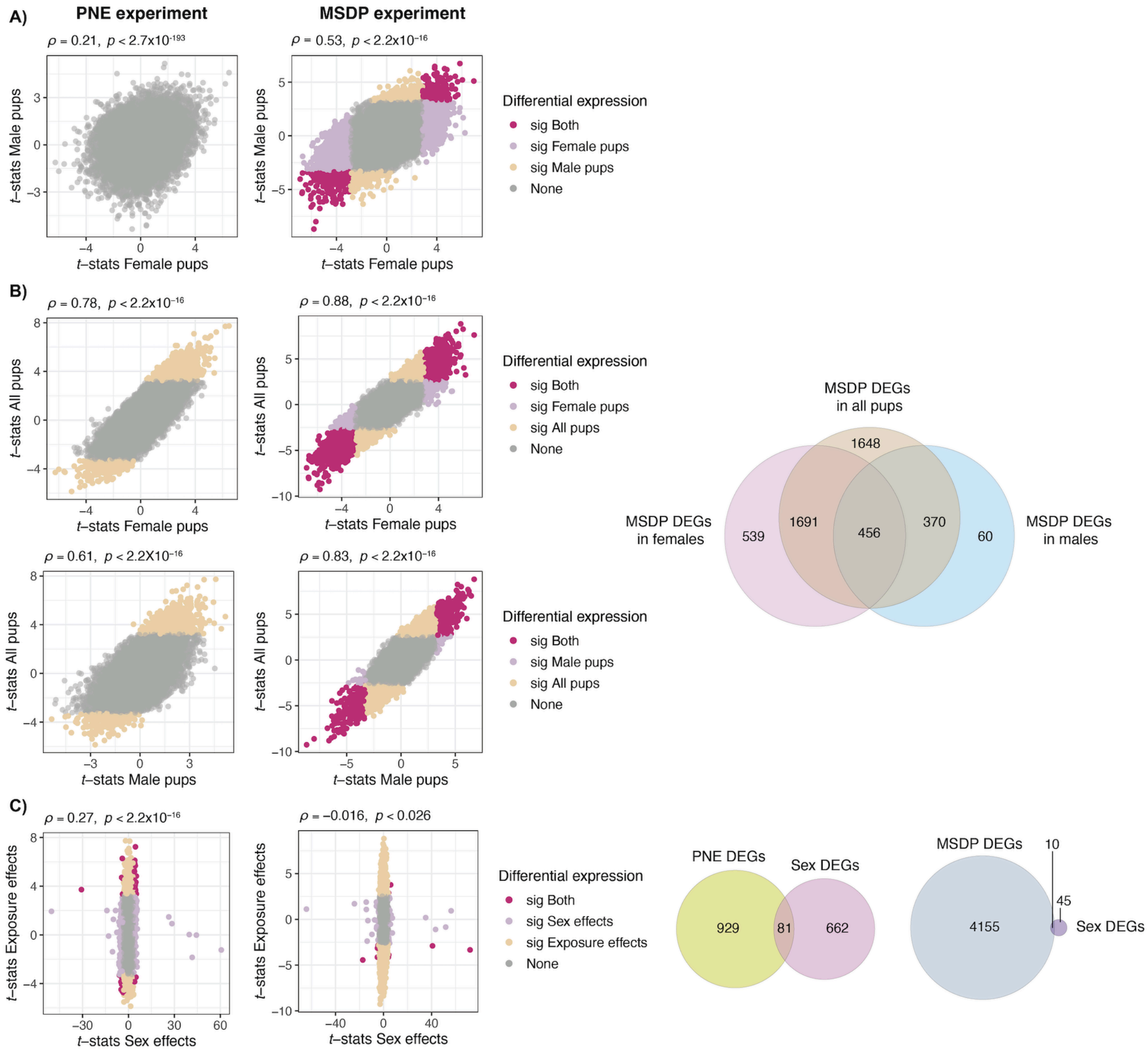

Supplementary Figure 11: Sex differences in transcriptomic effects of nicotine and smoking exposure in the developing brain. A) Moderated *t*-statistics for nicotine (left) and smoking (right) exposure in female vs. male pups. In dark pink, genes significantly affected in both sexes; in light pink and beige, genes significantly affected only in females and males, respectively, and in gray, genes that were not affected. **B)** Moderated *t*-statistics for same exposures in female pups (top scatter plots) and male pups (bottom scatter plots), compared to effects observed in all female and male pups together. The Venn diagram shows the number of MSDP DEGs found in female pups only, male pups only, and common to both sexes, and their overlaps with MSDP DEGs detected analyzing all pups together. **C)** Moderated *t*-statistics for same exposures compared to *t*-statistics for sex DGE. The Venn diagrams show the overlap between PNE and sex DEGs (left), and MSDP and sex DEGs (right). Spearman's correlation coefficient (ϱ) between *t*-statistics and its corresponding *p*-value are shown.

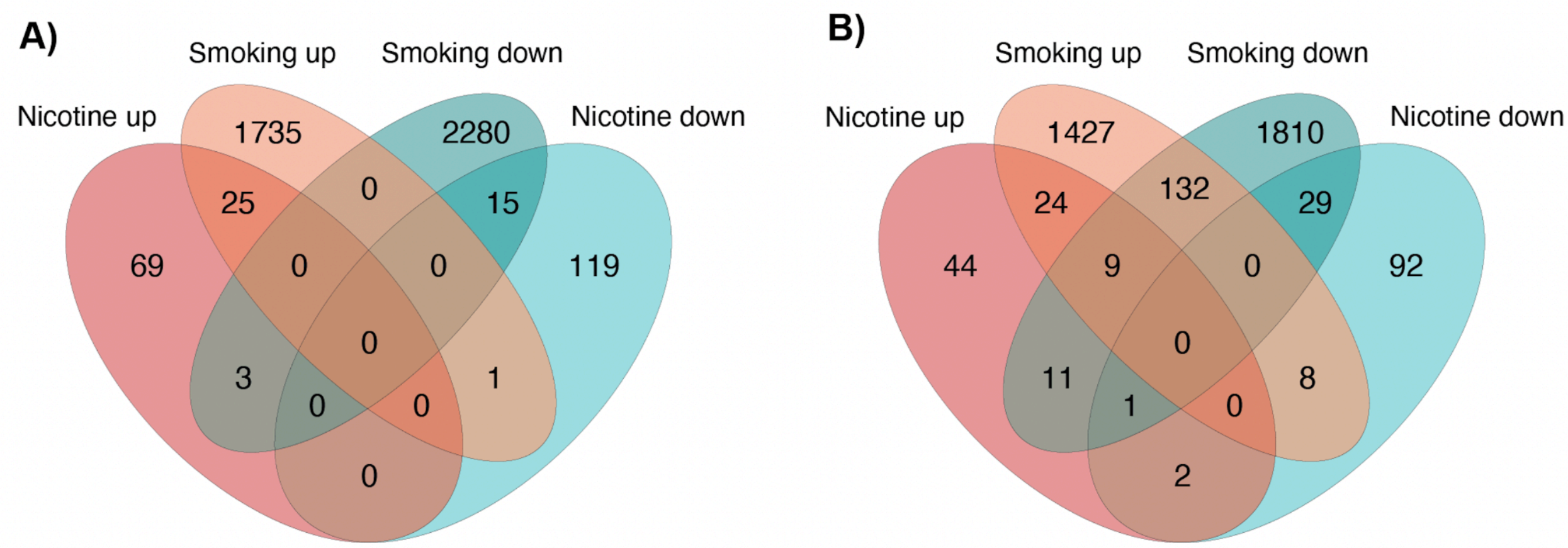

Supplementary Figure 12: Results of the DTE analysis in pup brain. Number of **A)** DE transcripts and **B)** genes expressing DE transcripts, up- and down-regulated in the nicotine and smoking exposure experiments.

A)

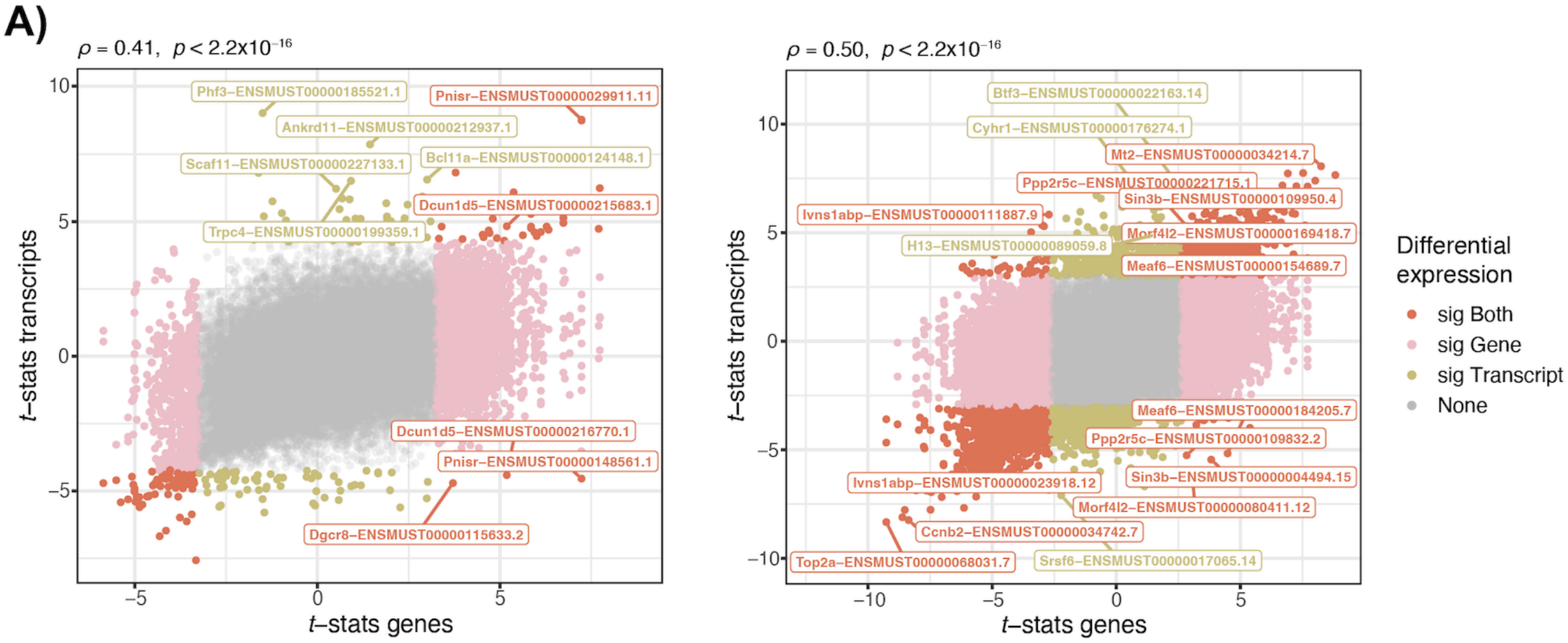
ρ = 0.41, p < 2.2x10^-16
Phf3−ENSMUST00000185521.1
Pnisr−ENSMUST00000029911.11
Ankrd11−ENSMUST00000212937.1
Scaf11−ENSMUST00000227133.1
Bcl11a−ENSMUST00000124148.1
Dcun1d5−ENSMUST00000215683.1
Trpc4−ENSMUST00000199359.1
Dcun1d5−ENSMUST00000216770.1
Pnisr−ENSMUST00000148561.1
Dgcr8−ENSMUST00000115633.2
t−stats transcripts
t−stats genes
ρ = 0.50, p < 2.2x10^-16
Btf3−ENSMUST00000022163.14
Cyhr1−ENSMUST00000176274.1
Mt2−ENSMUST00000034214.7
Ppp2r5c−ENSMUST00000221715.1
Sin3b−ENSMUST00000109950.4
Ivns1abp−ENSMUST00000111887.9
Morf4l2−ENSMUST00000169418.7
H13−ENSMUST00000089059.8
Meaf6−ENSMUST00000154689.7
Meaf6−ENSMUST00000184205.7
Ppp2r5c−ENSMUST00000109832.2
Sin3b−ENSMUST00000004494.15
Ivns1abp−ENSMUST00000023918.12
Morf4l2−ENSMUST00000080411.12
Ccnb2−ENSMUST00000034742.7
Top2a−ENSMUST00000068031.7
Srsf6−ENSMUST00000017065.14
Differential expression
sig Both
sig Gene
sig Transcript
None

B)

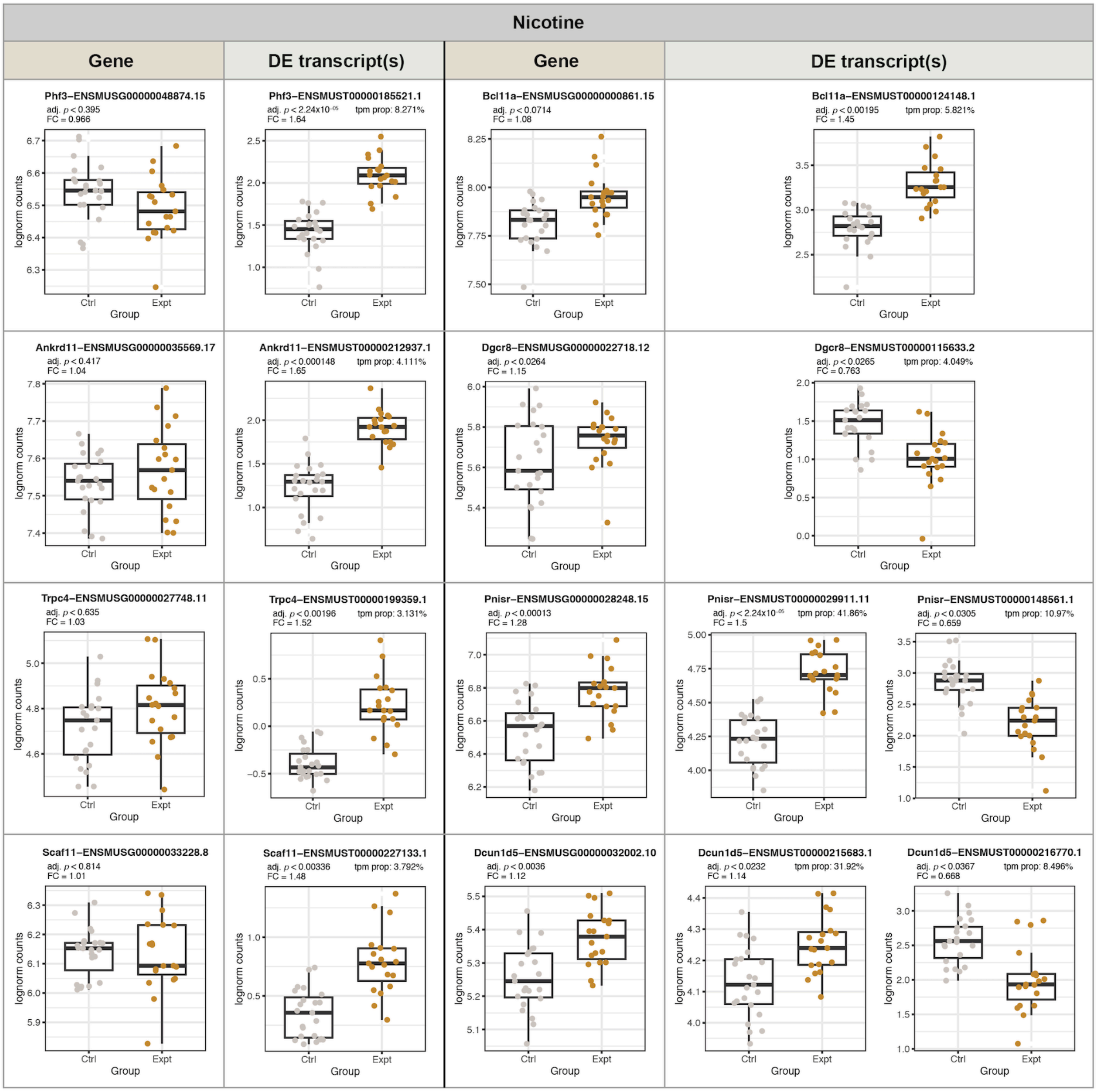

Nicotine
Gene
DE transcript(s)
Gene
DE transcript(s)
Phf3–ENSMUSG00000048874.15
adj. p < 0.395
FC = 0.966
Phf3–ENSMUST00000185521.1
adj. p < 2.24x10⁻⁰⁵ tpm prop: 8.271%
FC = 1.64
Bcl11a–ENSMUSG00000000861.15
adj. p < 0.0714
FC = 1.08
Bcl11a–ENSMUST00000124148.1
adj. p < 0.00195 tpm prop: 5.821%
FC = 1.45
Ankrd11–ENSMUSG00000035569.17
adj. p < 0.417
FC = 1.04
Ankrd11–ENSMUST00000212937.1
adj. p < 0.000148 tpm prop: 4.111%
FC = 1.65
Dgcr8–ENSMUSG00000022718.12
adj. p < 0.0264
FC = 1.15
Dgcr8–ENSMUST00000115633.2
adj. p < 0.0265 tpm prop: 4.049%
FC = 0.763
Trpc4–ENSMUSG00000027748.11
adj. p < 0.635
FC = 1.03
Trpc4–ENSMUST00000199359.1
adj. p < 0.00196 tpm prop: 3.131%
FC = 1.52
Pnisr–ENSMUSG00000028248.15
adj. p < 0.00013
FC = 1.28
Pnisr–ENSMUST00000029911.11
adj. p < 2.24x10⁻⁰⁵ tpm prop: 41.86%
FC = 1.5
Pnisr–ENSMUST00000148561.1
adj. p < 0.0305 tpm prop: 10.97%
FC = 0.659
Scaf11–ENSMUSG00000033228.8
adj. p < 0.814
FC = 1.01
Scaf11–ENSMUST00000227133.1
adj. p < 0.00336 tpm prop: 3.792%
FC = 1.48
Dcun1d5–ENSMUSG00000032002.10
adj. p < 0.0036
FC = 1.12
Dcun1d5–ENSMUST00000215683.1
adj. p < 0.0232 tpm prop: 31.92%
FC = 1.14
Dcun1d5–ENSMUST00000216770.1
adj. p < 0.0367 tpm prop: 8.496%
FC = 0.668
lognorm counts
Ctrl
Expt
Group

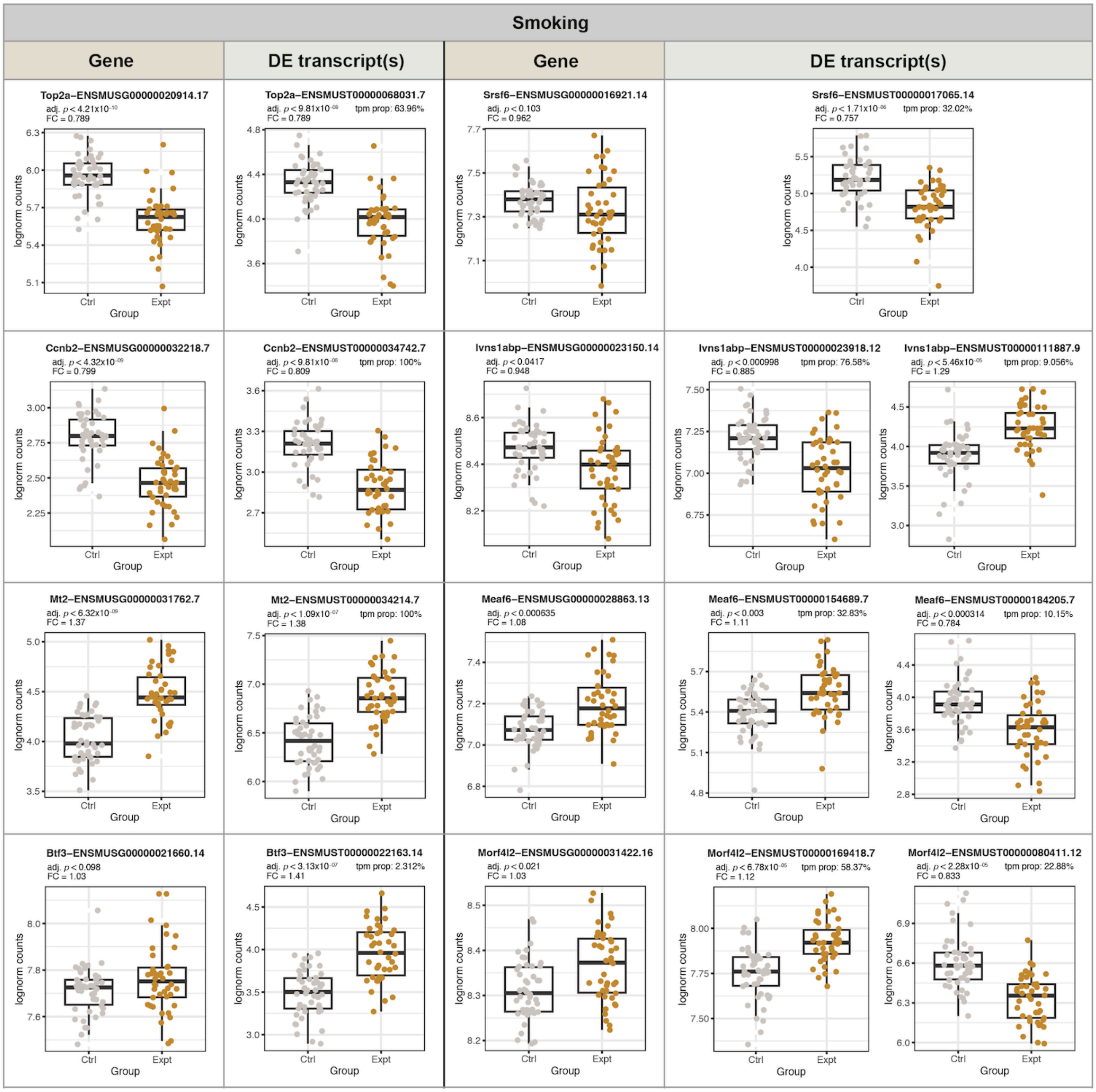

Smoking
Gene
DE transcript(s)
Gene
DE transcript(s)
Top2a–ENSMUSG00000020914.17
adj. p < 4.21x10-10
FC = 0.789
Top2a–ENSMUST00000068031.7
adj. p < 9.81x10-08 tpm prop: 63.96%
FC = 0.789
Srsf6–ENSMUSG00000016921.14
adj. p < 0.103
FC = 0.962
Srsf6–ENSMUST00000017065.14
adj. p < 1.71x10-06 tpm prop: 32.02%
FC = 0.757
Ccnb2–ENSMUSG00000032218.7
adj. p < 4.32x10-09
FC = 0.799
Ccnb2–ENSMUST00000034742.7
adj. p < 9.81x10-08 tpm prop: 100%
FC = 0.809
Ivns1abp–ENSMUSG00000023150.14
adj. p < 0.0417
FC = 0.948
Ivns1abp–ENSMUST00000023918.12
adj. p < 0.000998 tpm prop: 76.58%
FC = 0.885
Ivns1abp–ENSMUST00000111887.9
adj. p < 5.46x10-05 tpm prop: 9.056%
FC = 1.29
Mt2–ENSMUSG00000031762.7
adj. p < 6.32x10-09
FC = 1.37
Mt2–ENSMUST00000034214.7
adj. p < 1.09x10-07 tpm prop: 100%
FC = 1.38
Meaf6–ENSMUSG00000028863.13
adj. p < 0.000635
FC = 1.08
Meaf6–ENSMUST00000154689.7
adj. p < 0.003 tpm prop: 32.83%
FC = 1.11
Meaf6–ENSMUST00000184205.7
adj. p < 0.000314 tpm prop: 10.15%
FC = 0.784
Btf3–ENSMUSG00000021660.14
adj. p < 0.098
FC = 1.03
Btf3–ENSMUST00000022163.14
adj. p < 3.13x10-07 tpm prop: 2.312%
FC = 1.41
Morf4l2–ENSMUSG00000031422.16
adj. p < 0.021
FC = 1.03
Morf4l2–ENSMUST00000169418.7
adj. p < 6.78x10-05 tpm prop: 58.37%
FC = 1.12
Morf4l2–ENSMUST00000080411.12
adj. p < 2.28x10-05 tpm prop: 22.88%
FC = 0.833
lognorm counts
Group
Ctrl
Expt

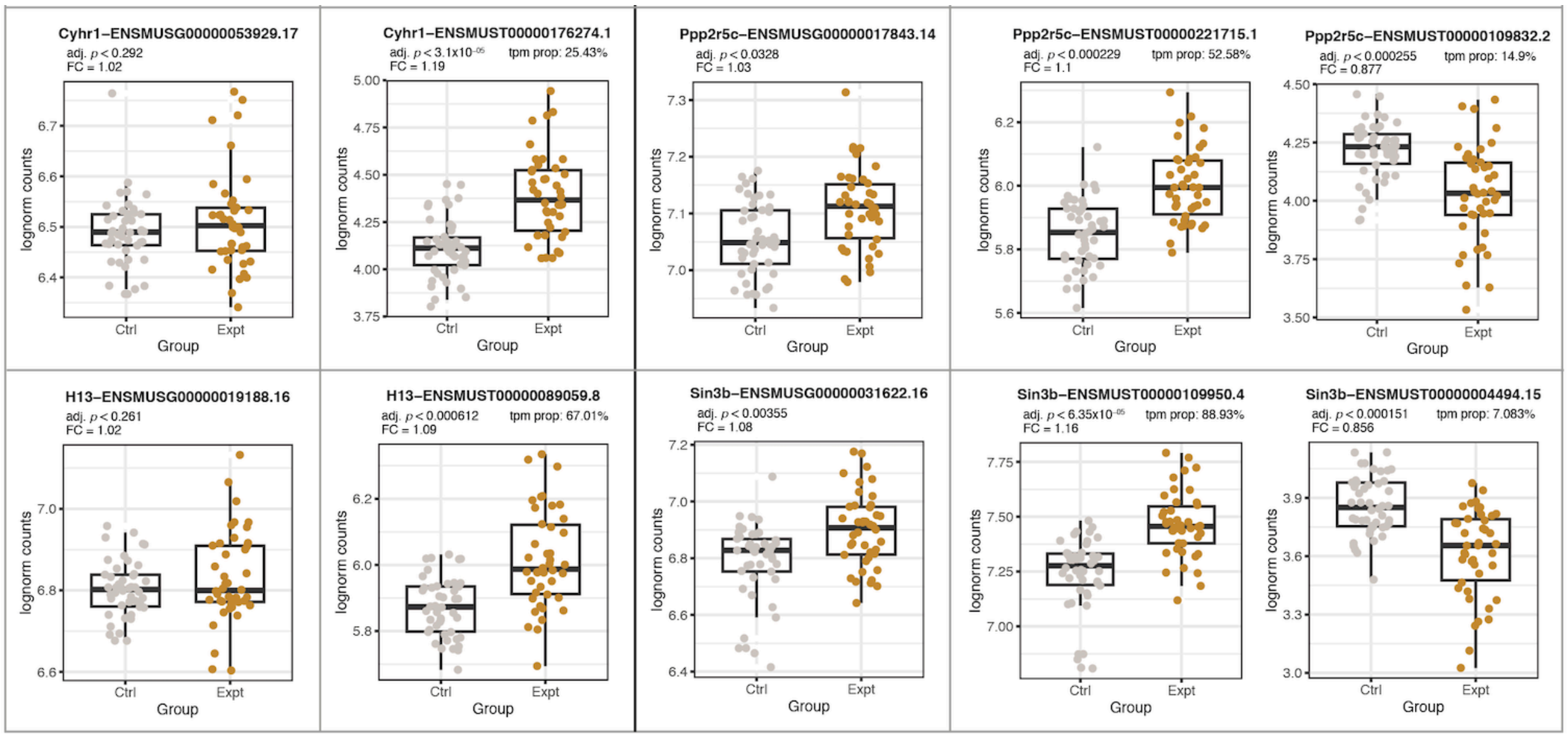

Supplementary Figure 13: **Expression of differentially expressed transcripts and their genes. A)** Plots showing the differential expression results as in **Fig. 3A** for PNE (left) and MSDP (right). The significantly differentially expressed transcripts of interest are labeled along with their corresponding gene. **B)** Box plots showing the expression of labeled transcripts (in log-TPM) and their corresponding genes (in log-CPM) for the nicotine and smoking exposure. FDR: false discovery rate; FC: fold-change; tpm prop: the proportion of total TPM of a gene that corresponds to a given transcript. Total gene TPM was obtained adding TPM of all transcripts of the gene across all samples.

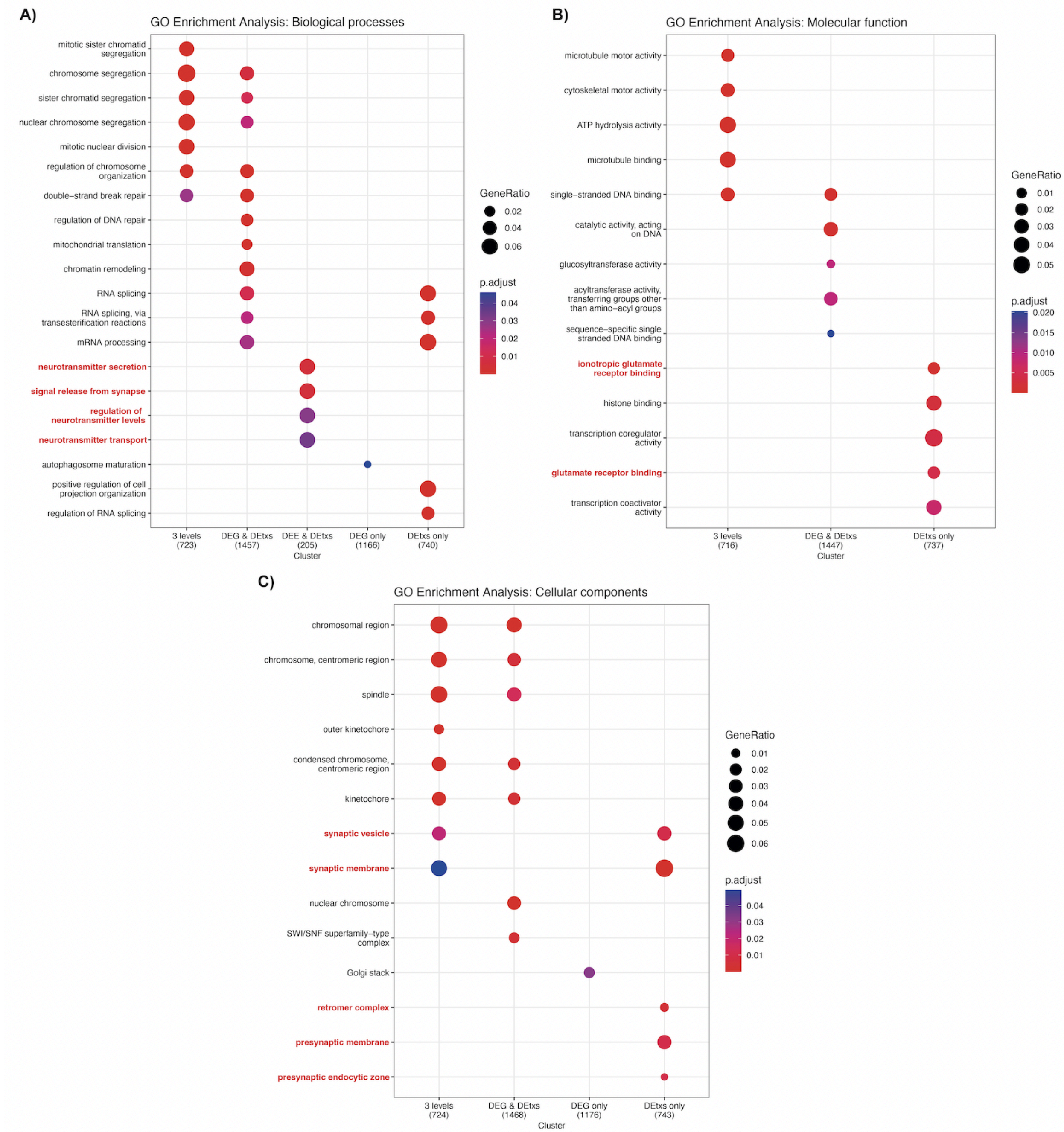

Supplementary Figure 14: **Functional enrichment analysis for genes expressing differentially expressed transcripts and exons in pup brain. A)** Biological processes, **B)** molecular functions, and **C)** cellular components significantly enriched (adjusted *p*-value<0.05) in clusters of DEGs with DE transcripts and DE exons (`3 levels`), DEGs with DE transcripts (`DEG & DEtxs`), DEGs with DE exons (`DEG & DEE`), non-DEGs with DE transcripts and DE exons (`DEE & DEtxs`), DEGs only (`DEG only`), and non-DEGs with DE transcripts only (`DEtxs only`) or DE exons only (`DEE only`), for the smoking exposure. Terms of interest appear in red. See **Fig. S9** caption for more details of these plots.

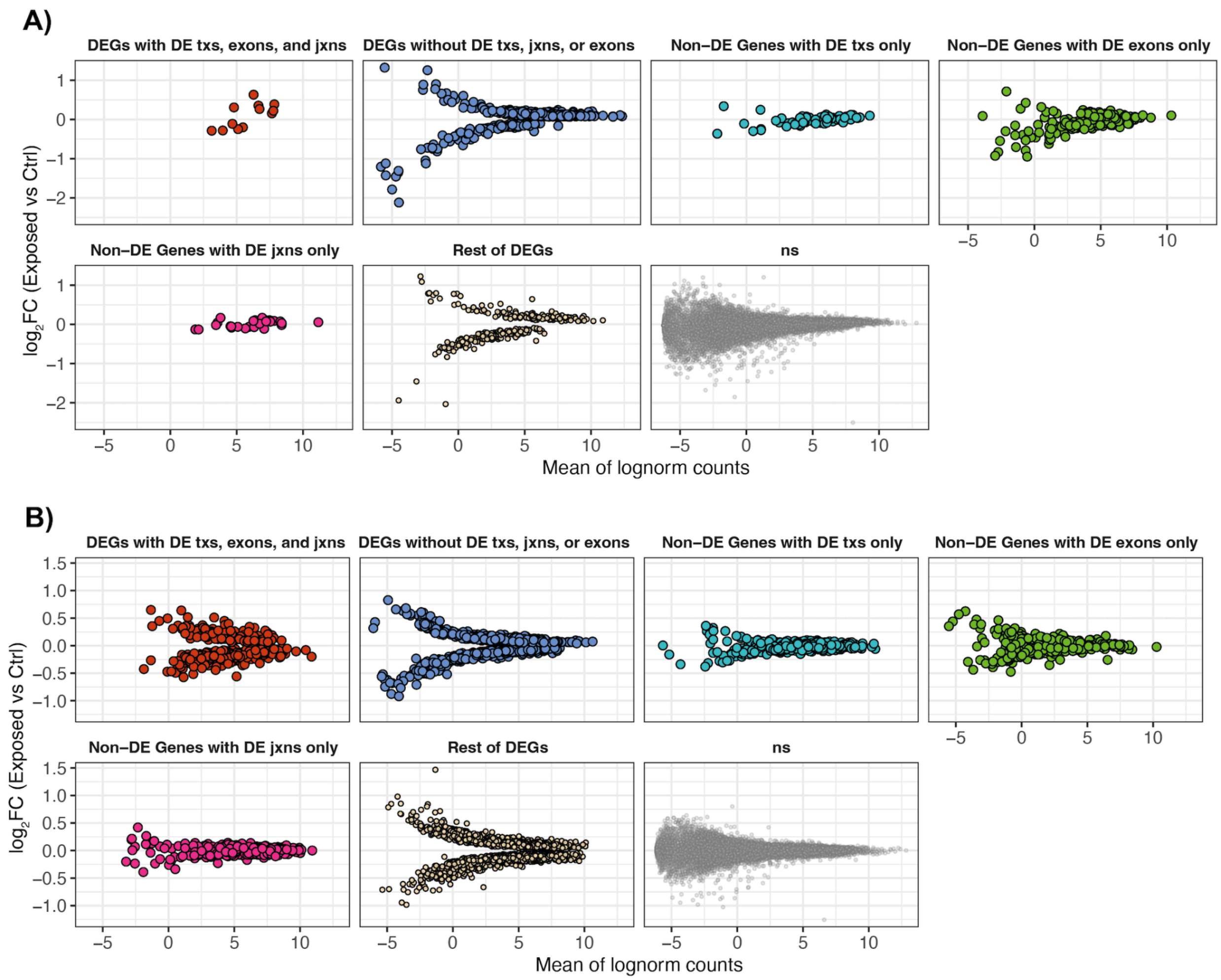

Supplementary Figure 15: MA plots of genes differentially expressed at different levels. Mean log-CPM and logFC of DEGs and non-DEGs with and without DE transcripts (txs), exons, and exon-exon junctions (jxns) for the **A)** nicotine and **B)** smoking exposure. Rest of DEGs are DEGs with two other DE features (txs and exons, txs and jxns, or exons and jxns).

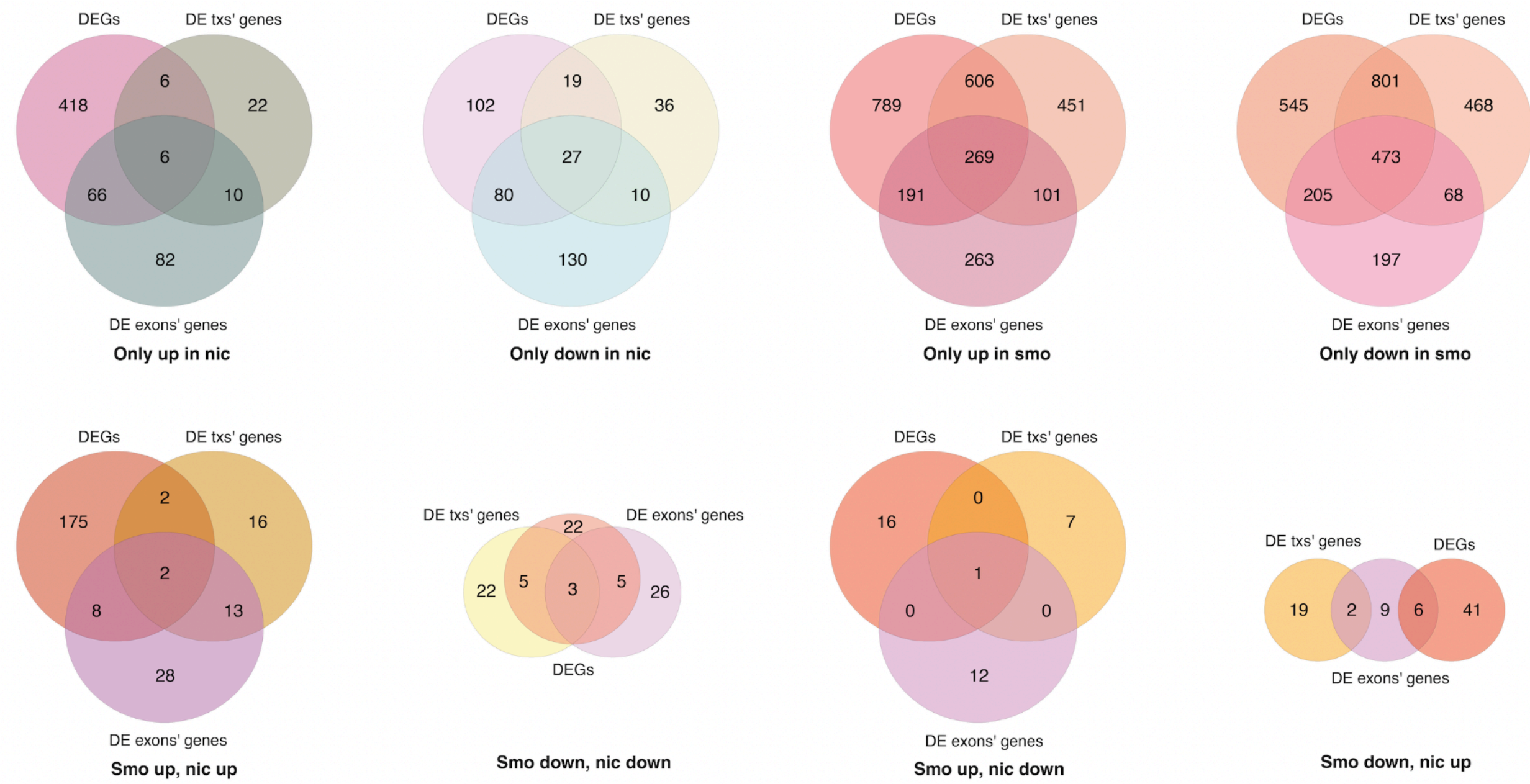

Supplementary Figure 16: **Comparison of differential expression results at gene, transcript, and exon levels in pup brain.** The overlap between DEGs and genes expressing DE exons and DE transcripts (txs) is shown across groups of up- and down-regulated features following smoking and nicotine exposures, as in **Fig. S9**.

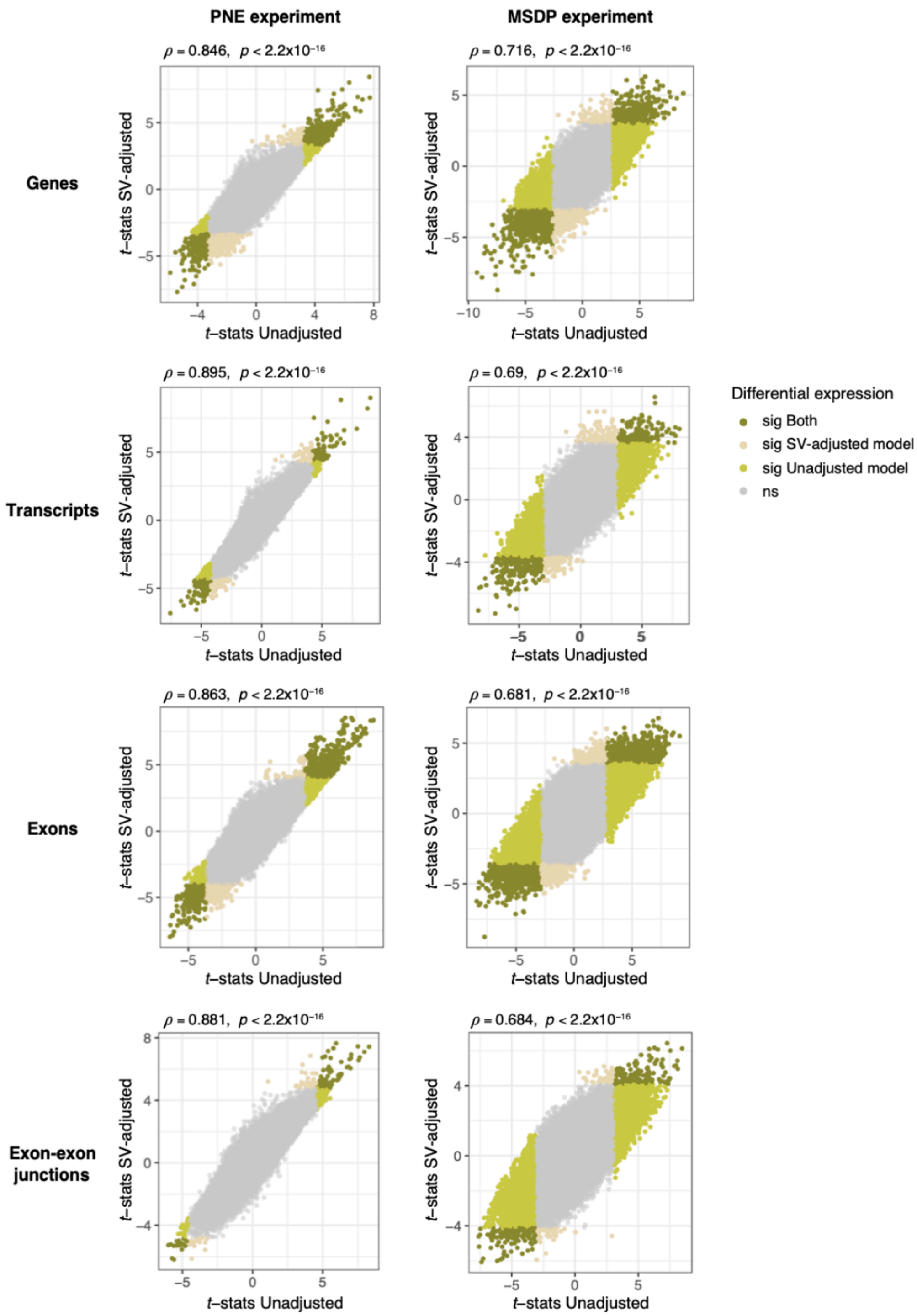

Supplementary Figure 17: **Comparison of PNE and MSDP transcriptomic effects in pup brain before and after surrogate variable adjustment.** Moderated *t*-statistics for nicotine (left) and smoking (right) prenatal exposure effects on the expression of genes, transcripts, exons, and exon-exon junctions, before and after adjustment of surrogate variables (SVs) capturing unmeasured litter- and batch-related

effects. Spearman's correlation coefficients (ϱ) between *t*-statistics and their corresponding *p*-values are shown. Related to **Table S16** and **Table S17**.

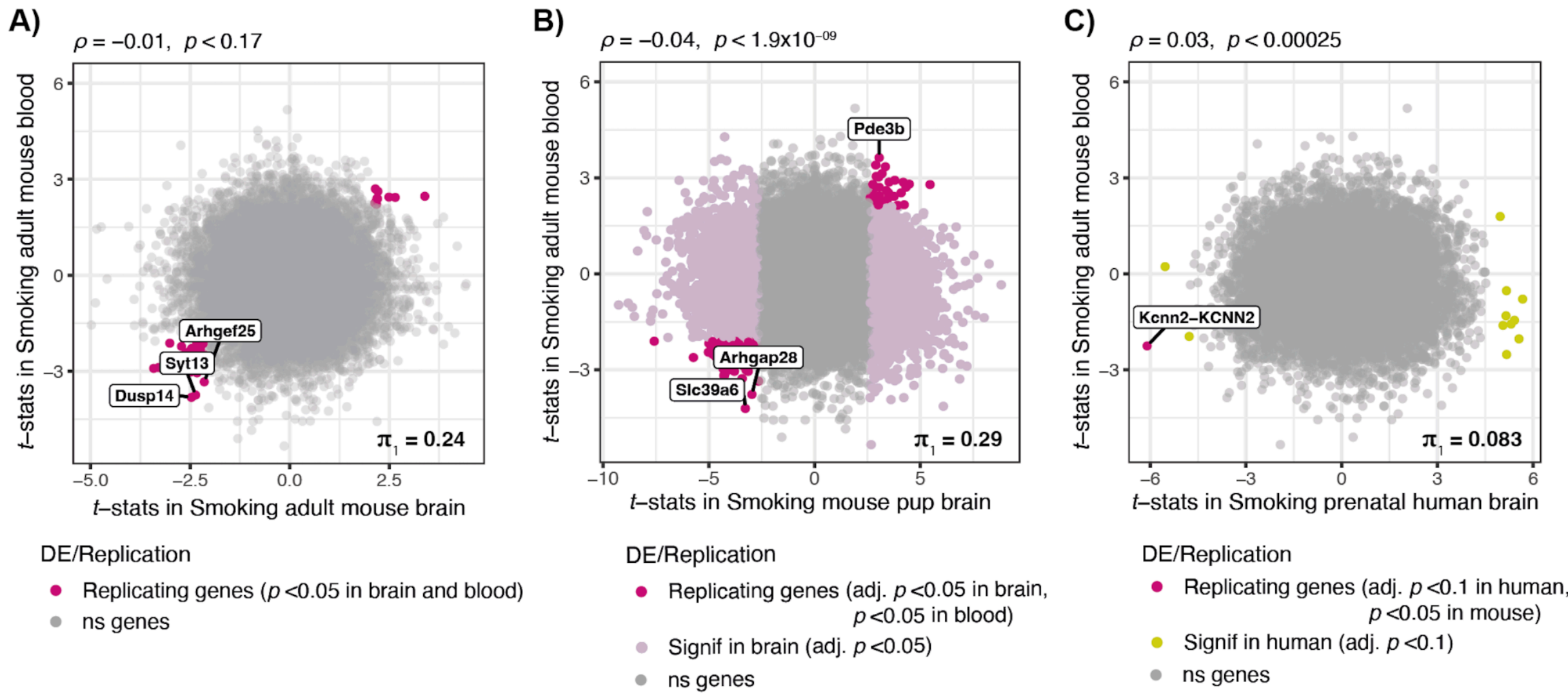

Supplementary Figure 18: Differential gene expression following smoking exposure in brain and blood. Comparison of moderated *t*-statistics for smoking exposure in adult blood vs **A)** adult brain, **B)** pup brain, and **C)** prenatal human brain. In dark pink, genes that replicate in blood (*p*-value<0.05 in blood and same logFC sign in both tissues). In light pink, MSDP-DEGs in pup brain (FDR<0.05). In yellow, DEGs for prenatal smoking in human brain (FDR<0.1). In gray, genes that were non-DEGs. The three replicating genes most significant in blood are labeled in A) and B), and the unique replicating human gene in C) is labeled together with its mouse orthologous gene name. Spearman's correlation coefficient (ϱ) and its *p*-value are shown, as well as the replication rate (π_1). Related to **Table S5** and **Table S18**.

Supplementary Material References

79. Harrow J, Denoeud F, Frankish A, Reymond A, Chen C-K, Chrast J, et al. GENCODE: producing a reference annotation for ENCODE. Genome Biol. 2006 Aug 7;7 Suppl 1(Suppl 1):S4.1-9.

80. Frankish A, Diekhans M, Ferreira A-M, Johnson R, Jungreis I, Loveland J, et al. GENCODE reference annotation for the human and mouse genomes. Nucleic Acids Res. 2019 Jan 8;47(D1):D766–73.

81. Cotto KC, Feng Y-Y, Ramu A, Skidmore ZL, Kunisaki J, Conrad DF, et al. RegTools: Integrated analysis of genomic and transcriptomic data for discovery of splicing variants in cancer. BioRxiv. 2023 Jan 9;

82. Bray NL, Pimentel H, Melsted P, Pachter L. Near-optimal probabilistic RNA-seq quantification. Nat Biotechnol. 2016 May;34(5):525–7.

83. Robinson MD, McCarthy DJ, Smyth GK. edgeR: a Bioconductor package for differential expression analysis of digital gene expression data. Bioinformatics. 2010 Jan 1;26(1):139–40.

84. Robinson MD, Oshlack A. A scaling normalization method for differential expression analysis of RNA-seq data. Genome Biol. 2010 Mar 2;11(3):R25.

85. Collado-Torres L, Jaffe AE, Burke EE. jaffelab: Commonly used functions by the Jaffe lab. 2021;

86. McCarthy DJ, Campbell KR, Lun ATL, Wills QF. Scater: pre-processing, quality control, normalization and visualization of single-cell RNA-seq data in R. Bioinformatics. 2017 Apr 15;33(8):1179–86.

87. Hoffman GE, Schadt EE. variancePartition: interpreting drivers of variation in complex gene expression studies. BMC Bioinformatics. 2016 Nov 25;17(1):483.

88. Benjamini Y, Hochberg Y. Controlling the false discovery rate: a practical and powerful approach to multiple testing. Journal of the Royal Statistical Society: Series B (Methodological). 1995 Jan;57(1):289–300.

89. Team RC. R: A Language and Environment for Statistical Computing. 2023;

90. Storey JD, Bass AJ, Robinson D. qvalue: Q-value estimation for false discovery rate control. Bioconductor. 2003 Aug 5;

91. Hoffman GE, Ma Y, Montgomery KS, Bendl J, Jaiswal MK, Kozlenkov A, et al. Sex differences in the human brain transcriptome of cases with schizophrenia. Biol Psychiatry. 2022 Jan 1;91(1):92–101.

92. Buja A, Eyuboglu N. Remarks on parallel analysis. Multivariate Behav Res. 1992 Oct 1;27(4):509–40.

93. Leek JT, Johnson WE, Parker HS, Fertig EJ, Jaffe AE, Zhang Y, et al. sva: Surrogate Variable Analysis. Bioconductor. 2017;R package(version):3.50.0.

94. Lawrence M, Huber W, Pagès H, Aboyoun P, Carlson M, Gentleman R, et al. Software for computing and annotating genomic ranges. PLoS Comput Biol. 2013 Aug 8;9(8):e1003118.

95. Wickham H. ggplot2 - Elegant Graphics for Data Analysis. 2nd ed. Cham: Springer International Publishing; 2016.

96. Huber W, Carey VJ, Gentleman R, Anders S, Carlson M, Carvalho BS, et al. Orchestrating high-throughput genomic analysis with Bioconductor. Nat Methods. 2015 Feb;12(2):115–21.

97. Appel L-M, Franke V, Bruno M, Grishkovskaya I, Kasiliauskaite A, Kaufmann T, et al. PHF3 regulates neuronal gene expression through the Pol II CTD reader domain SPOC. Nat Commun. 2021 Oct 19;12(1):6078.

98. Ka M, Kim W-Y. ANKRD11 associated with intellectual disability and autism regulates dendrite differentiation via the BDNF/TrkB signaling pathway. Neurobiol Dis. 2018 Mar;111:138–52.

99. Fowler MA, Sidiropoulou K, Ozkan ED, Phillips CW, Cooper DC. Corticolimbic expression of TRPC4 and TRPC5 channels in the rodent brain. PLoS ONE. 2007 Jun 27;2(6):e573.

100. Gualdani R, Gailly P. How TRPC channels modulate hippocampal function. Int J Mol Sci. 2020 May 30;21(11).

101. Simon R, Wiegreffe C, Britsch S. Bcl11 transcription factors regulate cortical development and function. Front Mol Neurosci. 2020 Apr 8;13:51.

102. Vázquez-Vélez GE, Zoghbi HY. Parkinson's disease genetics and pathophysiology. Annu Rev Neurosci. 2021 Jul 8;44:87–108.

103. Li M-J, Yan S-B, Chen G, Li G-S, Yang Y, Wei T, et al. Upregulation of CCNB2 and its perspective mechanisms in cerebral ischemic stroke and all subtypes of lung cancer: A comprehensive study. Front Integr Neurosci. 2022 Jul 19;16:854540.

104. Deng JM, Behringer RR. An insertional mutation in the BTF3 transcription factor gene leads to an early postimplantation lethality in mice. Transgenic Res. 1995 Jul;4(4):264–9.

105. Cabrera JR, Lucas JJ. MAP2 splicing is altered in huntington's disease. Brain Pathol. 2017 Mar;27(2):181–9.

106. Sathasivam K, Neueder A, Gipson TA, Landles C, Benjamin AC, Bondulich MK, et al. Aberrant splicing of HTT generates the pathogenic exon 1 protein in Huntington disease. Proc Natl Acad Sci USA. 2013 Feb 5;110(6):2366–70.

107. Guilarte TR, Burton NC, Verina T, Prabhu VV, Becker KG, Syversen T, et al. Increased APLP1 expression and neurodegeneration in the frontal cortex of manganese-exposed non-human primates. J Neurochem. 2008 Jun;105(5):1948–59.

108. Wilson R, Geyer SH, Reissig L, Rose J, Szumska D, Hardman E, et al. Highly variable penetrance of abnormal phenotypes in embryonic lethal knockout mice. [version 2; peer

review: 3 approved]. Wellcome Open Res. 2016;1:1.

109. Gonzàlez-Porta M, Frankish A, Rung J, Harrow J, Brazma A. Transcriptome analysis of human tissues and cell lines reveals one dominant transcript per gene. Genome Biol. 2013 Jul 1;14(7):R70.

110. Frankish A, Diekhans M, Jungreis I, Lagarde J, Loveland JE, Mudge JM, et al. GENCODE 2021. Nucleic Acids Res. 2021 Jan 8;49(D1):D916–23.
