# Supplementary material for: Molecular impact of nicotine and smoking exposure on the developing and adult mouse brain
Source: Addict Neurosci. Author manuscript; Available in PMC 2026 Oct 15. (PMC13662931; doi:10.1016/j.addicn.2026.100270)
Supplement: Supplementary file 2 [file NIHMS2211658-supplement-12.pdf]

Supplementary File 1: Additional DTE, DEE, and DJE results

Findings beyond gene level for gestational nicotine and smoking exposure on developing frontal cortex

DTE results

In addition to the highly concordant DGE and DTE results observed for prenatal nicotine and smoking exposure (**Fig. 3A**), PNE specifically affected transcripts of *Phf3, Ankrd11*, *Trpc4*, *Bcl11a*, and *Scaf11,* which were not significantly differentially expressed at the gene level but are all implicated in brain development (97–101) (97–102) (**Fig. S13**, **Table S8**). Moreover, consistent with DGE results, MSDP led to the downregulation of *Top2a* and *Ccnb2* transcripts, and upregulation of *Mt2* transcripts (**Fig. S13**, **Table S9**). Importantly, the ortholog of *Ccnb2* in human has been shown to promote cerebral ischemic stroke and lung cancer by interacting with *TOP2A* (103), pointing to potentially conserved regulatory mechanisms across species and disease contexts.

Furthermore, as observed for PNE, MSDP also affected transcript expression in genes not detected as differentially expressed, including *Btf3*, *Cyhr1*, *H13*, and *Srsf6* (**Fig. S13**, **Table S9**). Of these, *Btf3* is essential for in utero embryonic development (104), whereas the splicing factor gene *Srsf6* has target genes involved in brain organogenesis and has been implicated in missplicing events that lead to Huntington's disease (105,106). *Cyhr1* has been shown to respond to chronic manganese exposure linked to neurodegenerative changes in the frontal cortex (107), and *H13* plays a critical role in embryonic development and brain morphology (108). Together, these results highlight the added sensitivity of transcript-level analyses in capturing regulatory changes that may be masked in gene-level summaries, particularly for genes with complex isoform structure.

Interestingly, transcript-level analyses revealed differentially expressed transcripts regulated in the opposite direction to their parent gene, as observed for *Dgcr8* in PNE (**Fig. S13**, **Table S8**).
In addition, several DEGs exhibited both up- and downregulated transcripts, including *Pnsir* and *Dcun1d5* for PNE, and *Meaf6*, *Ivns1abp*, *Morf4l2*, *Sin3b*, and *Ppp2r5c* for MSDP (**Fig. S13**, **Table S8**, **Table S9**). Consistent with previous studies indicating that genes often express dominant transcript isoforms (109), transcripts regulated in the same direction as the gene accounted for a greater proportion of total gene expression than those regulated in the opposite direction (**Fig. S13**). These patterns could be indicative of differential transcript usage (DTU), where both overall gene expression and splicing dynamics are altered, leading to shifts in the relative abundance of transcript isoforms between conditions. Analyses of transcript expression proportions relative to total gene expression will enable the inference of these events.

DJE results

DJE analysis was performed to find novel splice isoforms, i.e., that are not annotated in GENCODE M25 (79,110). All differentially expressed exon-exon junctions except two for MSDP

were novel, with at least one unannotated splice site or having an unknown combination of donor and acceptor sites. Of DE junctions, 5 and 201 for PNE and MSDP, respectively, were fully novel, with both splice sites unknown and without assigned gene. For these novel junctions their immediate following and preceding genes, as well as their nearest overlapping neighbor gene were located (**Table S20**). The latter genes had a bigger overlap with the identified DEGs at the gene, transcript, and exon levels (**Fig. S19**), further supporting these nearest genes as bearers of potentially new isoforms, compared to immediate upstream and downstream genes. Detailed analyses of these results are required to further explore their plausibility and potential functional relevance.

Collectively, agreement was found between DGE, DTE, DEE, and DJE results for both PNE and MSDP (**Fig. 3C**). Overall, for more highly expressed DEGs, DE transcripts, exons, and exon-exon junctions were detected (**Fig. S15**), and such features were regulated in the same direction (**Fig. S16**). However, many DEGs only exhibited significant DE signal at the gene level. One could hypothesize that these DEGs have low expression levels and thus, not enough reads to properly quantify their exons, transcripts, and junctions, but that was not necessarily the case. While several DEGs with low mean expression have no other DE features (blue points in **Fig. S15** with mean lognorm counts < 0), not all DEGs with no DE features have low mean expression–they have smaller logFCs (blue points in **Fig. S15**). Furthermore, there were DE features from non-DE genes, where the high expression of such genes enabled their detection (**Fig. S15**). A plausible explanation for the presence of DE exons from non-DEGs or lowly expressed genes is that during exon quantification, reads mapping to regions shared by overlapping exons are assigned to all, artificially increasing their expression (see **Supplementary Materials and Methods: Read alignment and expression quantification**).

Additional caveats should be considered when interpreting results at the exon and exon-exon junction levels. Because exons may be shared across multiple transcripts, read assignment may be ambiguous. Depending on the analytical approach, reads may be partially or fully attributed to multiple features, as previously described (18). Consequently, exon-level expression does not necessarily reflect the abundance of the transcripts that contain those exons. In contrast, transcript-level estimates are derived using methods that probabilistically resolve read-mapping ambiguity across shared regions (82).

These differences in quantification frameworks mean that exon-level and transcript-level estimates are not directly comparable, and exon-level results should be interpreted with caution when making inferences about transcript expression. Similar considerations apply to exon-exon junction expression estimates. Future analyses incorporating transcript assembly approaches may help better resolve isoform structure and identify previously uncharacterized, condition- or context-specific transcript isoforms.

Additional Supplementary Figures

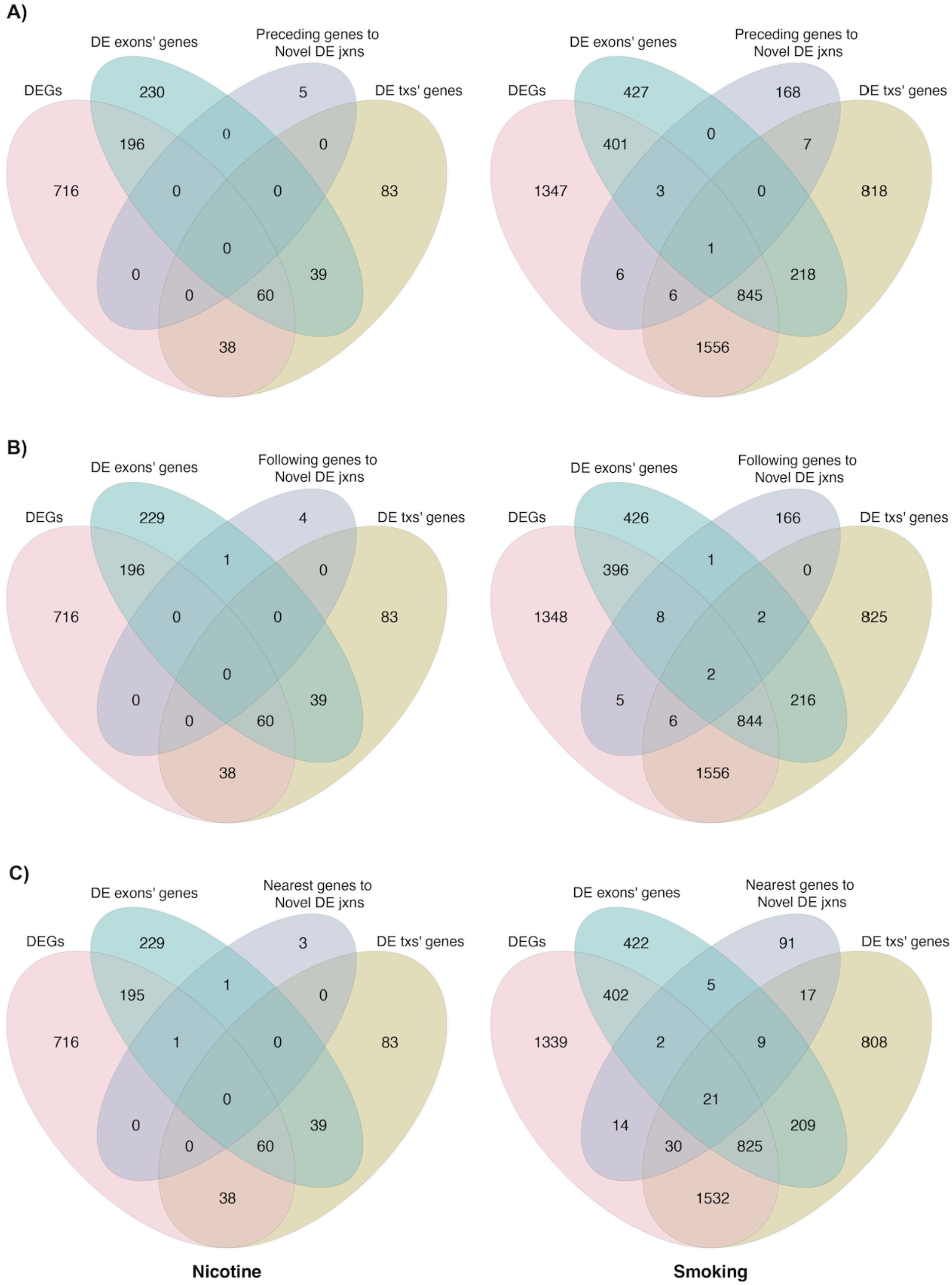

Supplementary Figure 19: Overlap between genes differentially expressed at different feature levels and genes associated with differentially expressed exon-exon junctions in pup brain.

Overlap between DEGs, genes expressing DE exons, genes expressing DE transcripts, and **A)** the preceding, **B)** following, and **C)** nearest genes annotated to DE novel exon-exon junctions without assigned gene, for PNE (left) and MSDP (right).
